# NAC Pre-Administration Prevents Cardiac Mitochondrial Bioenergetics, Dynamics, Biogenesis, and Redox Alteration in Folic Acid-AKI-Induced Cardio-Renal Syndrome Type 3

**DOI:** 10.3390/antiox12081592

**Published:** 2023-08-10

**Authors:** Belén Cuevas-López, Edgar Ignacio Romero-Ramirez, Fernando E. García-Arroyo, Edilia Tapia, Juan Carlos León-Contreras, Alejandro Silva-Palacios, Francisco-Javier Roldán, Omar Noel Medina Campos, Luz Hernandez-Esquivel, Alvaro Marín-Hernández, José Guillermo Gonzaga-Sánchez, Rogelio Hernández-Pando, José Pedraza-Chaverri, Laura Gabriela Sánchez-Lozada, Omar Emiliano Aparicio-Trejo

**Affiliations:** 1 Department of Cardio-Renal Physiology, National Institute of Cardiology Ignacio Chávez, Mexico City 14080, Mexico; bcuevasl1400@alumno.ipn.mx (B.C.-L.); 314322023@quimica.unam.mx (E.I.R.-R.); enrique.garcia@cardiologia.org.mx (F.E.G.-A.); edilia.tapia@cardiologia.org.mx (E.T.); jose.gonzaga@cardiologia.org.mx (J.G.G.-S.); laura.sanchez@cardiologia.org.mx (L.G.S.-L.); 2 Experimental Pathology Section, National Institute of Medical Sciences and Nutrition “Salvador Zubirán”, Mexico City 14000, Mexico; carlos.leonc@incmnsz.mx (J.C.L.-C.); rogelio.hernandezp@incmnsz.mx (R.H.-P.); 3 Department of Cardiovascular Biomedicine, National Institute of Cardiology Ignacio Chávez, Mexico City 14080, Mexico; alejandro.silva@cardiologia.org.mx; 4 Outpatient Department, National Institute of Cardiology Ignacio Chávez, Mexico City 14080, Mexico; roldan@cardiologia.org.mx; 5 Department of Biology, Faculty of Chemistry, National Autonomous University of Mexico, Mexico City 04510, Mexico; mconoel@unam.mx (O.N.M.C.); pedraza@unam.mx (J.P.-C.); 6 Department of Biochemistry, National Institute of Cardiology Ignacio Chávez, Mexico City 14080, Mexico; maria.esquivel@cardiologia.org.mx (L.H.-E.); alvaro.marin@cardiologia.org.mx (A.M.-H.)

**Keywords:** cardio-renal syndrome type 3, folic acid-induced cardio-renal damage, NAC and mitochondria, mitochondrial ROS production, mitochondrial dynamic, biogenesis

## Abstract

The incidence of kidney disease is increasing worldwide. Acute kidney injury (AKI) can strongly favor cardio-renal syndrome (CRS) type 3 development. However, the mechanism involved in CRS development is not entirely understood. In this sense, mitochondrial impairment in both organs has become a central axis in CRS physiopathology. This study aimed to elucidate the molecular mechanisms associated with cardiac mitochondrial impairment and its role in CRS development in the folic acid-induced AKI (FA-AKI) model. Our results showed that 48 h after FA-AKI, the administration of N-acetyl-cysteine (NAC), a mitochondrial glutathione regulator, prevented the early increase in inflammatory and cell death markers and oxidative stress in the heart. This was associated with the ability of NAC to protect heart mitochondrial bioenergetics, principally oxidative phosphorylation (OXPHOS) and membrane potential, through complex I activity and the preservation of glutathione balance, thus preventing mitochondrial dynamics shifting to fission and the decreases in mitochondrial biogenesis and mass. Our data show, for the first time, that mitochondrial bioenergetics impairment plays a critical role in the mechanism that leads to heart damage. Furthermore, NAC heart mitochondrial preservation during an AKI event can be a valuable strategy to prevent CRS type 3 development.

## 1. Introduction

The incidence of acute kidney injury (AKI), a group of pathological syndromes defined by the deterioration of renal functions in a short period, is currently increasing worldwide, with more than 13 million people affected annually by AKI, far exceeding breast cancer and heart failure [1,2,3]. Between 10 and 15% of intensive care unit patients present AKI episodes [2], and the global mortality from AKI has remained high for the past 50 years [3] because AKI is associated with adverse short-term outcomes and adverse long-term effects on survival [1]. One or several episodes of AKI favors the development of chronic kidney disease (CKD) [4,5], with high mortality rates [6]. Furthermore, renal dysfunction alone is insufficient to explain the high mortality rate in AKI patients because the damage to remote organs, like the heart, caused by AKI also contributes to poor recovery [1,7,8]. Current evidence has shown that AKI causes the release into the bloodstream of soluble mediators, known as cardio-renal connectors, such as inflammatory molecules and renin-angiotensin system components, together with uremia and electrolyte and hemodynamic unbalance, resulting in the spread of damage to peripheral tissues, such as the heart, favoring cardio-renal syndrome (CRS) development [9,10,11]. Type 3 CRS is characterized by an acute worsening of renal function that triggers cardiac dysfunction, a common cause of death in AKI patients [10,12]. Although recently CRS pathology has been extensively studied, the molecular pathways connecting renal to cardiac damage are not wholly deciphered [8,12,13].

On the other hand, mitochondrial bioenergetics and redox dysfunction have recently emerged as central axes in developing AKI pathological processes like tubular damage, hemodynamic alterations, inflammation, cellular death, oxidative stress, and fibrosis [14,15,16,17,18]. Likewise, growing evidence suggests that renal mitochondrial alterations participate in the progression of several types of CKD [19,20,21,22,23]. The kidney and heart are energy-demanding organs that depend highly on mitochondrial bioenergetics [19,24,25]. Additionally, to be involved in crucial metabolic processes, cell death, and inflammation, mitochondria damage is closely related to the development of cardiovascular diseases and CRS [13,26,27]. Consequently, it was recently shown that CKD triggers mitochondrial bioenergetic impairment in organs such as the heart [13,27,28] and skeletal muscle [29], favoring the spread of damage in these tissues. Nevertheless, heart mitochondria impairment related to the molecular pathways that trigger CRS type 3 is poorly understood [7,12].

The folic acid (FA)-induced AKI (FA-AKI) model has been widely used to study the pathophysiology of AKI [30,31] because it recreates the pathology reported in patients [32] and is highly reproducible [30,31]. The FA high concentrations induce cell death (especially in the proximal tubule), tubular obstruction, hemodynamic alterations, hyperuricemia, cytokine release, inflammation, fibrosis, oxidative stress, mitochondrial bioenergetics, and redox alterations in the kidney that lead to CKD development [15,20,30,33]. Furthermore, previous reports showed that FA-AKI triggers the release into the bloodstream of proinflammatory cytokines and uric acid from the kidney [20,34,35,36,37], leading to cardiac inflammation and cardiomyocyte apoptosis, favoring CRS type 3 development [38]. Additionally, we previously demonstrated that intraperitoneal FA administration (300 mg/kg) triggers in kidney mitochondria the decrease in glutathione (GSH) levels and the higher S-glutathionylation removal activity of mitochondrial glutaredoxin (Grx), leading to complex I (CI) activity reduction [15]. The decrease in renal mitochondrial oxidative phosphorylation (OXPHOS) capacity and coupling induce the loss of mitochondrial membrane potential (ΔΨm) and increase mitochondrial hydrogen peroxide (H_2_O_2_) production in the kidney, favoring AKI development [15]. However, until now, it is unknown if cardiac mitochondria alterations in this model exist and their participation in the CRS.

N-acetyl-cysteine (NAC) is a precursor of GSH [39,40] that regulates mitochondrial function by GSH levels [15,41,42]. NAC has been employed to prevent mitochondrial bioenergetic alterations in kidney damage models [39,43] as well as renal damage in cardiac surgery and contrast-induced nephropathy [40]. We previously demonstrated that NAC pre-administration prevented renal mitochondrial bioenergetics, redox state, and dynamics alterations in FA-AKI [15]. Furthermore, NAC prevents the long-term deterioration of renal mitochondrial function, avoiding CKD development [44]. However, NAC effects on the possible cardiac mitochondrial alterations have not been evaluated in type 3 CRS. Therefore, our objective was to elucidate and characterize the cardiac mitochondrial impairment and its role in CRS type 3 development in rats with the FA-AKI model. Our results show, for the first time, that the FA-AKI model induces cardiac mitochondrial impairment. Furthermore, NAC protected the cardiac mitochondrial OXPHOS and ΔΨm by CI activity and GSH preservation, avoiding the mitochondrial dynamics shift to fission and the decreases in mitochondrial biogenesis and mass in the heart induced by FA-AKI. This cardiac mitochondrial protection was associated with NAC capacity to prevent heart inflammation and oxidative stress, thus preventing CRS type 3 development.

## 2. Materials and Methods

### 2.1. Reagents

Adenosine 5′-diphosphate sodium salt (ADP), adenosine 5′-triphosphate sodium salt (ATP), Amplex Red, antimycin A, ammonium chloride (NH_4_Cl), L-arginine, fat-free bovine serum albumin (BSA), β-mercaptoethanol, bromophenol blue, 1-Chloro-2,4-dinitrobenzene (CDNB), carbonyl cyanide m-chlorophenylhydrazone (CCCP), catalase from bovine liver, cytochrome *c* from equine heart, coenzyme A (CoA), D-(+)-glucose, D-mannitol, decylubiquinone (DUB), 2,6-dichlorophenolindophenol sodium salt hydrate (DCPIP), 5,5′-dithio-bis-(2-nitrobenzoic acid) (DTNB), dithiothreitol (DTT), GSH, glutathione disulfide (GSSG), GSH peroxidase (GPx) lyophilized powder, 5-thio-2-nitrobenzoic acid (TNB) ethylene glycol-bis(2-aminoethyl ether)-N,N,N′,N′-tetraacetic acid (EGTA), FA, glucose-6-phosphate dehydrogenase (G6PDH), glutathione reductase (GR), glutamic acid, glutaraldehyde, glycerol, hexokinase, 4-(2-hydroxyethyl)-1-piperazineethanesulfonic acid (HEPES), horseradish peroxidase (HRP), K-lactobionate, manganese (II) chloride (MgCl_2_) tetrahydrate, lead citrate, malic acid, NAC, β-Nicotinamide adenine dinucleotide phosphate reduced (NADPH), and oxidized (NADP^+^), β-Nicotinamide adenine dinucleotide reduced (NADH), and oxidized (NAD^+^), nitro blue tetrazolium (NBT), osmium tetroxide, potassium cyanide (KCN), rotenone, safranin O, sodium azide, sodium succinate dibasic, sodium phosphate dibasic (Na_2_HPO_4_), sodium phosphate monobasic (NaH_2_PO_4_), sodium glutamate, sodium L-ascorbate, sodium chloride (NaCl), sodium fluoride (NaF), sodium orthovanadate (Na_3_VO_4_), sodium malate, sodium deoxycholate, sodium dodecyl sulfate (SDS), superoxide dismutase (SOD) bovine, phenylmethanesulfonyl fluoride (PMSF), paraformaldehyde, sucrose, taurine, tetramethyl-p-phenylenediamine (TMPD), trizma (Tris), trizma-hydrochloride (Tris-HCl), triton X-100, thiamine pyrophosphate, tween, uranyl acetate, 2-oxoglutarate, 2-vinylpyridine (2-VP), and creatine kinase (CK) activity assay kit were purchased from Sigma-Aldrich (St. Louis, MO, USA). Commercial kits from Spinreact (Girona, Spain) were used to measure blood urea nitrogen (BUN), plasma creatinine, aspartate aminotransferase (AST), and alanine aminotransferase (ALT). The sedative sodium pentobarbital (SedalphorteMR) was purchased from Salud y Bienestar Animal S.A. de C.V (Mexico City, Mexico). Sodium bicarbonate (NaHCO_3_), H_2_O_2_, ethyl alcohol, ethylenediaminetetraacetic acid disodium salt dihydrate (EDTA), and potassium hydroxide (KOH) were purchased from JT Baker (Xalostoc, Edo. Mexico, Mexico). Non-fat dry milk and antibodies against β-Actin, brain natriuretic peptide (BNP), interleukin (IL)-6, troponin C (Trop C), dynamin-related protein 1 (DRP1), mitochondrial fission 1 protein (FIS1), optic atrophy 1 (OPA1), mitofusin1 (MFN1) and 2 (MFN2), nuclear respiratory factor 1 (NRF1) and 2 (NRF2), sirtuin 1 (SIRT1) and 3 (SIRT3), PTEN-induced putative kinase 1 (PINK1), tubulin and phosphatase and tensin homolog (PTEN)-induced putative kinase 1 (PINK1) were purchased from Santa Cruz Biotechnology (Dallas, TX, USA). Antibody against BCL2 interacting protein 3 (BNIP3) was purchased from Cell Signaling (Danvers, MA, USA). Antibodies against voltage dependence anion channel 1 (VDAC1), peroxisome proliferator-activated receptor gamma coactivator 1-alpha (PGC-1α), mitochondrial ATP-dependent Lon protease 1 (LONP1), ATP synthase subunit 5A (ATP5A), malondialdehyde (MDA), carnitine palmitoyltransferase-1 (CPT1), peroxisome proliferator-activated receptor alpha (PPARα) and gamma (PPARγ), and 4-hydroxynonenal (4HNE), as well as tumor necrosis factor (TNF)-alpha ELISA kit and cardiolipin assay kit were purchased from Abcam (Cambridge, MA, USA). Protease inhibitor cocktail phosphoenolpyruvate (PEP), pyruvate kinase (PYK)/lactate dehydrogenase (LDH), and glutamate dehydrogenase were purchased from Roche Applied Science (Mannheim, Germany). Succinyl CoA synthetase was purchased from Megazyme (Bray, Ireland). Glutaredoxin (Grx) fluorescent activity assay kit was from Cayman Chemical (Ann Arbor, MI, USA).

### 2.2. Experimental Design

The experimental protocol was approved by the Institutional Animal Care Committee (Comité Interno para el Cuidado y de Uso y de Animales de Laboratorio, CICUAL) at the National Institute of Cardiology Ignacio Chávez (INC/CICUAL/013/2021) and was conducted according to Mexican Official Norm Guides for the use and care of laboratory animals (NOM-062-ZOO-1999) and the disposal of biological residues (NOM-087-SEMARNAT-SSA1-2002). Male Wistar rats (4 groups) with an initial body weight between 250 and 300 g were employed (n = 5–6 per group). Group 1: Vehicle, animals were injected with 300 mM NaHCO_3_. Group 2: FA, animals received an intraperitoneal dose of FA (300 mg/kg body weight) dissolved in 300 mM NaHCO_3_ every 48 h on days 1, 3, and 5. Group 3: NAC + FA animals were pre-treated with NAC (300 mg/kg) 2 h before each FA administration (3 times: days 1, 3, 5). Group 4: NAC, animals received the NAC doses (3 times: days 1, 3, 5) before vehicle administration. The analysis was conducted on day 7, 48 h after the last FA administration. The doses used for each compound followed our previous report [15,22]. The rats were housed in a temperature-controlled environment with a 12–12 h light-dark cycle and maintained with water and food *ad libitum.* Animals were anesthetized with sodium pentobarbital (90 mg/kg). Blood was obtained from the abdominal aorta, and plasma was separated and stored at 4 °C to determine BUN and creatinine levels as markers of renal function.

### 2.3. Renal, Liver, and Heart Damage Markers

Renal damage markers creatinine and BUN levels and liver damage markers ALT and AST were assessed in plasma by commercial kits from Spinreact (Girona, Spain) following the manufacturer’s instructions [17]. Meanwhile, heart damage was evaluated by the determination of the CK activity in plasma by commercial kits purchased from Sigma-Aldrich (St. Louis, MO, USA) as well as the BNP, IL-6, and Trop C levels by Western blot (WB), and TNF-α by ELISA kit in heart homogenates using the methodology below described.

### 2.4. Kidney and Heart Histology and Immunohistochemistry

For hematoxylin/eosin (H&E) stains, both organs, kidneys, and hearts were washed in PBS, cut in two halves, and fixed in paraformaldehyde-glutaraldehyde solution (4–1.5%, respectively), pH = 7.2. Then, 3 mm wide sections were dehydrated, embedded in paraffin, and 5 μm sections were obtained. For H&E staining, the slides were deparaffinized at 50 °C, hydrated with graduated alcohols in descending order, Harris Hematoxylin (Sigma-Aldrich ID 24895879) was used with lithium carbonate to intensify color, Eosin Y (Sigma-Aldrich SKU 230251-25G) was used, the slides were dehydrated and mounted in Entellan resin (Merck 107961, Darmstadt, Germany) for observation. Additionally, 1 mm width slices were dehydrated, embedded in paraffin, and 5 μm sections were obtained for the corresponding immunohistochemistry and were mounted on positively charged slides to ensure tissue adherence. Then, citrate buffer 0.01 M, pH 6.2, and immersion in a 95 °C water bath were used for heat-induced epitope retrieval. Endogenous peroxidase was quenched using a rabbit polydetector peroxidase blocker (Bio SB). Two hundred microliters of rabbit anti-mouse polyclonal antibody anti-TNF-alpha and BAX (Santa Cruz, sc-20672 1:250 dilution) were used for slide incubation, the slides were incubated for 30 min with the biotinylated mouse/rabbit immunodetection, and HRP Label rabbit poly-detector (Bio SB), and bound antibodies were detected with the Rabbit Polydetector DAB Kit (Bio SB, Santa Barbara, CA, USA). Quantification of areas with immunolabeling for TNF- alpha and BAX was performed using the open source image processing software Image J (2.0.0-rc-43/1.52n), deconvolution was performed with the DeconvolutionLab plugins (BIG-EPFL) for Image J.

### 2.5. Evaluation of Cardiac Function by Echocardiography

Two-dimensional images of the cardiac chamber were obtained from short-axis views of the left ventricle (LV) at the papillary muscle levels in rats from different groups using a SONOS 550 echocardiographer (Koninklijke Phillips Electronics, Eindhoven, The Netherlands) with a 12 MHz transducer. Briefly, rats were anesthetized with a low dose of sodium pentobarbital (1.9 mg/100 g body weight, intraperitoneally). LV internal diameter, LV posterior wall thickness, fractional shortening (FS), and ejection fractions (EF) were calculated from the LV dimensions at the end-systolic (LVDs) and diastolic (LVDd), using the following formulas: %EF = Y + [(100 − Y) × 0.15], where Y = [(LVDd2 − LVDs2/LVDd2) × 100] and %FS = [(LVDd − LVDs/LVDd) × 100] according to our previous report [45]. At the end of the evaluation, the animals were allowed to recover for a few days before being euthanized.

### 2.6. Protein Extraction and WB

The protein extraction was performed in radioimmunoprecipitation (RIPA) buffer (2 mM EDTA, 1 mM EGTA, 150 mM NaCl, 5 mM NaF, 40 mM Tris-HCl, 1 mM Na_3_VO_4_, 1 mM PMSF, 0.5% sodium deoxycholate, 0.1% SDS, pH 7.6 with protease inhibitor cocktail) as previously described [15]. Lowry method was employed to determine protein concentration. The samples were diluted in 5X Laemmli buffer (60 mM Tris-HC l2% SDS, 10% glycerol, 5% β-mercaptoethanol, 0.01% bromophenol blue, pH = 6.8), denaturalized, and run in SDS-PAGE electrophoresis polyvinylidene fluoride (PVDF) membranes were used for transference, membrane blocking, primary and secondary antibody incubation was performed as a previously described [15]. Chemiluminescent protein bands were using a ChemiDoc XRS+ Imaging Systems (BIO-RAD, Alfred Nobel Drive, Hercules, CA, USA) and analyzed using the Image Lab 6.1 (BIORAD, Alfred Nobel Drive, Hercules, CA, USA) software.

### 2.7. Isolation of Heart Mitochondria

After sacrifice, the heart was cooled by immersion in isolation buffer (225 mM D-mannitol, 75 mM sucrose, 1 mM EDTA, 5 mM HEPES, 0.1% BSA, pH = 7.4) at 4 °C and then cut into small pieces. Mitochondria were isolated from the whole heart mass, tissues were homogenized in a glass Potter-Elvehjem with a TeflonVR pestle in the same buffer, and mitochondria were obtained by differential centrifugation [22,46]. The pellet was resuspended in 120 µL of BSA-free isolation buffer, and the total mitochondrial protein was measured by the Lowry method [22,46].

### 2.8. Mitochondrial Membrane Potential (ΔΨm)

The changes in ΔΨm at 37 °C were measured using 5 µM safranin O as previously described [15] using a Synergy-Biotek microplate reader (Biotek Instruments, Winooski, VT, USA). CI-linked substrate respiration was achieved using a mix of pyruvate, malate, and glutamate (5:2:10 mM), meanwhile complex II (CII)-linked respiration was achieved by the addition of the mix succinate rotenone (10 mM: 0.5 µM). State 3 (S3) was determined in the presence of a 2.5 mM ADP, and state 4 was induced by 2.5 μM oligomycin addition (S4o). Unspecific interactions were determinate by 5 μM CCCP addition and used as a correction parameter. Results were expressed as the changes in arbitrary units of fluorescence (AUF) of safranin O and normalized per milligram of protein (AUF/mg of protein).

### 2.9. The Activity of Mitochondrial Respiratory Complexes

Respiratory complexes activity was measured as previously described [22]. The CI activity was evaluated (at 600 nm) by the decrease in the DCPIP absorbance (proportional to the CI activity) using NADH as a substrate and DUb as a mobile element, meanwhile rotenone was used as a specific inhibitor to determinate unspecific correction. The CII activity was also evaluated by the decrease in the DCPIP in a separate assay using succinate as a substrate and in the presence of rotenone to inhibit CI interference. The complex III (CIII) activity was evaluated (at 550 nm) by the increase in cytochrome *c* reduction absorbance using substrate DUbH_2_. The complex IV (CIV) activity was evaluated by cytochrome c oxidation (at 550 nm) absorbance using reduced cytochrome C as a substrate, and 1 mM sodium azide was used as a specific inhibitor of CIV. The ATP synthase activity was evaluated as previously described in a hexokinase-G6PDH-NADP^+^ reduction assay (at 340 nm) [47]. Synergy-Biotek microplate reader (Biotek Instruments, Winooski, VT, USA) was used for absorbance measurements at 37 °C. The activity of ATP synthase and CI to CIV was corrected by subtracting the activity determined in the presence of the corresponding specific inhibitor and expressed as nano mol per minute per milligram of protein (nmol/min/mg protein).

### 2.10. Krebs Cycle Metabolites and Enzyme Activity

Aconitase activity was evaluated in heart isolate mitochondria obtained immediately after rats were sacrificed. The aconitase activity was evaluated by determining the intermediate product cis-aconitate’s formation rate at 240 nm, as we previously described [15]. Citrate synthase activity was evaluated in freshly isolated mitochondria fraction determined by recording the increase in the absorbance at 412 nm of TNB [44]. Both activities were expressed as nmol/min/mg of protein. To determinate succinate and 2-oxoglutarate content, a portion of 0.03–0.1 g of the frozen tissue was powdered in a mortar under liquid N_2_. After, the tissue was mixed with 500 µL 3% perchloric acid/1 mM EDTA solution. The last suspension was neutralized with 3 M KOH/0.1 mM tris and centrifugated at 1800× *g* for 5 min at 4 °C. The supernatant was stored at −70 °C. For determination of 2-oxoglutarate dehydrogenase activity, 0.03–0.05 g of the frozen tissue was powdered and mixed with 500 µL SHE (220 mM sucrose 10 mM HEPES, 1 mM EGTA, pH 7.3) buffer plus 1 mM phenylmethanesulfonyl fluoride, 1 mM EDTA, 0.1% Triton X-100, and 5 mM DTT. The suspension was stored at −70 °C until use.

The 2-oxoglutarate dehydrogenase activity and succinate and 2-oxoglutarate levels were determined by spectrofluorometry with an excitation of 340 nm and an emission wavelength of 460 nm. First, succinate was determined at 25 °C in a buffer with 120 KCl, 20 mM M, 1 mM EGTA, and tris 20 mM, pH 8 plus 5 mM MgCl_2_, 0.15 mM NADH, 1 mM ATP, 2 mM PEP, 0.1 mM CoA, 2 PYK/2.8 U LDH, and 100 µL of neutralized supernatant previously stored at −70 °C. The reaction was started by adding 1 U succinyl CoA synthetase. Then, 2-oxoglutarate was determined at 30 °C in 10 mM HEPES/1 mM EGTA pH 7.4 plus 0.1 mM ADP, 0.1 mM NH_4_Cl, 0.15 mM NADH, and 100 µL of neutralized supernatant previously stored at −70 °C. The reaction was started by adding 5 U glutamate dehydrogenase. Finally, 2-oxoglutarate dehydrogenase activity was determined at 37 °C in 10 mM HEPES/1 mM EGTA pH 7.4. The assay contained 1 mM MgCl_2_, 0.5 mM NAD+, 5 mM 2-oxoglutarate, 0.5 mM thiamine pyrophosphate, 0.5 mM DTT, 0.02% Triton, 5 µM rotenone, and 30–50 mg of tissue extract. After 4 min of incubation, the reaction was started with 0.25 mM CoA. Activities were expressed as nmol/min/mg of protein.

### 2.11. Mitochondrial H_2_O_2_ Production and 4HNE Lipoperoxidation Levels

Mitochondrial H_2_O_2_ was measured as previously described [15,22] at 37 °C in a Synergy-Biotek microplate reader (Biotek Instruments, Winooski, VT, USA) using Amplex Red as a probe. Freshly isolated mitochondria were resuspended in MiR05 plus HRP 0.5 U/mL. Briefly, Amplex Red is oxidized in the presence of H_2_O_2_ to produce resorufin, whose fluorescence is detected at 530–590 nm. Complexes I or CII linked substrates were used to stimulate the respiratory states in each condition. In addition, a standard curve with different H_2_O_2_ concentrations was employed in each condition to ensure the linearity of the assay, and sequential additions were used to determine the production rate in each state. To eliminate the possible interference of other substrates with Amplex Red and verify the specificity concerning H_2_O_2_, negative controls were made with the addition of catalase (280 U/mL) and Gpx (280 U/mL plus GHS 10 µM) to the reaction medium. The activity was expressed as nmol/min/mg of protein. The lipoperoxidation marker 4-HNE levels were determined in heart homogenates by spectrophotometric using a standard curve of tetramethoxypropane as previously described [48]. Briefly, 1-methyl-2-phenylindole in acetonitrile: methanol (3:1) was added to homogenates, and the reaction was started with 37% HCl plus FeCl_3_. Synergy-Biotek microplate reader (Biotek Instruments, Winooski, VT, USA) was used for absorbance measurements at 586 nm after 1 h of incubation. Data were expressed as nmol of 4HNE per microgram of protein (nmol/μg protein).

### 2.12. Activity of Antioxidant Enzymes

As previously described, heart homogenates 100–200 mg were used to measure antioxidant enzyme activities [15]. Briefly, superoxide dismutase (SOD) activity was evaluated by spectrophotometry at 560 nm using NBT as a probe. Glutathione S-transferase (GST) activity was evaluated by measuring the increase in absorbance at 340 nm generated by the adduct GSH-CDNB. Glutathione reductase (GR) activity was evaluated by measuring the disappearance of NADPH at 340 nm. Glutathione peroxidase (GPx) activity was measured by the disappearance of NADPH at 340 nm in a coupled reaction with GR. The lipoperoxidation markers protein MDA and protein 4HNE adducts were evaluated by WB [44].

### 2.13. Mitochondrial Glutathione, GRX Activity, Mitochondrial Protein S-Glutathionylation Levels, and Cardiolipin Levels

Total glutathione (GSH plus GSSG) content was evaluated in fresh heart isolate mitochondria obtained immediately after rats were sacrificed. Total glutathione content and GSSG levels were measured in renal homogenates using a previously described method [15,49]. The total glutathione assay is based on the reaction of GSH with DTNB to produce GSSG-TNB adduct (GS-TNB), which is detectable at 412 nm. The GR added to the assay reduces the GS-TNB to GSH, which then reacts with DTNB. The rate of change in absorbance was compared with GSH standards. The measurement of GSSG was made similarly, but the samples were incubated with 2-VP, which scavenges GSH; this allows for GSSG to be reduced to GSH by GR. The levels of GSH were determined by subtraction of GSH content in 2-VP-treated samples to GSH content of samples without 2-VP.

Meanwhile, cardiolipin levels were determined in fresh mitochondria by commercial kits, including cardiolipin assay kit from Abcam (Cambridge, MA, USA). All measurements were normalized by protein content. The removal S-glutathionylation activity of the GRX was evaluated using a commercial kit from Cayman Chemical (Ann Arbor, MI, USA). In each case, the activities were normalized by protein content. The levels of total mitochondrial protein S-glutathionylation were evaluated by WB.

### 2.14. Electron Microscopy

The myocardiums were fixed, post-fixed in osmium tetroxide, dehydrated, and infiltrated in epoxy resin using the previously reported protocol [15]. The tissues were mounted on electron microscopy grids, and uranyl acetate and lead citrate salts were used to contrast. Grids were observed, and electron microscopy micrographs were taken with an electron microscope (Tecnai Spirit BioTwin, FEI, Hillsboro, OR, USA). The mitochondria with circular morphology and small ovoids were measured taking into account the diameter of each mitochondrion in its longest axis using the TEM Imaging & Analysis V 4.7 SP3 software (FEI Tecnology, Hillsboro, OR, USA) for quantification of the mitochondria smaller than 700 nm since it was the average size that resulted from the mitochondria count in the control group. All the counts were made in images with an area of 100 um^2^, and the counts of structures that indicate autophagy and mitophagy were made in images of the same area.

### 2.15. Statistics

Data are presented as mean ± standard error of the mean (SEM). They were analyzed by data analysis performed with Graph Pad Prism 8 (San Diego, CA, USA). They were analyzed by one-way analysis of variance with a subsequent Tukey test using the software Graph-Pad Prism 6 (San Diego, CA, USA). A *p*-value less than 0.05 was considered significant.

## 3. Results

### 3.1. NAC Prevented CRS Type III Triggers by FA-Induced AKI

**Figure 1:**
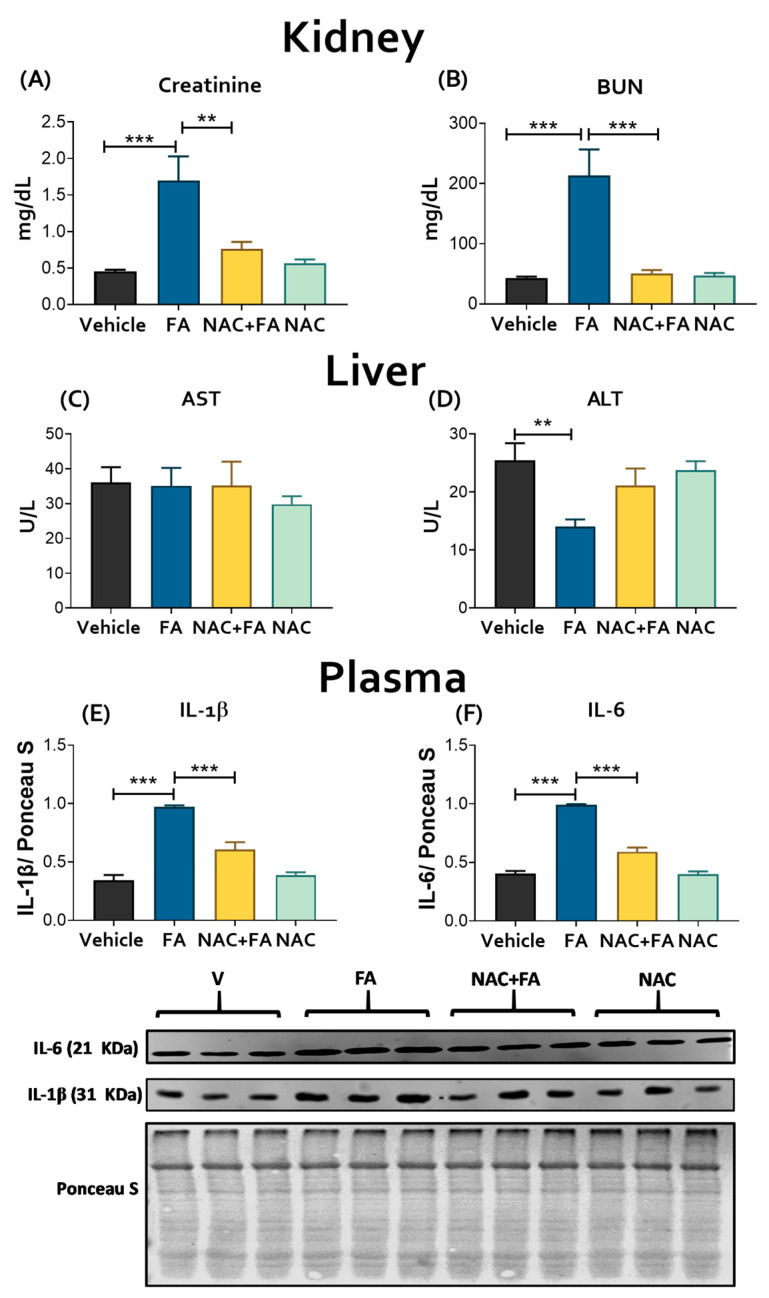
Evaluation of renal damage markers: (**A**) Creatinine and (**B**) blood urea nitrogen (BUN) evaluated in plasma; the analysis was conducted on day 7 of evolution (48 h after the last FA administration) using commercial kits. The results are expressed in milligrams per deciliter (mg/dL). Evaluation of liver damage markers: (**C**) aspartate aminotransferase (AST) and (**D**) alanine aminotransferase (ALT) in plasma; the analysis was conducted on day 7 of evolution using commercial kits, and the results are expressed in units of the corresponding assays per liter (U/L). Evaluation in plasma of proinflammatory cytokines by Western blot and its densitometry: (**E**) interleukin one beta (IL-1β) and (**F**) interleukin six (IL-6). Ponceau S Staining of the corresponding membranes was used as a control charger. Data are mean ± SEM, n = 5–6. ** *p* < 0.01, *** *p* < 0.001. FA = Folic Acid, NAC = N-acetyl-cysteine.

AKI development in the FA group was confirmed by the increase in serum creatinine (Figure 1A) and BUN (Figure 1B) compared to the vehicle. Because the liver and kidney are the principal storage organs of folate metabolites [50,51], we evaluated if FA also increased liver damage markers. However, as we showed in Figure 1C,D, there was no increase in the plasma enzyme activity of AST and ALT, suggesting no liver damage at least one week after the first FA administration. Interestingly, previous studies have shown that folic acid administration can decrease ALT and AST plasma levels in patients with liver damage [52,53]. These effects could be associated with the role of 5-methyltetrahydrofolate (folate active metabolite) in the synthesis of the S-adenosyl-L-methionine (SAM), the principal methyl donor that also regulates GSH synthesis [53,54,55], furthermore, low doses of folate have been also used to reduce hyperhomocysteinemia conditions by an increase in tetrahydrofolate [56,57]. Because homocysteine synthesis takes place mainly in the liver [55], reducing its levels by folic acid supplementation reduces liver injury and oxidative stress by reducing NOX superoxide production [58]. Therefore, the observed decrease in ALT plasma levels (Figure 1D) could be attributed to the regulation of SAM and homocysteine liver levels by the tetrahydrofolate; however, more studies are still necessary. Similarly, our results show higher levels of proinflammatory cytokines in plasma after FA administration (Figure 1E,F), which suggests the generation of a systemic proinflammatory environment in this group.

On the other hand, NAC pre-administration is effective in preventing AKI, reducing creatinine and BUN levels (Figure 1A,B), histological damage in the kidney (Supplementary Figure S1), and the increase in proinflammatory cytokines in plasma (Figure 1E,F), in agreement with our previous studies [15,22].

**Figure 2:**
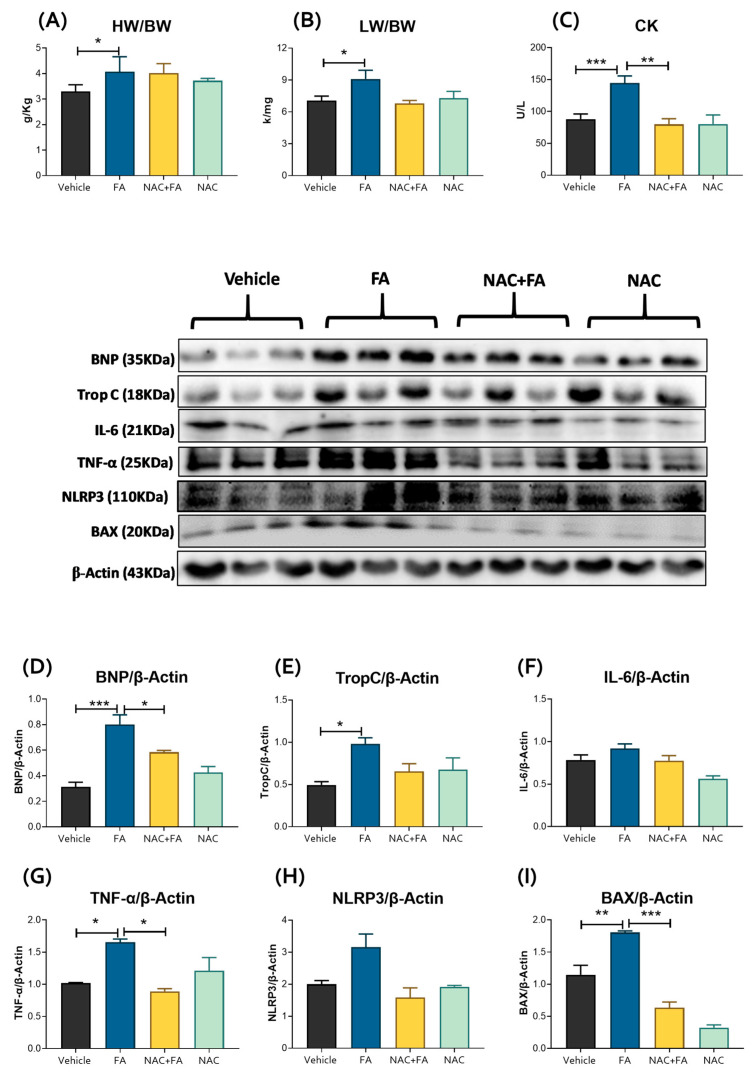
Heart damage markers. (**A**) HW/BW and (**B**) LW/BW ratios, (**C**) creatine kinase (CK) activity in the heart. Data are mean ± SEM, n = 5–6. Western blot representative images and their densitometries of proteins: (**D**) BNP, (**E**) Trop C, (**F**) IL-6, (**G**) TNF-α, (**H**) NRLP3 and (**I**) BAX in heart homogenates. β-Actin was used as loading controls. Data are mean ± SEM, n = 3. * *p* < 0.05, ** *p* < 0.01 and *** *p* < 0.001. BNP = brain natriuretic peptide, BW = body weight, HW = heart weight, LW = lung weight, Trop C = troponin C, IL-6 = Interleukin 6, TNF-α = tumor necrosis factor-alpha, NLRP3 = NLR family pyrin domain containing 3, BAX = Bcl-2-associated X, FA = Folic acid, NAC = N-acetyl-cysteine.

**Figure 3:**
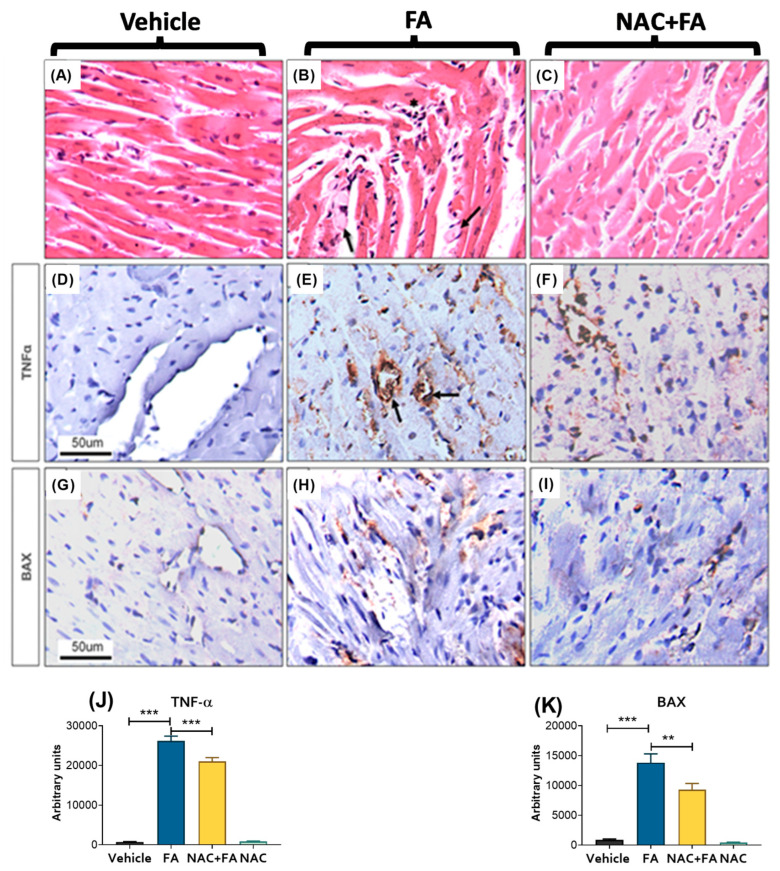
Representative micrographs of heart histology and TNF-alfa and BAX immunohistochemistry. (**A**) Normal heart histology of a rat from the control vehicle group. (**B**) FA-treated animal show groups of death cardiomyocytes with condensed nucleus and fragmented hyaline cytoplasm (***arrows***) and some interstitial lymphocytes (***white asterisk***). (**C**) NAC + FA treatment reduced tissue damage. (**D**) The control animal does not show TNF-alpha immunoreactivity. (**E**) In contrast, strong TNF-alpha immunoreactivity is seen in the cytoplasm of some myocardial and inflammatory cells; it is particularly intense in the endothelium of small blood vessels (***arrows***). (**F**) Rat treated with FA + NAC showed decreased TNF-alpha immunoreactivity; only the endothelium showed immunoreactivity. (**G**) The control rat does not show BAX immunoreactivity. (**H**) Numerous cardiomyocytes show BAX immunoreactivity in animals treated with FA. (**I**) Occasional myocytes show scare immunostaining to BAX in a rat from the NAC + FA group. Quantification of TNF-alpha (**J**) and BAX (**K**) immunoreactivity in the heart. The images used for quantification correspond to a size of area x field of 314,679.481 μm. Data are mean ± SEM, n = 7. ** *p* < 0.01, *** *p* < 0.001. BAX = Bcl-2 Associated X-protein, FA = Folic Acid, NAC = N-acetyl-cysteine, TNF-alpha = tumor necrosis factor alfa (All micrographs 400× magnification).

It has been well-documented that AKI can induce cardiac injury [10,11]. In this way, FA doses ≥ 250 mg/kg may induce cardiac inflammation and apoptosis and decrease systolic and diastolic left ventricular pressure, coronary flow, and heart rate evaluated in isolated rat hearts according to the Langendorff technique [38]. To confirm the CRS type III development in our model we evaluated heart weight, but no change was observed concerning the control group (Supplementary Table S1). However, the heart weight/body and lung weight/body weight ratios significantly increased concerning the control (Figure 2A,B). Additionally, the FA group showed higher levels of CK activity in plasma (Figure 2C), increased levels of BNP, troponin C, TNF-α, and cell death protein Bcl-2-associated (BAX) (Figure 2D,E,G,I), with no significant increase in IL-6 and NLR family pyrin domain containing 3 (NLRP3) heart levels (Figure 2F,H). Likewise, a histological analysis in the FA group by H&E staining showed focal groups of myocardiocytes with a condensed hyperchromatic nucleus and cytoplasmic fragmentation with hyalinization indicative of cell death compared to the control (Figure 3A,B). These cells were surrounded by chronic inflammatory infiltrate; there was also mild fibrosis in the walls of medium size blood vessels in the FA group (Figure 3B). The histology (Figure 3B) and TNF-alpha and BAX immunostaining evaluation (Figure 3E,H,J,K) in animals treated with FA verify the CRS type 3 development, characterized by heart inflammation and cell death induction. On the other hand, NAC pre-administration prevented the CK activity increase in plasma (Figure 2C) and the FA-induced increase in the heart damage markers BNP, TNF-α, and apoptotic protein BAX levels (Figure 2D,G,I). Furthermore, histological analysis shows occasional death or damaged cardiomyocytes and scare inflammatory cells (Figure 3C). NAC treatment also decreased inflammatory markers (Figure 2 and Figure 3). Immunohistochemistry in the FA group showed positive TNF-α immunostaining in inflammatory cells and some cardiomyocytes, and it was particularly intense in endothelial cells from blood vessels related to the inflammatory response (Figure 3E,J). There were also injured cardiomyocytes that showed immunoreactivity to BAX that denote apoptotic cells (Figure 3H,K). Indeed, NAC administration induced lesser TNF-α immunoreactivity; only endothelial cells showed positivity in some venules (Figure 3F,J), and occasional cardiomyocytes showed immunoreactivity to BAX (Figure 3I,K). Together, these results suggested that NAC partially prevented the inflammation and cell death triggered by CRS induced by FA.

**Figure 4:**
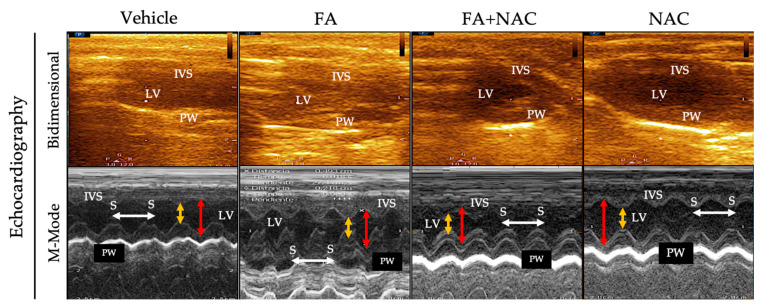
Representative images in 2D and echocardiography (Echo). (**Top image**): Representative two-dimensional echocardiographic images of the parasternal long-axis view in each group. (**Bottom image**): Corresponding M-mode at the mid-ventricular level from two-dimensional images. It is possible to measure the end-systolic (yellow double-headed arrow) and end-diastolic (red double-headed arrow) diameters of the left ventricle (LV), the thickness of the interventricular septum (IVS), and posterior wall (PW), as well as to calculate the heart rate using the distance between two consecutive systoles (S, white double-headed arrow). FA = folic acid; NAC = N-acetyl-cysteine.

Structural analysis and cardiac function by echocardiography showed that the lung weight (LW) normalized with the tibial length (TL) was lower in the NAC + FA group compared to the FA group (Supplementary Table S1) without favoring pulmonary congestion in rats. The structural parameters in the animals treated with NAC did not show significant changes. Likewise, the echocardiographic related to the interventricular septum (IVS) and the LV (LVPW) posterior wall did not present thickening of the ventricular wall or dilation of the cavities in any of the treatments (Figure 4 and Table 1). Although the LV dimensions in systole and diastole are smaller in the NAC + FA group, cardiac function alteration is not established since all animals’ ejection fraction (EF) was similar (Figure 4 and Table 1).

### 3.2. CRS Triggered by FA-Induced AKI Is Related to Mitochondrial Bioenergetics Alterations in the Heart

**Figure 5:**
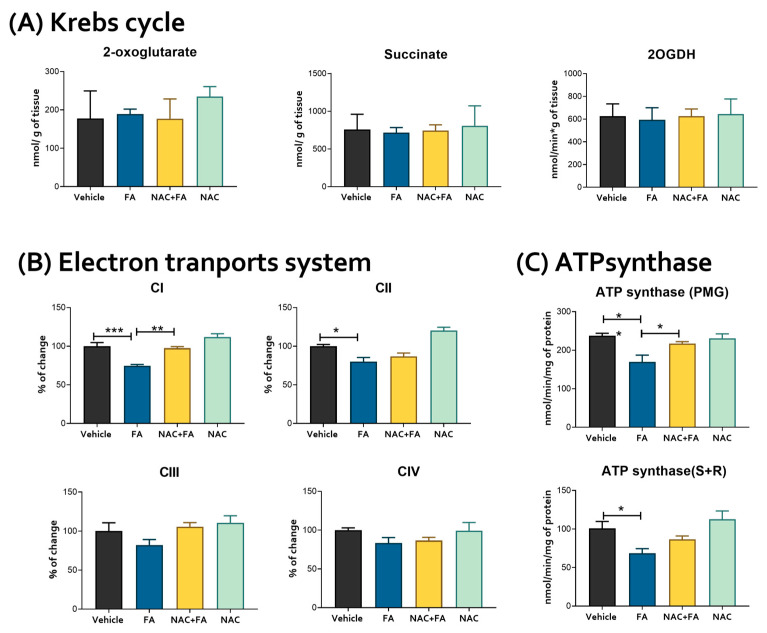
Heart mitochondrial bioenergetics. (**A**) Levels of the Krebs cycle intermediates 2-oxoglutarate and succinate and activity of 2-oxoglutarate dehydrogenase (2-OGDH) in heart homogenates. (**B**) The activity of respiratory complexes in isolated mitochondria from the heart: Complex I (CI), Complex II (CII), Complex III (CIII), Complex IV (CIV). (**C**) ATP synthase activity using PMG = pyruvate, malate, and glutamate and S + R = succinate plus rotenone as a substrate. Data are mean ± SEM, n = 5–6. * *p* < 0.05, ** *p* < 0.01, *** *p* < 0.001. FA = folic acid, NAC = N-acetyl-cysteine.

**Figure 6:**
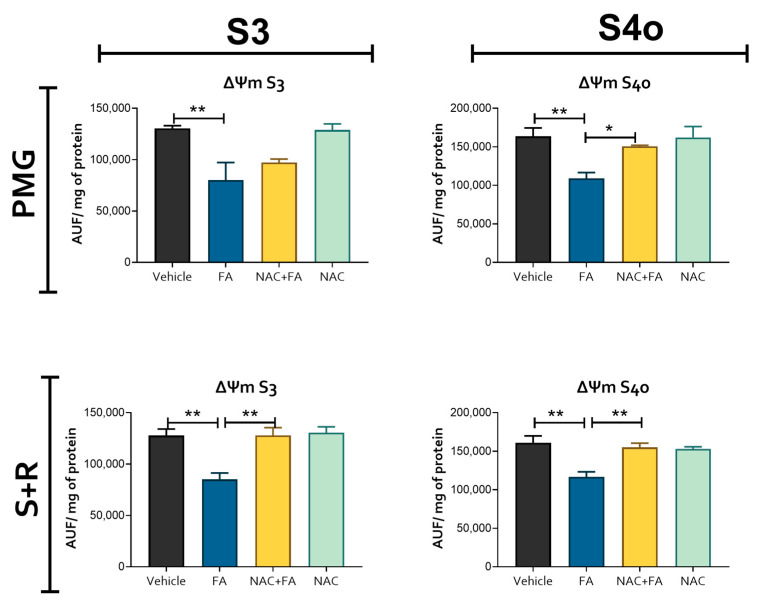
Heart mitochondrial membrane potential. Changes in mitochondrial membrane potential (ΔΨm) evaluated in isolated heart mitochondria using safranine O as a probe, AUF = arbitrary unit of florescence, PMG = pyruvate, malate, and glutamate, S + R = succinate + rotenone. S3 = state 3, S4o = state 4 induced by oligomycin. FA = folic acid, NAC = N-acetyl-cysteine. Data are mean ± SEM, n = 5–6. * *p* < 0.05, ** *p* < 0.01.

Heart metabolism is highly dependent on mitochondrial homeostasis [59], and alterations in this organelle can lead to cardiac damage [24,59]. Therefore, to elucidate if the observed structural damage triggered by FA-induced AKI administration was related to mitochondrial bioenergetics alteration, we evaluated the activity of the electron transport system (ETS) complexes and ATP synthase in heart-isolated mitochondria as well as the Krebs cycle enzymes and metabolites in heart homogenates. We did not observe significant changes in the Krebs cycle metabolites 2-oxoglutarate and succinate in the heart (Figure 5A) and kidney (Supplementary Figure S2) produced by FA. However, CI and CII activities presented a significant reduction in the FA group (Figure 5B), which, together with the ATP synthase activity reduction using both CI and CII linked substrates (Figure 5C), indicates a decrease in OXPHOS capacity in heart mitochondria after FA administration. This agrees with the reduction in ΔΨm observed in both S3 (a state in which ΔΨm is mainly used for ATP production) and S4o (a state in the absence of mitochondrial ATP production) in both CI and CII linked substrates (Figure 6), implying a permanent mitochondrial depolarization induced by FA. Together, our results suggest an early mitochondrial bioenergetics impairment in the heart induced by FA-AKI.

On the other hand, NAC pre-administration was able to prevent the reduction in mitochondrial CI activity (Figure 5B) and in ATP synthase activity when a CI-linked substrate (PMG) is used (Figure 5C) and partially prevented the ΔΨm membrane depolarization on both S3 and S4o induced by FA administration in the heart (Figure 6). Thus, these results indicate that the protective NAC effects on CRS would be related to its therapeutic effects in mitochondrial heart bioenergetics.

### 3.3. CRS Triggered by FA-AKI Is Related to Mitochondrial Oxidative Stress and GSH Reduction in the Heart

**Figure 7:**
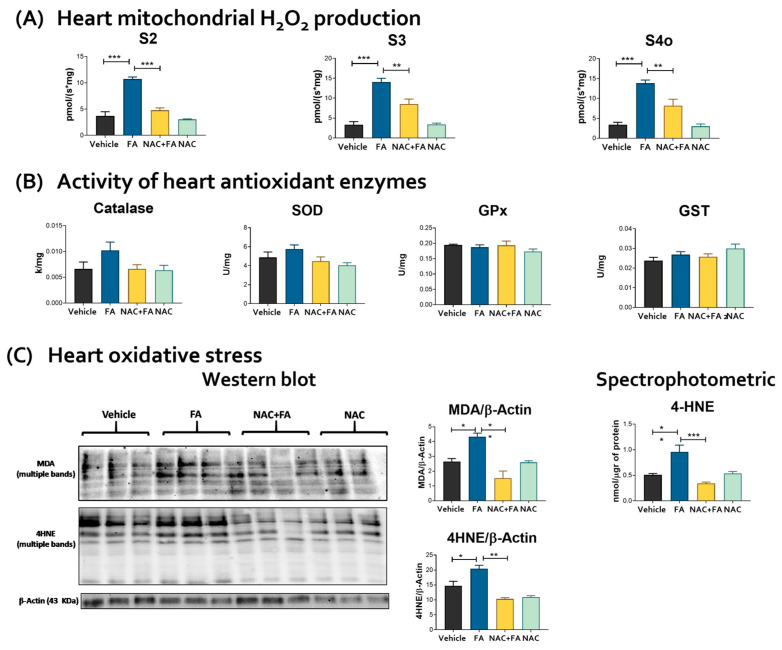
**Oxidative stress.** (**A**) Rate of mitochondrial hydrogen peroxide (H_2_O_2_) production. S2 = State 2, S3 = State 3, S4 = State 4. (**B**) Activity of antioxidant enzymes in heart homogenates: catalase, SOD = superoxide dismutase, GPx = glutathione peroxidase, and GST = glutathione S-transferase. Data are mean ± SEM, n = 5–6. (**C**) MDA and 4HNE lipoperoxidation markers evaluated by Western blot (mean ± SEM, n = 3) and by spectrophotometric technique (mean ± SEM, n = 5–6) in heart homogenates. MDA = malondialdehyde, 4HNE = 4-hydroxynonenal. β-Actin was used as a control charger. FA = folic acid, NAC = N-acetyl-cysteine. * *p* < 0.05, ** *p* < 0.01, *** *p* < 0.001.

**Figure 8:**
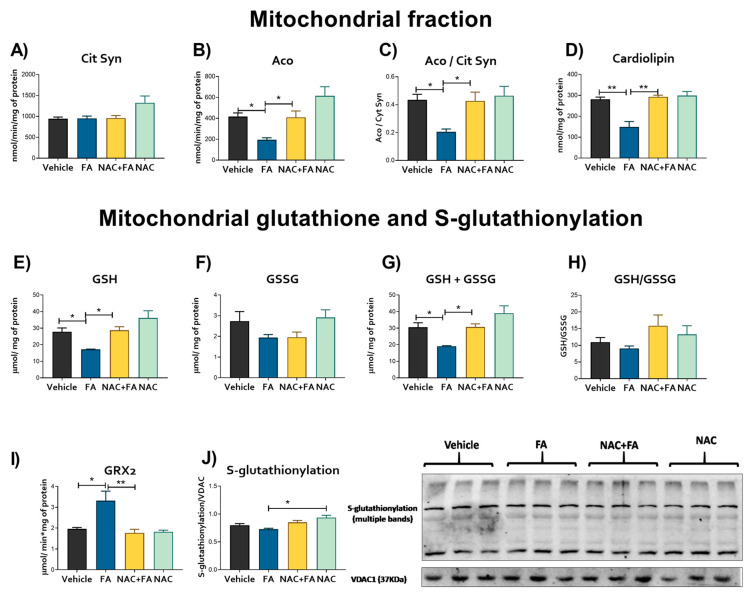
Mitochondrial stress markers. Activity of (**A**) citrate synthase (Cit Syn), (**B**) aconitase (Aco), (**C**) Aco/Cit Syn ratio, and (**D**) cardiolipin levels in isolated heart mitochondria. Levels of (**E**) GSH = glutathione, (**F**) glutathione disulfide (GSSG), (**G**) GSH plus GSSG, (**H**) GSH/GSSG ratio, (**I**) removal S-glutathionylation activity of the glutaredoxin 2 (GRX) and (**J**) total S-glutathionylation levels evaluated by Western blot and their densitometry in isolated mitochondria fraction. FA = folic acid, NAC = N-acetyl-cysteine. Data are mean ± SEM, n = 5–6. * *p* < 0.05 and ** *p* < 0.01.

Mitochondrial bioenergetics alterations may induce the redox to unbalance, inducing higher ROS production by different mitochondrial enzymes [20,60,61]. Therefore, to elucidate if bioenergetics alteration affects the mitochondrial redox state in the heart, we evaluated the rate of mitochondrial H_2_O_2_ production and oxidative stress markers in the heart. The specificity of the assay for the determination of the rate of H_2_O_2_ production was verified using scavenger by catalase and GPx (Supplementary Figure S3). The heart mitochondria of the FA group showed an enormously increased rate of H_2_O_2_ production for the vehicle/control group, regardless of the type of respiratory substrate (Figure 7A). Although, we did not observe changes in the antioxidant enzymes activities in the heart, except a slight tendency to increase in catalase activity in the FA group (Figure 7B). Lipoperoxidation markers MDA and 4HNE in hearts evaluated both by Western blot and quantitatively by spectrophotometry showed higher levels in the FA group (Figure 7C), confirming oxidative stress in this organ. Furthermore, mitochondrial heart aconitase activity and the aconitase/citrate synthase activities ratio were reduced in the FA group without changes in mitochondrial citrate synthase activity, confirming heart mitochondrial oxidative stress (Figure 8A–C). Likewise, mitochondrial cardiolipin levels were reduced in the FA group (Figure 8D), consistently with the increase in oxidative stress, favoring the reduction in mitochondrial GSH and GSH + GSSH (Figure 8E–G) without a significant decrease in GSSG levels and GSH/GSSG (Figure 8F–H). Summarizing, the data indicate that the higher mitochondrial ROS production induced by FA-AKI triggers oxidative stress. It was recently proposed that S-glutathionylation, a post-translational and redox-sensitive modification involving disulfide between GSH and Cys residue, is a mechanism that links mitochondrial bioenergetics with the redox state and ROS production in this organelle [1,2,3,4,5]. Because the protein S-glutathionylation highly depends on GSH in the mitochondrial matrix [1], we speculate that the FA-induced increase induced a decrease in the mitochondrial GSH levels which would activate the S-glutathionylation removal activity of GRX2. As we showed in Figure 8I, the removal of the S-glutathionylation activity of the GRX2 was increased in the FA group. However, no significant changes were observed in the total glutathionylation levels (Figure 8J).

In contrast, the NAC + FA group presented lower rates of mitochondrial H_2_O_2_ production than the FA group (Figure 7A), preventing an increase in lipoperoxidation in the heart (Figure 7C), mitochondrial cardiolipin depletion (Figure 8D), and reduction in aconitase and the rate aconitase/citrate synthase activities (Figure 8C,D) by preventing alterations of the heart mitochondrial glutathione levels (Figure 8E–G). Interestingly, NAC administration prevented an increase in the removal of the S-glutathionylation activity of the GRX2 (Figure 8I), which is congruent with the restoration in GSH and GSH/GSSG ratio (Figure 8E–H) observed in this group. We speculate that the changes in the S-glutathionylation of specific proteins, such as mitochondrial CI (the principal target for protein S-glutathionylation in heart mitochondria [1,8]), would be related to the NAC protective mechanism; however, deeper proteomics studies would be necessary to elucidate the molecular mechanism. Summarizing, our results displayed that NAC protection of the mitochondrial redox state by GSH regulation is also active in cardiac tissue.

### 3.4. CRS Triggers by FA-AKI Induce Mitochondrial Fission in the Heart

**Figure 9:**
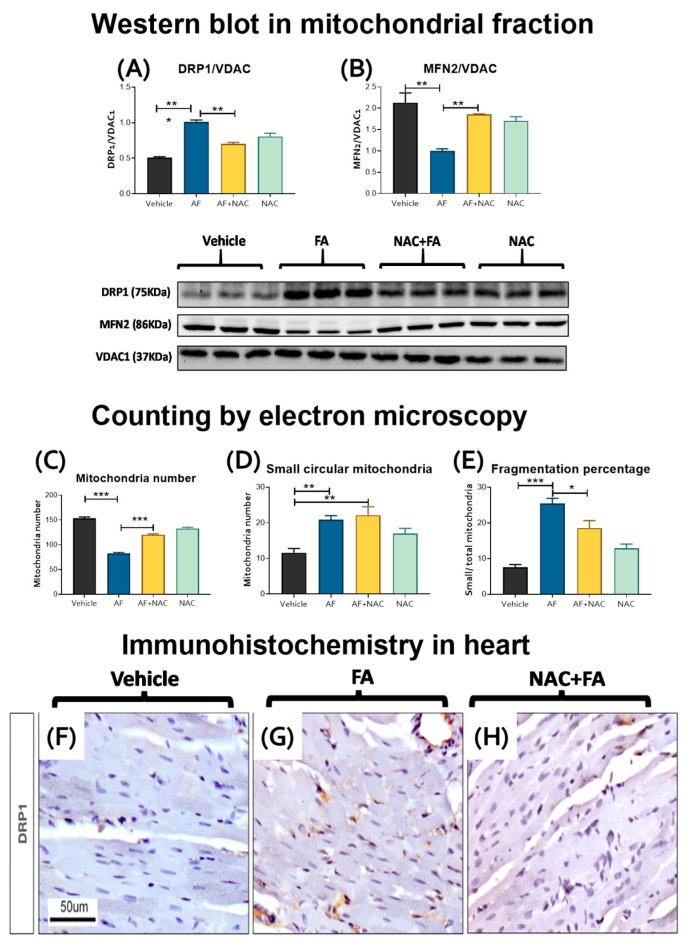
Mitochondrial dynamics. Western proteins in the heart isolated mitochondrial and their densitometries of fission protein (**A**) DRP1 and (**B**) fusion proteins MFN2. VDAC1 was used as a loading control. Data are mean ± SEM, n = 3. ** *p* < 0.01. Counting by electron microscopy: (**C**) total Mitochondrial number and (**D**) small mitochondria with ovoid or circular morphology per image in cardiomyocyte, as well as (**E**) fragmentation rate (small/total mitochondria). The electron microscopy images used for counting correspond to a size field of 100 μm. Data are mean ± SEM, n = 7. * *p* < 0.05, ** *p* < 0.01, *** *p* < 0.001. DRP1 immunohistochemistry in heart: (**F**) Control animal shows scarce DRP1 immunoreactivity. (**G**) Higher DRP1 immunoreactivity is seen in the cytoplasm of some myocardial cells of the FA group. (**H**) Rat treated with FA + NAC showed a decrease in DRP1 immunoreactivity in cardiac cells. DRP1 = dynamin-related protein 1, VDAC1 = voltage-dependent anion selective channel 1, MFN2 = mitofusin 2. FA = folic acid, NAC = N-acetyl-cysteine.

**Figure 10:**
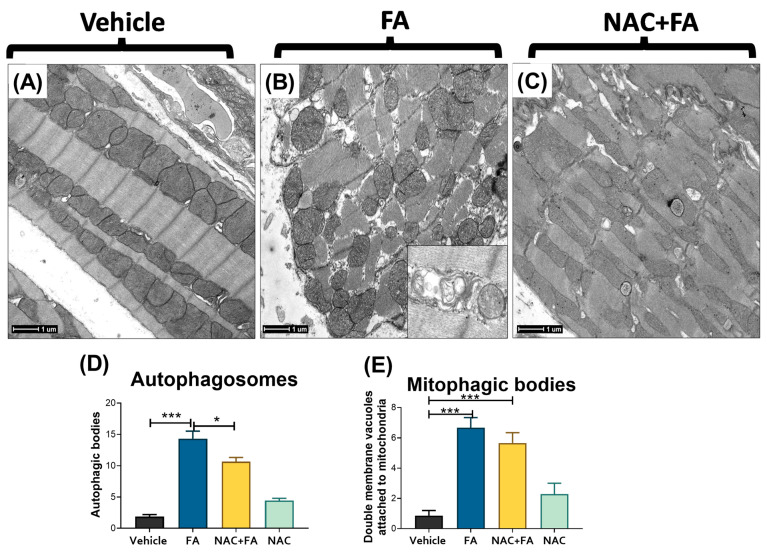
Representative electron microscopy micrographs of the different experimental groups. (**A**) Normal ultrastructural morphology of myocardial cell from control vehicle rat. (**B**) Myocardial cells that show myofilaments disarray and numerous round (fission) mitochondria from FA-treated animals, the inset show double membrane cytoplasmic vacuoles that correspond to autophagosomes. (**C**) Myocardial cell rat from FA plus NAC animal show long (fusion) mitochondria. Counting of: (**D**) number of autophagosomes and (**E**) double membrane vacuoles attached to mitochondria (mitophagic) bodies per image; the electron microscopy images used for counting correspond to a size field of 100 μm. Data are mean ± SEM, n = 7. * *p* < 0.05, *** *p* < 0.001. FA = folic acid, NAC = N-acetyl-cysteine.

Mitochondrial bioenergetics impairment and oxidative stress may induce an imbalance in mitochondrial dynamics, favoring the mitochondrial network fragmentation (fission) over the joining of two or more mitochondria (fusion) [18,62,63]. Thus, to evaluate the changes in mitochondrial dynamics, we determined the levels of fission proteins DRP1 and FIS1 and fusion proteins MFN1, MFN2, and OPA1 in the heart mitochondria. The FA-treated group showed increased levels of the fission protein DRP1 (Figure 9A) without changes in FIS1 levels (Supplementary Figure S4). We observed a reduction in the MFN2 levels (Figure 9B). However, we did not observe significant changes in the levels of the other fusion proteins nor the mitochondrial protease LONP1 (Supplementary Figure S4). Interestingly, the electron microscopy evaluation showed a decrease in the mitochondrial number per cardiomyocytes in the FA group (Figure 9C and Figure 10B), an increase in the number of small, circular mitochondria per cardiomyocyte (Figure 9D), and in the ratio of small/total mitochondria (Figure 9E), thus suggesting a higher degree of mitochondrial fragmentation. These results were confirmed by immunohistochemistry, where the hearts from the FA group showed greater DRP1 immunostaining with respect to the vehicle (Figure 9F,G). The NAC + AF treated group showed a significant reversion of dynamic protein changes (Figure 9A,B), mitochondrial number (Figure 9C), and fragmentation percentage (Figure 9E), as well as less DRP1 immunostaining in cardiomyocytes (Figure 9H).

In comparison with the control group that showed cardiomyocytes with normal distribution and morphology of mitochondria and myofilaments array (Figure 10A), the cardiomyocytes of the FA group showed a higher number of small mitochondria with ovoid or circular morphology that corresponded to fission mitochondria (Figure 9D,E and Figure 10). In this FA group, many cardiomyocytes also showed a disarray of myofilaments and a higher number of autophagosomes (Figure 10B,D), as well as double membrane vacuoles attached to the mitochondria that correspond to mitophagy bodies (Figure 10B,E). The FA plus NAC group showed myocardial cells with long mitochondria characteristic of fusion mitochondria (Figure 10C), a lower number of small mitochondria (Figure 9D), and autophagosomes (Figure 10D). Summarizing, our results imply a slight shift of mitochondrial dynamics in the fission process in the hearts of FA-treated animals. On the other hand, NAC pretreatment prevented the FA-induced increase in DRP1 mitochondria levels (Figure 9A,G), increased MFN2 levels (Figure 9B), and preserved mitochondria structure (Figure 9C–E and Figure 10C).

### 3.5. NAC Prevented Mitochondrial Biogenesis Reduction in the Heart Induced by FA-AKI

**Figure 11:**
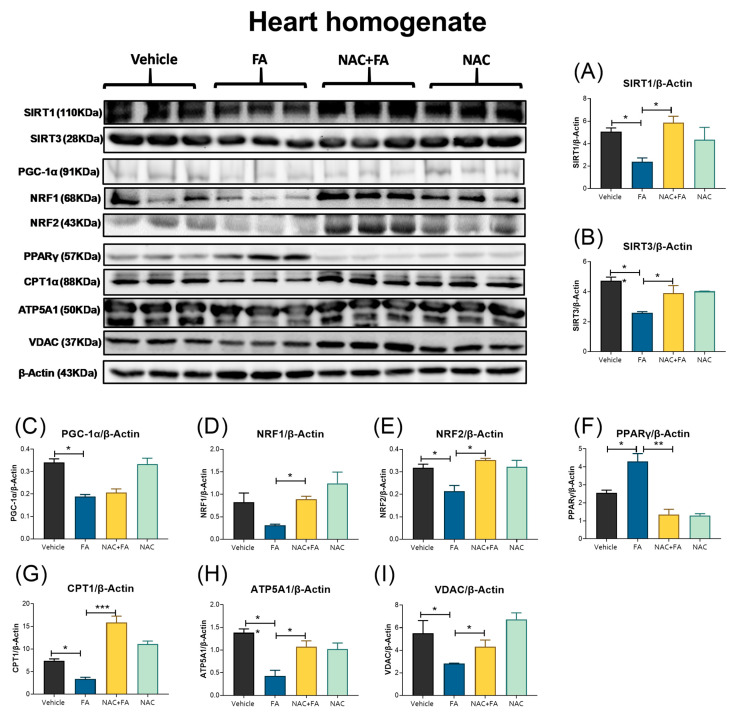
Mitochondrial biogenesis and mass markers. Western blot representative images and their densitometries of mitochondrial biogenesis protein: (**A**) SIRT1, (**B**) SIRT3, (**C**) PGC-1α, (**D**) NRF1, (**E**) NRF2, and (**F**) PPARγ. As well as mitochondrial proteins: (**G**) CPT1α, (**H**) ATP5A1, and (**I**) VDAC in heart homogenates. SIRT1 = sirtuin 1, SIRT3 = sirtuin 3, PGC-1α = peroxisome proliferator-activated receptor-gamma coactivator, NRF1 = nuclear respiratory factor 1, NRF2 = nuclear respiratory factor 2, PPARγ = peroxisome proliferator-activated receptor gamma, CPT1 = carnitine palmitoyltransferase 1A, ATP5A1 = ATP synthase subunit 5 A. Voltage-dependent anion channel (VDAC) and β-Actin were used as loading controls. FA = folic acid, NAC = N-acetyl-cysteine. Data are mean ± SEM, n = 3. * *p* < 0.05, ** *p* < 0.01, *** *p* < 0.001.

Mitochondrial bioenergetic impairment and oxidative stress may induce the imbalance in mitochondrial dynamics. It has been reported that an FA overdose causes a decrease in renal mitochondrial biogenesis [15,64,65]. Therefore, we evaluated mitochondrial biogenesis protein in the heart and mitochondria mass markers. We found that FA induces a decrease in the levels of SIRT1 and SIRT3 (Figure 11A,B). Likewise, PGC-1α and NRF-2 biogenesis protein levels in the heart are reduced in the FA group (Figure 11C–E) and increased PPARγ levels (Figure 11F). Consistently, the mitochondrial proteins CPT1, ATP5A, and VDAC (Figure 11H–J) also decreased in the hearts from the FA groups, suggesting a reduction in mitochondrial mass and number (Figure 9C). On the other hand, the NAC + FA group preserved the SIRT1 and SIRT3 levels (Figure 11A,B) and increased the NRF1 and NRF-2, reducing PPARγ protein levels with respect to the FA group (Figure 11D,E,G). NAC also increases mitochondrial proteins CPT1, ATP5A, and VDAC in the heart (Figure 11H–J), suggesting both mitochondrial biogenesis and mass preservation.

### 3.6. NAC Prevented Mitophagy Induction in Heart Triggers by FA-AKI

**Figure 12:**
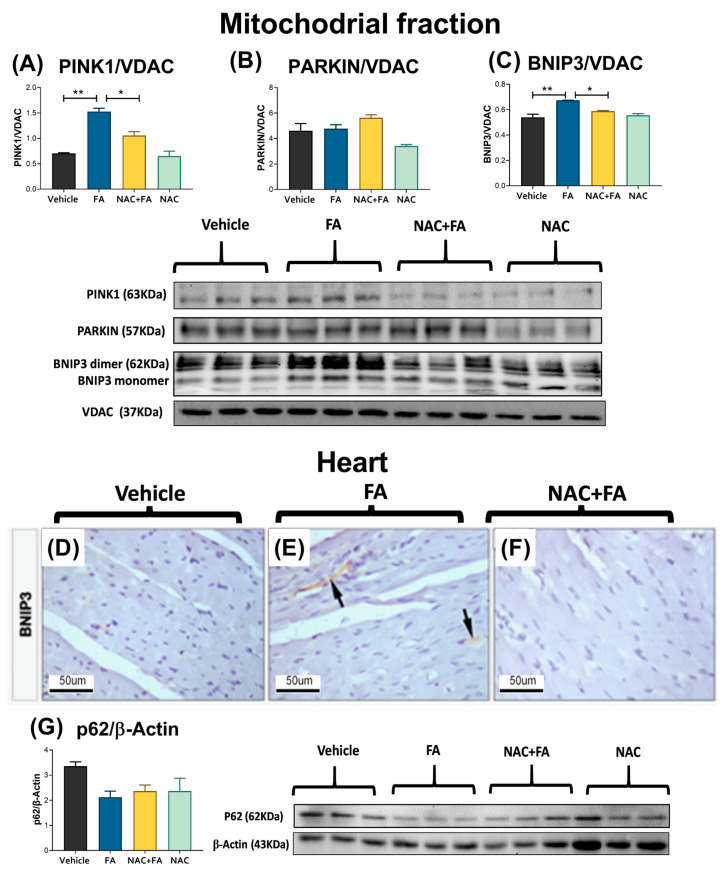
Mitophagy and autophagy markers. Western blot representative images and their densitometries of mitophagy: (**A**) PINK1, (**B**) PARKIN, and (**C**) BNIP3 in heart-isolated mitochondria. Counting corresponds to a size field of 100 μm. Data are mean ± SEM, n = 7. * *p* < 0.05, ** *p* < 0.01. **BNIP3 immunohistochemistry in heart:** (**D**) Control animal does not show BNIP3 immunoreactivity. (**E**) BNIP3 immunoreactivity can be seen in some myocardial cells of the FA group (black arrows). (**F**) FA + NAC rats do not show BNIP3 immunoreactivity in cardiac cells. (**G**) The p62 autophagy protein in heart homogenates. VDAC1 and β-Actin were used as a loading control. PINK1 = PTEN induced kinase 1, BNIP3 = BCL2 interacting protein 3, VDAC = voltage-dependent anion channel 1. FA = folic acid, NAC = N-acetyl-cysteine. Data are mean ± SEM, n = 3. * *p* < 0.05 and ** *p* < 0.01.

Impaired mitophagy flux favors the accumulation of renal-damaged mitochondria in FA-induced AKI and CKD [15,44]. Therefore, we evaluated mitophagy-related proteins in the heart to characterize alterations in the mitophagy flux. We observed an increase in the FA group in the mitochondrial levels of PINK1 and BNIP3 mitophagy protein levels (Figure 12A,C), without changes in the parkin and p62 levels in the heart (Figure 12B,G). Likewise, the FA group showed higher BNIP3 immunoreactivity in the heart in comparison to the vehicle (Figure 12D,E), suggesting the mitophagy induction. Interestingly, NAC pretreatment reduces the increase in both PINK1 and BNIP3 mitophagy levels (Figure 12A,C) in the heart mitochondria and did not show BNIP3 immunoreactivity in the heart (Figure 12F), suggesting the prevention of mitophagy induction.

## 4. Discussion

The mitochondrial role in the molecular pathways connecting renal and cardiac damage is not fully understood [12,13]. In this way, the FA-AKI model can be used to study these molecular mechanisms [22,32,38,65]. After intraperitoneal administration, folate is transported by the reduced folate carrier exchanger (RFCE) or by the high-affinity folate receptor (HFR) inside the cells [50,51,66,67] and stored as impermeable polyglutamate derivatives [51,66]. The kidney is susceptible to accumulating FA metabolites because of its high expression of HFR [50], thus in high doses (≥100 mg/kg), FA saturates the HFR and RFCE, which, together with the low FA solubility, triggers renal FA precipitation [64,65,68,69]. Moreover, intracellular folate reduction by the dihydrofolate reductase (DHFR) [67,70] depletes the NADPH equivalents, favoring renal oxidative stress and inflammation [15,36,69]. This organelle is more vulnerable to damage because 40% of total folates are stored in mitochondria [50,51]. Indeed, renal mitochondrial dysfunction is a crucial mechanism in the pathogenesis of FA-AKI [15,22,64,65] and remains even in CKD [71,72,73,74]. We verified the FA-AKI development (Figure 1) in agreement with the cell death and inflammation in mitochondria-rich nephron segments [15,36,75]. Interestingly, damage markers were increased in the liver (Figure 1C,D), the second major folate reservoir in the body [70,76]. These results imply that the specificity of FA damage cannot be explained only by high HFR and RFCE levels in the kidney and liver [70,76], suggesting that high mitochondria abundance would be decisive for tissue damage development.

In AKI, cell death and inflammation favor the release of several cardio-renal connectors, resulting in cardiac injury [10,11]. In particular, proinflammatory factors trigger cardiac myocyte apoptosis and oxidative stress in the heart [10,77,78]. This agrees with our results that showed higher plasma levels of IL-1β and IL-6 in the FA groups (Figure 1D–F), and previous reports showed an early increase in the plasma levels of proinflammatory cytokines like IL-1β, IL-5, IL-6, IL-18, interferon-γ, TNF-α, MCP-1, and fibrosis growth factor 23 (FGF23) [34,35,36,37] can act on distal organs like the heart, suggesting that they could act as cardio-renal connectors that increase cell death markers in the heart, favoring CRS type 3 development (Figure 2 and Figure 3). Although the echocardiography function evaluation did not show significant changes at this time (Table 1 and Figure 4), our results are in agreement with previous reports in which FA (250 mg/kg) induced inflammation and cardiomyocyte apoptosis, followed by a posterior decrease in functional parameters like systolic and diastolic left ventricular pressure, coronary flow, and heart rate [38], suggesting that heart inflammation and apoptosis are early triggers of subsequent cardiac function loss. This phenomenon was also observed in models like nephrectomy and renal ischemia/reperfusion-induced CRS [7,12,13,79].

Recent evidence suggests mitochondria damage modulates cell death and inflammation [24,59]. In AKI, tubular cell death promotes the bloodstream release of the danger-associated molecular patterns (DAMPs); between them, mitochondrial-derived DAMPs (mtDAMPs) are potent immunological enhancers by activation of receptor-like stimulators of interferon genes (STING), toll-like receptor 9 (TLR9), and/or NLRP3 inflammasome [11,80]. Previous studies showed that FA-AKI increases serum proinflammatory cytokines IL-1β, IL-5, IL-6, IL-18, interferon-γ, TNF-α, MCP-1, and fibrosis growth factor 23 (FGF23) [34,35,36,37] that may trigger inflammation in the heart. Consistently, our results in the FA-AKI group showed higher levels of IL-6 and TNF-α and a tendency to increase NLRP3 inflammasome in the heart (Figure 2). It was proposed that these cardio-renal connectors interact with a receptor on the surface of cardiomyocytes inducing mitochondria bioenergetic changes [59]. In CRS type 3 induced by renal ischemia-reperfusion, the increase in bloodstream IL-6 depletes myocardial ATP levels and ΔΨm [9,12]. In agreement, our results showed a reduction in cardiac mitochondrial EST complexes and OXPHOS activity (Figure 5B,C) and lower ΔΨm (Figure 6). Interestingly, we previously reported that the same FA dose causes kidney mitochondrial decoupling and ΔΨm, OXPHOS capacity, and CI activity reduction, even in the absence of occlusion phenomena [15], suggesting that in mitochondria-rich tissue, high doses of FA per se reduces OXPHOS. Since in the heart ATP production is mainly maintained by the OXPHOS [24,59,81], the mitochondrial bioenergetic alterations compromise the cardiomyocyte function [59,82]. Furthermore, in AKI and CRS, the loss of ΔΨm triggers cell death induction [7,12,83].

In FA-AKI, CI dysfunction increases renal mitochondrial ROS [15,22]. In the heart, our results also showed that CI reduction (Figure 5B) is also accompanied by higher cardiac mitochondrial ROS production (Figure 7A). Although the antioxidant enzyme activities were unchanged (Figure 7B), in contrast with as reported in the kidney [15,22], FA also induced oxidative stress in the cardiac mitochondria (Figure 7A and Figure 8A–C), generating a decrease in mitochondrial cardiolipin levels. We previously showed that FA induces kidney mitochondrial oxidative stress by reducing renal mitochondria GSH equivalents [15,22]. Consistently, our data showed that FA also leads to a cardiac mitochondrial reduction in GSH and GSH + GSSG (Figure 8E,G), indicating that a GSH unbalance is involved in the mitochondrial prooxidant state in the heart.

Mitochondrial GSH links energy metabolism with the redox state by S-glutathionylation, a critical mechanism that regulates the activity and formation of mitochondrial complexes [42,84]. We previously demonstrated that FA induces the loss of mitochondrial GSH and S-glutathionylation in the kidney. Meanwhile, NAC’s S-glutathionylation induction prevented renal mitochondrial damage [15]. Remarkably, our results showed similar protection in mitochondrial glutathione levels by NAC in the heart after CRS type 3 (Figure 8E,G), avoiding an increase in lipoperoxidation (Figure 7C), mitochondrial cardiolipin depletion (Figure 8D), and a reduction in aconitase/citrate synthase activities (Figure 8C,D). Furthermore, mitochondrial GSH preservation by NAC protects CI activity (Figure 5B), avoiding ΔΨm depolarization (Figure 6) to maintain the ATP synthase activity in the heart (Figure 5C). Our results, together with those previously described in the kidney [15], suggest that the effect of NAC would be associated with the GSH levels and therefore the regulation of S-glutathionylation in cardiac mitochondria. However, more is still necessary to confirm this hypothesis. Similarly, NAC administration preserved mitochondrial oxygen consumption, coupling, ATP synthase activity, and supercomplexes assembly in post-ischemic hearts, reducing infarct size and cardiac function loss [85]. Consistently, our results showed that NAC heart mitochondrial bioenergetics preservation (Figure 5 and Figure 6) is related to lower inflammation, fibrosis, and cell death in the heart (Figure 2 and Figure 3). Thus, suggesting that cardiac bioenergetic preservation by NAC in the AKI event can prevent CRS development.

We previously demonstrated FA-induced kidney mitochondrial fragmentation by ΔΨm loss and oxidative stress [15]. Our result showed similar behavior in cardiac tissue from the FA group, where DRP1 mitochondrial levels increase (Figure 9), and myocardial cells show numerous fragmented and round mitochondria (Figure 10). This cardiac tendency to fission has also been reported in CRS-type 3 induced by renal ischemia-reperfusion, which triggers cardiac apoptosis [7,25]. Additionally, a renal mitochondrial biogenesis decrease is central in AKI pathology [63,65,86]. Previous reports showed that FA-AKI decreases the mRNA levels of ETS proteins and the mtDNA number [64,65], associated with a PGC-1α and NRF2 protein decrease in the proximal tubule [15,22]. Here we showed for the first time in the heart that FA decreased PGC-1α and NRF-2 protein levels (Figure 11C–E) by downregulating SIRT1 and SIRT3 (Figure 11A,B). This mitochondrial biogenesis reduction favors the downregulating mitochondrial proteins CPT1, ATP5A, and VDAC (Figure 11G–I), contributing to the heart bioenergetics crisis. Consistently, previous studies showed that oxidative stress induces PGC-1α reduction in CRS [87,88]. At the same time, in cardiomyocytes, SIRT1 pharmacological inhibition triggers a PGC-1α decrease and an ROS production increase, favoring fibrosis and inflammation [89], suggesting a pathological loop between heart SIRTs, oxidative stress, and inflammation. In this way, the administration of TWEAK, a nuclear factor kappa-light-chain-enhancer of activated B cells (NF-κB) inhibitor, preserved the PGC-1α levels in the renal and improved renal function in mice administered with FA [65]. This agrees with our results, in which NAC preserved heart SIRT1 and SIRT3 levels (Figure 11A,B), increasing NRF1 and NRF2 (Figure 11D,E), and CPT1, ATP5A and VDAC levels (Figure 10G–I). These data suggest that NAC protects mitochondrial biogenesis and mass, and ultrastructure in the heart (Figure 9, Figure 10 and Figure 11) by mitochondrial GSH preservation (Figure 8E,G).

Finally, we previously demonstrated that FA induced an impaired mitophagy flux in the kidney, favoring the accumulation of damaged mitochondria [15]. Our results showed an increase in PINK1 and BNIP3 in heart mitochondria (Figure 12A,C), and parkin and p62 levels in the heart did not substantially change (Figure 12B,G). However, the autophagosome number increase in myocardial cells observed by the electron microscopy (Figure 10) suggested an impaired mitophagy flux. Although more studies using autophagy inhibitors are still necessary to elucidate the state of autophagy flux in this model, the restoration of levels by NAC (Figure 10 and Figure 12) suggests that oxidative stress and GSH depletion could be directly involved in these alterations.

**Figure 13:**
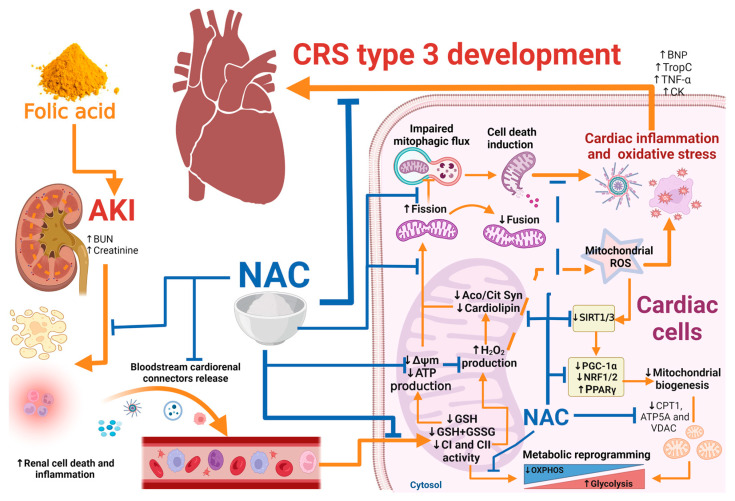
Integrative scheme. After its administration, FA induces AKI, favoring inflammatory and cell death processes in kidney segments like the proximal tubule. This triggers the release to the bloodstream of cardio-renal connectors, which can reach the heart cells. Thus, cardio-renal connector interaction with cardiac cells induces mitochondrial impairment by GSH and GSH + GSSH depletion in this organelle and CI and CII activity reduction, triggering a drop in ATP production and ΔΨm. Additionally, the complexes alteration in the mitochondrial heart triggers a H_2_O_2_ production increase, resulting in mitochondrial oxidative stress and a reduction in cardiolipin levels, which together with ΔΨm loss induce mitochondrial impairment in mitophagy flux, favoring mitochondrial damage accumulation and cell death induction. Likewise, mitochondrial ROS reduce SIRT1/3 levels inducing a reduction in mitochondrial biogenesis factors and decreasing mitochondrial proteins CPT1, ATP5A, and VDAC. Together, these alterations favor the energetic metabolic reprogramming in the heart. On the other hand, mitochondrial bioenergetic alterations and oxidative stress enhance the heart’s proinflammatory processes and cell death, ultimately leading to the CRS type 3 development in this model. In contrast, NAC pre-administration prevents inflammatory and cell death processes in kidney and cardio-renal connectors increase. NAC also prevents heart mitochondrial bioenergetics alliterations, preserving ATP and ΔΨm by CI activity and glutathione balance preservation, thus preventing the mitochondrial dynamics shift to fission and decreasing biogenesis and mitochondrial mass in the heart. By mitochondrial preservation, NAC administration prevented inflammatory and cell death markers in the heart and cardiac oxidative stress, thus preventing CRS type 3 development in FA-AKI. Aco = aconitase; AKI = acute kidney injury; ATP = adenosine triphosphate; ATP5A1 = ATP synthase subunit 5 A; BNP = brain natriuretic peptide; BUN = blood urea nitrogen; CI = Complex I; CII = Complex II; Cit Syn = citrate Synthase; CPT1 = carnitine palmitoyltransferase 1A, CK = creatine kinase; CRS = cardio-renal syndrome; FA = folic acid; GSH = reduced glutathione; GSSG = glutathione disulfide; H_2_O_2_ = hydrogen peroxide; NAC = N-acetyl-cysteine; NRF1 = nuclear respiratory factor 1; NRF2 = nuclear respiratory factor 2; OXPHOS = oxidative phosphorylation; PGC-1α = peroxisome proliferator-activated receptor-gamma coactivator; PPARγ = peroxisome proliferator-activated receptor gamma; ROS = reactive oxygen species; SIRT1 = sirtuin 1; SIRT3 = sirtuin 3; TNF-α = tumor necrosis factor-alpha; Trop C = troponin C; VDAC = voltage-dependent anion channel; ΔΨm = mitochondrial membrane potential.

Summarizing, as we showed in Figure 13, we proposed that FA-AKI triggers a bloodstream increase in cardio-renal connectors as a result of renal parenchyma cell death and inflammation. The cardio-renal connectors interact with cardiac cells, inducing energetic metabolic reprogramming characterized by mitochondrial ATP production reduction and ΔΨm loss. The mitochondrial heart alterations result from GSH depletion and CI and CII activity reduction, which triggers H_2_O_2_ production and oxidative stress that induces mitochondrial fragmentation, biogenesis inhibition, and impairment in mitophagy flux. Mitochondrial bioenergetic alterations and oxidative stress enhance heart inflammation and cell death, leading to CRS development. In contrast, NAC prevented CRS type 3 development by heart mitochondrial bioenergetics and redox state preservation, preventing an imbalance in mitochondrial dynamics and decreases in the biogenesis and mitochondrial mass, as well as heart inflammation. These effects could be associated with CI protection by glutathione balance preservation in this organelle. Although more studies are still needed to elucidate the role of S-glutathionylation in CRS, our data clearly show that mitochondrial bioenergetic alterations play a fundamental role in the mechanism that allows damage progression from the kidney to the heart.

## 5. Conclusions

The early induction of the heart mitochondrial OXPHOS reduction, triggered by oxidative stress, glutathione depletion, mitochondrial biogenesis, and mass reduction by FA-AKI, plays a crucial role in heart inflammation and CRS type 3 development. Furthermore, preserving the NAC of heart mitochondrial bioenergetics and biogenesis during an AKI event can be a valuable strategy to prevent CRS development.

## Figures and Tables

**Table 1:** Heart parameters obtained from echocardiography analysis. IVS = interventricular septum, LVDd = left ventricle (LV) dimension at end-diastole; LVDs = LV dimension at end-systole; LVPW = LV dimension posterior wall; EF = ejection fraction; FS = fractional shortening; HR = heart rate; bpm = beats per minute. Data are mean ± SEM, n = 5–6. * *p* < 0.05 vs Vehicle. FA = folic acid, NAC = N-acetyl-cysteine.

Parameter	Vehicle	FA	NAC+FA	NAC
IVS (mm)	2 ± 0.19	2.02 ± 0.25	2.0 ± 0.15	1.9 ± 0.19
LVDd (mm)	5.3 ± 0.33	5.1 ± 0.38	4.6 ± 0.31 *	5.1 ± 0.45
LVDs (mm)	2.6± 0.23	2.5± 0.5	2.04 ± 0.11 *	2.2± 0.14
LVPW (mm)	1.9 ± 0.14	1.8 ± 0.17	1.8 ± 0.13	1.8 ± 0.08
EF(%)	83.8 ± 6.16	90.4 ± 1.19	83.3 ± 1.5	90.9 ± 1.52
FS (%)	49.7 ± 3.2	51.3 ± 9.2	55.7 ± 1.9	56.7 ± 2.5
HR (bpm)	369.7 ± 67.9	424.3 ± 58.9	351.6 ± 45.8	356.2 ± 22.8

## Data Availability

Data are contained within the article.

## Supplementary Materials

The following supporting information can be downloaded at: https://www.mdpi.com/article/10.3390/antiox12081592/s1.

## References

[1] Hoste E.A.J., Kellum J.A., Selby N.M., Zarbock A., Palevsky P.M., Bagshaw S.M., Goldstein S.L., Cerdá J., Chawla L.S. (2018). Global Epidemiology and Outcomes of Acute Kidney Injury. Nat. Rev. Nephrol..

[2] Ronco C., Bellomo R., Kellum J.A. (2019). Acute Kidney Injury. Lancet.

[3] Kellum J.A., Romagnani P., Ashuntantang G., Ronco C., Zarbock A., Anders H.J. (2021). Acute Kidney Injury. Nat. Rev. Dis. Prim..

[4] Fogo A.B., Cohen A.H., Colvin R.B., Jennette J.C., Alpers C.E. (2014). Fundamentals of Renal Pathology.

[5] Shafi T., Coresh J., Himmelfarb J., Sayegh M.H. (2010). Chronic Kidney Disease: Definition, Epidemiology, Cost, and Outcomes. Chronic Kidney Disease, Dialysis, and Transplantation.

[6] Li P.K.T., Burdmann E.A., Mehta R.L. (2013). Acute Kidney Injury: Global Health Alert. Hong Kong J. Nephrol..

[7] Sumida M., Doi K., Ogasawara E., Yamashita T., Hamasaki Y., Kariya T., Takimoto E., Yahagi N., Nangaku M., Noiri E. (2015). Regulation of Mitochondrial Dynamics by Dynamin- Related Protein-1 in Acute Cardiorenal Syndrome. J. Am. Soc. Nephrol..

[8] Rangaswami J., Bhalla V., Blair J.E.A., Chang T.I., Costa S., Lentine K.L., Lerma E.V., Mezue K., Molitch M., Mullens W. (2019). Cardiorenal Syndrome: Classification, Pathophysiology, Diagnosis, and Treatment Strategies: A Scientific Statement From the American Heart Association. Circulation.

[9] Doi K., Noiri E. (2016). Mitochondrial Dysfunction in Cardiorenal Syndrome. Antioxid. Redox Signal..

[10] Chuasuwan A., Kellum J.A. (2012). Cardio-Renal Syndrome Type 3: Epidemiology, Pathophysiology, and Treatment. Semin. Nephrol..

[11] Kingma J.G., Simard D., Rouleau J.R., Drolet B., Simard C. (2017). The Physiopathology of Cardiorenal Syndrome: A Review of the Potential Contributions of Inflammation. J. Cardiovasc. Dev. Dis..

[12] Wang J., Sun X., Wang X., Cui S., Liu R., Liu J., Fu B., Gong M., Wang C., Shi Y. (2021). Grb2 Induces Cardiorenal Syndrome Type 3: Roles of IL-6, Cardiomyocyte Bioenergetics, and Akt/MTOR Pathway. Front. Cell Dev. Biol..

[13] Bigelman E., Cohen L., Aharon-Hananel G., Levy R., Rozenbaum Z., Saada A., Keren G., Entin-Meer M. (2018). Pathological Presentation of Cardiac Mitochondria in a Rat Model for Chronic Kidney Disease. PLoS ONE.

[14] Szeto H.H., Liu S., Soong Y., Seshan S.V., Cohen-Gould L., Manichev V., Feldman L.C., Gustafsson T. (2017). Mitochondria Protection after Acute Ischemia Prevents Prolonged Upregulation of IL-1 *β* and IL-18 and Arrests CKD. J. Am. Soc. Nephrol..

[15] Aparicio-Trejo O.E., Reyes-Fermín L.M., Briones-Herrera A., Tapia E., León-Contreras J.C., Hernández-Pando R., Sánchez-Lozada L.G., Pedraza-Chaverri J. (2019). Protective Effects of N-Acetyl-Cysteine in Mitochondria Bioenergetics, Oxidative Stress, Dynamics and S-Glutathionylation Alterations in Acute Kidney Damage Induced by Folic Acid. Free Radic. Biol. Med..

[16] Reyes-Fermín L.M., Avila-Rojas S.H., Aparicio-Trejo O.E., Tapia E., Rivero I., Pedraza-Chaverri J. (2019). The Protective Effect of Alpha-Mangostin against Cisplatin-Induced Cell Death in LLC-PK1 Cells Is Associated to Mitochondrial Function Preservation. Antioxidants.

[17] Avila-Rojas S.H., Tapia E., Briones-Herrera A., Aparicio-Trejo O.E., León-Contreras J.C., Hernández-Pando R., Pedraza-Chaverri J. (2018). Curcumin Prevents Potassium Dichromate (K2Cr2O7)-Induced Renal Hypoxia. Food Chem. Toxicol..

[18] Molina-Jijón E., Aparicio-Trejo O.E., Rodríguez-Muñoz R., León-Contreras J.C., del Carmen Cárdenas-Aguayo M., Medina-Campos O.N., Tapia E., Sánchez-Lozada L.G., Hernández-Pando R., Reyes J.L. (2016). The Nephroprotection Exerted by Curcumin in Maleate-Induced Renal Damage Is Associated with Decreased Mitochondrial Fission and Autophagy. BioFactors.

[19] Forbes J.M., Thorburn D.R. (2018). Mitochondrial Dysfunction in Diabetic Kidney Disease. Nat. Rev. Nephrol..

[20] Aparicio-Trejo O.E., Tapia E., Sánchez-Lozada L.G., Pedraza-Chaverri J. (2018). Mitochondrial Bioenergetics, Redox State, Dynamics and Turnover Alterations in Renal Mass Reduction Models of Chronic Kidney Diseases and Their Possible Implications in the Progression of This Illness. Pharmacol. Res..

[21] Aparicio-Trejo O.E., Aranda-Rivera A.K., Osorio-Alonso H., Martínez-Klimova E., Sánchez-Lozada L.G., Pedraza-Chaverri J., Tapia E. (2022). Extracellular Vesicles in Redox Signaling and Metabolic Regulation in Chronic Kidney Disease. Antioxidants.

[22] Aparicio-Trejo O.E., Avila-Rojas S.H., Tapia E., Rojas-Morales P., León-Contreras J.C., Martínez-Klimova E., Hernández-Pando R., Sánchez- Lozada L.G., Pedraza-Chaverri J. (2020). Chronic Impairment of Mitochondrial Bioenergetics and β-Oxidation Promotes Experimental AKI-to-CKD Transition Induced by Folic Acid. Free Radic. Biol. Med..

[23] Martínez-Klimova E., Aparicio-Trejo O.E., Gómez-Sierra T., Jiménez-Uribe A.P., Bellido B., Pedraza-Chaverri J. (2020). Mitochondrial Dysfunction and Endoplasmic Reticulum Stress in the Promotion of Fibrosis in Obstructive Nephropathy Induced by Unilateral Ureteral Obstruction. BioFactors.

[24] Keshavarz-Bahaghighat H., Darwesh A.M., Sosnowski D.K., Seubert J.M. (2020). Mitochondrial Dysfunction and Inflammaging in Heart Failure: Novel Roles of CYP-Derived Epoxylipids. Cells.

[25] Doi K. (2016). Kidney-Heart Interactions in Acute Kidney Injury. Nephron.

[26] Brenner B.M., Troy J.L., Daugharty T. (1971). Dynamics of Glomerular Ultrafiltration in the Rat. J. Clin. Investig..

[27] Jia G., Aroor A.R., Sowers J.R. (2014). Estrogen and Mitochondria Function in Cardiorenal Metabolic Syndrome.

[28] Correa F., Buelna-Chontal M., Hernández-Reséndiz S., García-Niño W.R., Roldán F.J., Soto V., Silva-Palacios A., Amador A., Pedraza-Chaverrí J., Tapia E. (2013). Curcumin Maintains Cardiac and Mitochondrial Function in Chronic Kidney Disease. Free Radic. Biol. Med..

[29] Tamaki M., Miyashita K., Wakino S., Mitsuishi M., Hayashi K., Itoh H. (2014). Chronic Kidney Disease Reduces Muscle Mitochondria and Exercise Endurance and Its Exacerbation by Dietary Protein through Inactivation of Pyruvate Dehydrogenase. Kidney Int..

[30] Ortiz A., Sanchez-Niño M.D., Izquierdo M.C., Martin-Cleary C., Garcia-Bermejo L., Moreno J.A., Ruiz-Ortega M., Draibe J., Cruzado J.M., Garcia-Gonzalez M.A. (2015). Translational Value of Animal Models of Kidney Failure. Eur. J. Pharmacol..

[31] Chunsun D., Lawrence P.K., Youhua L. (2008). Animal Models of Kidney Diseases. Sourcebook of Models for Biomedical Research.

[32] Szczypka M.S., Westover A.J., Clouthier S.G., Ferrara J.L.M., Humes H.D. (2005). Rare Incorporation of Bone Marrow-Derived Cells into Kidney after Folic Acid-Induced Injury. Stem Cells.

[33] He S., Liu N., Bayliss G., Zhuang S. (2013). EGFR Activity Is Required for Renal Tubular Cell Dedifferentiation and Proliferation in a Murine Model of Folic Acid-Induced Acute Kidney Injury. AJP Ren. Physiol..

[34] Li X., Zou Y., Fu Y., Xing J., Wang K., Wan P., Wang M. (2021). Ibudilast Attenuates Folic Acid—Induced Acute Kidney Injury by Blocking Pyroptosis Through TLR4-Mediated NF- κ B and MAPK Signaling Pathways. Front. Pharmacol..

[35] Durlacher-betzer K., Hassan A., Levi R., Axelrod J., Silver J., Naveh-many T. (2018). Interleukin-6 Contributes to the Increase in Fibroblast Growth Factor 23 Expression in Acute And&nbsp;Chronic Kidney Disease. Kidney Int..

[36] Martin-Sanchez D., Ruiz-Andres O., Poveda J., Carrasco S., Cannata-Ortiz P., Sanchez-Niño M.D., Ruiz Ortega M., Egido J., Linkermann A., Ortiz A. (2017). Ferroptosis, but Not Necroptosis, Is Important in Nephrotoxic Folic Acid–Induced AKI. J. Am. Soc. Nephrol..

[37] Kim S.M., Kim Y.G., Kim D.J., Park S.H., Jeong K.H., Lee Y.H., Lim S.J., Lee S.H., Moon J.Y. (2018). Inflammasome-Independent Role of NLRP3 Mediates Mitochondrial Regulation in Renal Injury. Front. Immunol..

[38] Nikolic T., Petrovic D., Matic S., Turnic T.N., Jeremic J., Radonjic K., Srejovic I., Zivkovic V., Bolevich S., Bolevich S. (2020). The Influence of Folic Acid-Induced Acute Kidney Injury on Cardiac Function and Redox Status in Rats. Naunyn-Schmiedeberg’s Arch. Pharmacol..

[39] Sharma M., Sud A., Kaur T., Tandon C., Singla S.K. (2016). N-Acetylcysteine with Apocynin Prevents Hyperoxaluria-Induced Mitochondrial Protein Perturbations in Nephrolithiasis. Free Radic. Res..

[40] Amini A., Masoumi-Moghaddam S., Morris D.L. (2016). Utility of Bromelain and N-Acetylcysteine in Treatment of Peritoneal Dissemination of Gastrointestinal Mucin-Producing Malignancies.

[41] Tamma G., Valenti G. (2016). Evaluating the Oxidative Stress in Renal Diseases: What Is the Role for S-Glutathionylation?. Antioxid. Redox Signal..

[42] Mailloux R.J., Treberg J.R. (2016). Protein S-Glutathionlyation Links Energy Metabolism to Redox Signaling in Mitochondria. Redox Biol..

[43] Shen X.L., Zhang Y., Xu W., Liang R., Zheng J., Luo Y., Wang Y., Huang K. (2013). An ITRAQ-Based Mitoproteomics Approach for Profiling the Nephrotoxicity Mechanisms of Ochratoxin A in HEK 293 Cells. J. Proteomics.

[44] Aparicio-Trejo O.E., Rojas-Morales P., Avila-Rojas S.H., León-Contreras J.C., Hernández-Pando R., Jiménez-Uribe A.P., Prieto-Carrasco R., Sánchez-Lozada L.G., Pedraza-Chaverri J., Tapia E. (2020). Temporal Alterations in Mitochondrial β-Oxidation and Oxidative Stress Aggravate Chronic Kidney Disease Development in 5/6 Nephrectomy Induced Renal Damage. Int. J. Mol. Sci..

[45] Prieto-Carrasco R., Silva-Palacios A., Rojas-Morales P., Aparicio-Trejo O.E., Medina-Reyes E.I., Hernández-Cruz E.Y., Sánchez-Garibay C., Salinas-Lara C., Pavón N., Roldán F.J. (2021). Unilateral Ureteral Obstruction for 28 Days in Rats Is Not Associated with Changes in Cardiac Function or Alterations in Mitochondrial Function. Biology.

[46] Hernández-Reséndiz S., Correa F., García-Niño W.R., Buelna-Chontal M., Roldán F.J., Ramírez-Camacho I., Delgado-Toral C., Carbó R., Pedraza-Chaverrí J., Tapia E. (2015). Cardioprotection by Curcumin Post-Treatment in Rats with Established Chronic Kidney Disease. Cardiovasc. Drugs Ther..

[47] Aparicio-Trejo O.E., Tapia E., Molina-Jijón E., Medina-Campos O.N., Macías-Ruvalcaba N.A., León-Contreras J.C., Hernández-Pando R., García-Arroyo F.E., Cristóbal M., Sánchez-Lozada L.G. (2017). Curcumin Prevents Mitochondrial Dynamics Disturbances in Early 5/6 Nephrectomy: Relation to Oxidative Stress and Mitochondrial Bioenergetics. BioFactors.

[48] García-Arroyo F.E., Gonzaga-Sánchez G., Tapia E., Muñoz-Jiménez I., Manterola-Romero L., Osorio-Alonso H., Arellano-Buendía A.S., Pedraza-Chaverri J., Roncal-Jiménez C.A., Lanaspa M.A. (2021). Osthol Ameliorates Kidney Damage and Metabolic Syndrome Induced by a High-Fat/High-Sugar Diet. Int. J. Mol. Sci..

[49] Negrette-Guzmán M., García-Niño W.R., Tapia E., Zazueta C., Huerta-Yepez S., León-Contreras J.C., Hernández-Pando R., Aparicio-Trejo O.E., Madero M., Pedraza-Chaverri J. (2015). Curcumin Attenuates Gentamicin-Induced Kidney Mitochondrial Alterations: Possible Role of a Mitochondrial Biogenesis Mechanism. Evid. Based Complement. Altern. Med..

[50] Gaudeuille A., N’Demanga Kamoune J., Ngakoula-Mbangui D., Mamadou N.N. (2002). Profile of Surgical Emergencies in Rural Areas: The Regional Hospital of Bambari in Central Africa as an Example. Dakar Médical.

[51] Fox J.T., Stover P.J. (2008). Folate-Mediated One-Carbon Metabolism. Vitam. Horm..

[52] Welzel T.M., Katki H.A., Sakoda L.C., Evans A.A., London W.T., Chen G., O’Broin S., Shen F.M., Lin W.Y., McGlynn K.A. (2007). Blood Folate Levels and Risk of Liver Damage and Hepatocellular Carcinoma in a Prospective High-Risk Cohort. Cancer Epidemiol. Biomarkers Prev..

[53] Medici V., Virata M.C., Peerson J.M., Stabler S.P., French S.W., Gregory J.F., Albanese A., Bowlus C.L., Devaraj S., Panacek E.A. (2011). S-Adenosyl-L-Methionine Treatment for Alcoholic Liver Disease: A Double-Blinded, Randomized, Placebo-Controlled Trial. Alcohol. Clin. Exp. Res..

[54] Anstee Q.M., Day C.P. (2012). S-Adenosylmethionine (SAMe) Therapy in Liver Disease: A Review of Current Evidence and Clinical Utility. J. Hepatol..

[55] Medici V., Halsted C.H. (2013). Folate, Alcohol, and Liver Disease. Mol. Nutr. Food Res..

[56] Hwang S.-Y., Siow Y.L., Au-Yeung K.K.W., House J., Karmin O. (2011). Folic Acid Supplementation Inhibits NADPH Oxidase-Mediated Superoxide Anion Production in the Kidney. Am. J. Physiol. Ren. Physiol..

[57] Wyatt C.M., Spence J.D. (2016). Folic Acid Supplementation and Chronic Kidney Disease Progression. Kidney Int..

[58] Woo C.W.H., Prathapasinghe G.A., Siow Y.L., Karmin O. (2006). Hyperhomocysteinemia Induces Liver Injury in Rat: Protective Effect of Folic Acid Supplementation. Biochim. Biophys. Acta Mol. Basis Dis..

[59] Shi S., Zhang B., Li Y., Xu X., Lv J., Jia Q., Chai R., Xue W., Li Y., Wang Y. (2022). Mitochondrial Dysfunction: An Emerging Link in the Pathophysiology of Cardiorenal Syndrome. Front. Cardiovasc. Med..

[60] Shadel G.S., Horvath T.L. (2015). Mitochondrial ROS Signaling in Organismal Homeostasis. Cell.

[61] Quinlan C.L., Gerencser A.A., Treberg J.R., Brand M.D. (2011). The Mechanism of Superoxide Production by the Antimycin-Inhibited Mitochondrial Q-Cycle. J. Biol. Chem..

[62] Zhan M., Brooks C., Liu F., Sun L., Dong Z. (2013). Mitochondrial Dynamics: Regulatory Mechanisms and Emerging Role in Renal Pathophysiology.

[63] Bhargava P., Schnellmann R.G. (2017). Mitochondrial Energetics in the Kidney. Nat. Rev. Nephrol..

[64] Stallons L.J., Whitaker R.M., Schnellmann R.G. (2014). Suppressed Mitochondrial Biogenesis in Folic Acid-Induced Acute Kidney Injury and Early Fibrosis. Toxicol. Lett..

[65] Ruiz-Andres O., Suarez-Alvarez B., Sánchez-Ramos C., Monsalve M., Sanchez-Niño M.D., Ruiz-Ortega M., Egido J., Ortiz A., Sanz A.B. (2016). The Inflammatory Cytokine TWEAK Decreases PGC-1α Expression and Mitochondrial Function in Acute Kidney Injury. Kidney Int..

[66] Chancy C.D., Kekuda R., Huang W., Prasad P.D., Kuhnel J.M., Sirotnak F.M., Roon P., Ganapathy V., Smith S.B. (2000). Expression and Differential Polarization of the Reduced-Folate Transporter-1 and the Folate Receptor α in Mammalian Retinal Pigment Epithelium. J. Biol. Chem..

[67] Nazki F.H., Sameer A.S., Ganaie B.A. (2014). Folate: Metabolism, Genes, Polymorphisms and the Associated Diseases. Gene.

[68] Huguenin M.E., Birbaumer A., Brunner F.P., Thorhorst J., Schmidt U., Dubach U.C., Thiel G. (1978). An Evaluation of the Role of Tubular Obstruction in Folic Acid-Induced Acute Renal Failure in the Rat. Nephron.

[69] Gupta A., Puri V., Sharma R., Puri S. (2012). Folic Acid Induces Acute Renal Failure (ARF) by Enhancing Renal Prooxidant State. Exp. Toxicol. Pathol..

[70] Ducker G.S., Rabinowitz J.D. (2017). One-Carbon Metabolism in Health and Disease. Cell Metab..

[71] Ortiz A., Lorz C., Catalan M.P., Danoff T.M., Yamasaki Y., Egido J., Neilson E.G. (2000). Expression of Apoptosis Regulatory Proteins in Tubular Epithelium Stressed in Culture or Following Acute Renal Failure. Kidney Int..

[72] Dai C., Yang J., Liu Y. (2002). Single Injection of Naked Plasmid Encoding Hepatocyte Growth Factor Prevents Cell Death and Ameliorates Acute Renal Failure in Mice. J. Am. Soc. Nephrol..

[73] Doi K., Okamoto K., Negishi K., Suzuki Y., Nakao A., Fujita T., Toda A., Yokomizo T., Kita Y., Kihara Y. (2006). Attenuation of Folic Acid-Induced Renal Inflammatory Injury in Platelet-Activating Factor Receptor-Deficient Mice. Am. J. Pathol..

[74] Ortega A., Rámila D., Izquierdo A., González L., Barat A., Gazapo R., Bosch R.J., Esbrit P. (2005). Role of the Renin-Angiotensin System on the Parathyroid Hormone-Related Protein Overexpression Induced by Nephrotoxic Acute Renal Failure in the Rat. J. Am. Soc. Nephrol..

[75] Fang T.-C. (2005). Proliferation of Bone Marrow-Derived Cells Contributes to Regeneration after Folic Acid-Induced Acute Tubular Injury. J. Am. Soc. Nephrol..

[76] Lucock M. (2000). Folic Acid: Nutritional Biochemistry, Molecular Biology, and Role in Disease Processes. Mol. Genet. Metab..

[77] Edelstein C.L., Akcay A., Nguyen Q. (2009). Mediators of Inflammation in Acute Kidney Injury. Mediators Inflamm..

[78] De Deyn P.P., Vanholder R., D’Hooge R. (2003). Nitric Oxide in Uremia: Effects of Several Potentially Toxic Guanidino Compounds. Kidney Int. Suppl..

[79] Jeremy BS M.P. (2012). Renal Failure Is Associated with Driving of Gene Expression towards Cardiac Hypertrophy and Reduced Mitochondrial Activity. J. Clin. Exp. Cardiolog..

[80] Zhang Q., Raoof M., Chen Y., Sumi Y., Sursal T., Junger W., Brohi K., Itagaki K., Hauser C.J. (2010). Circulating Mitochondrial DAMPs Cause Inflammatory Responses to Injury. Nature.

[81] Ishimoto Y., Inagi R. (2016). Mitochondria: A Therapeutic Target in Acute Kidney Injury. Nephrol. Dial. Transplant..

[82] Che R., Yuan Y., Huang S., Zhang A. (2014). Mitochondrial Dysfunction in the Pathophysiology of Renal Diseases. Am. J. Physiol. Physiol..

[83] Plotnikov E.Y., Kazachenko A.V., Vyssokikh M.Y., Vasileva A.K., Tcvirkun D.V., Isaev N.K., Kirpatovsky V.I., Zorov D.B. (2007). The Role of Mitochondria in Oxidative and Nitrosative Stress during Ischemia/Reperfusion in the Rat Kidney. Kidney Int..

[84] Mailloux R.J. (2020). Protein S-Glutathionylation Reactions as a Global Inhibitor of Cell Metabolism for the Desensitization of Hydrogen Peroxide Signals. Redox Biol..

[85] Ramírez-Camacho I., Correa F., El Hafidi M., Silva-Palacios A., Ostolga-Chavarría M., Esparza-Perusquía M., Olvera-Sánchez S., Flores-Herrera O., Zazueta C. (2018). Cardioprotective Strategies Preserve the Stability of Respiratory Chain Supercomplexes and Reduce Oxidative Stress in Reperfused Ischemic Hearts. Free Radic. Biol. Med..

[86] Smith J.A., Stallons L.J., Collier J.B., Chavin K.D., Schnellmann R.G. (2014). Suppression of Mitochondrial Biogenesis through Toll-like Receptor 4-Dependent Mitogen-Activated Protein Kinase Kinase/Extracellular Signal-Regulated Kinase Signaling in Endotoxin-Induced Acute Kidney Injury. J. Pharmacol. Exp. Ther..

[87] Aroor A.R., Mandavia C., Ren J., Sowers J.R., Pulakat L. (2012). Mitochondria and Oxidative Stress in the Cardiorenal Metabolic Syndrome. Cardiorenal Med..

[88] Peterson S.J., Choudhary A., Kalsi A.K., Zhao S., Alex R., Abraham N.G. (2020). Ox-Hdl: A Starring Role in Cardiorenal Syndrome and the Effects of Heme Oxygenase-1 Intervention. Diagnostics.

[89] Waldman M., Cohen K., Yadin D., Nudelman V., Gorfil D., Laniado-Schwartzman M., Kornwoski R., Aravot D., Abraham N.G., Arad M. (2018). Regulation of Diabetic Cardiomyopathy by Caloric Restriction Is Mediated by Intracellular Signaling Pathways Involving “SIRT1 and PGC-1α”. Cardiovasc. Diabetol..

