# Supplementary material for: NAC Pre-Administration Prevents Cardiac Mitochondrial Bioenergetics, Dynamics, Biogenesis, and Redox Alteration in Folic Acid-AKI-Induced Cardio-Renal Syndrome Type 3
Source: Antioxidants (Basel). 2023 Aug 10;12(8):1592. doi: 10.3390/antiox12081592 (PMC10451243; doi:10.3390/antiox12081592)

Supplementary material

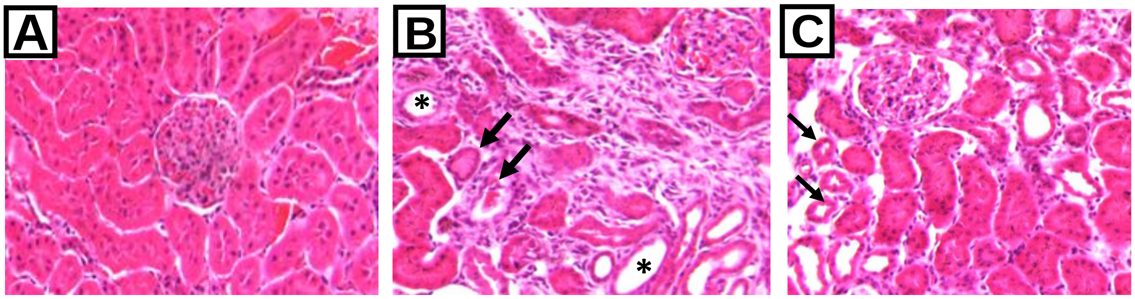

Figure S1. Representative micrographs of kidney sections with hematoxylin and eosin staining: (A) vehicle (normal histology). (B) FA group shows interstitial chronic inflammatory infiltrate surrounding proximal convoluted tubules, with swollen and detached epithelium (arrow) or hyaline cast in the lumen (arrow). Other tubules are coated with flat atrophic epithelium (asterisks). (C) NAC + FA group shows minimal interstitial inflammation and lesser tubular damage; some tubules show cuboidal cells with a hyperchromatic nucleus corresponding to the regenerative epithelium (arrows).

Table S1. General and heart parameters.

Parameter	Vehicle	FA	NAC+FA	NAC
Body weight (BW, g)	322 ± 37.3	259.2 ± 58.3*	225.8 ± 17.5*	261.3 ± 3.9
Heart weight (HW, g)	1.07 ± 0.16	1.04 ± 0.19	0.90 ± 0.09	0.97 ± 0.05
HW/BW (g/Kg)	3.3 ± 0.26	4.07 ± 0.59*	4.02 ± 0.37*	3.72 ± 0.09
Lung weight (LW, g)	2.2 ± 0.34	2.29 ± 0.45	1.5 ± 0.19*	1.9 ± 0.13
LW/BW (g/Kg)	7.07 ± 1.4	9.1 ± 2.02*	6.8 ± 0.69	7.4 ± 0.55
Tibial length (TL, cm)	5.12 ± 0.2	4.8 ± 0.29	4.8 ± 0.08*	5.2 ± 0.27
HW/TL (g/cm)	0.2 ± 0.03	0.21 ± 0.03	0.18 ± 0.02	0.18 ± 0.005
LW/TL (g/cm)	0.4 ± 0.07	0.48 ± 0.1	$0.31 \pm 0.03^{*,+}$	0.36 ± 0.04

*p≤0.05 vs. NAC. Data are mean ± SD n= 5-11. *p≤0.05 vs Control, +p≤0.05 vs. FA. FA = Folic acid, NAC = N-acetyl-cysteine.

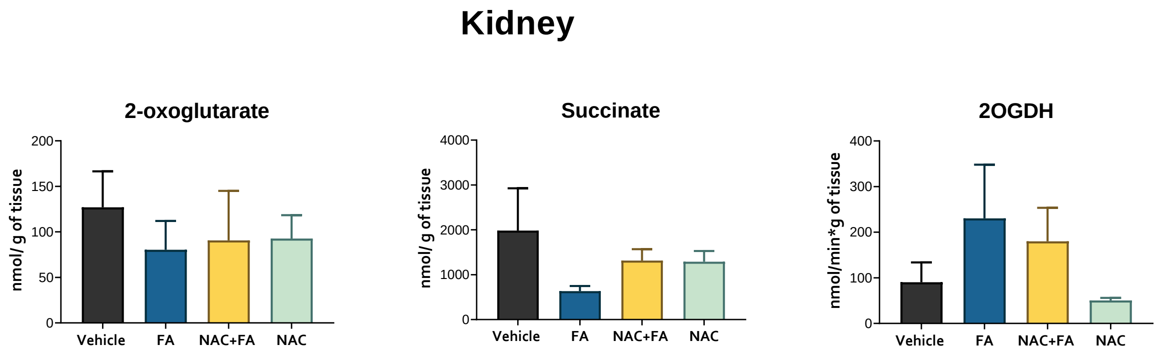

Figure S2. Levels of Krebs cycle intermediates 2-oxoglutarate and succinate and 2-oxoglutarate dehydrogenase (2-OGDH) activity in kidney homogenates. Data are mean ± SEM, n = 3. FA = Folic acid, NAC = N-acetyl-cysteine.

Heart mitochondrial H_2O_2 production

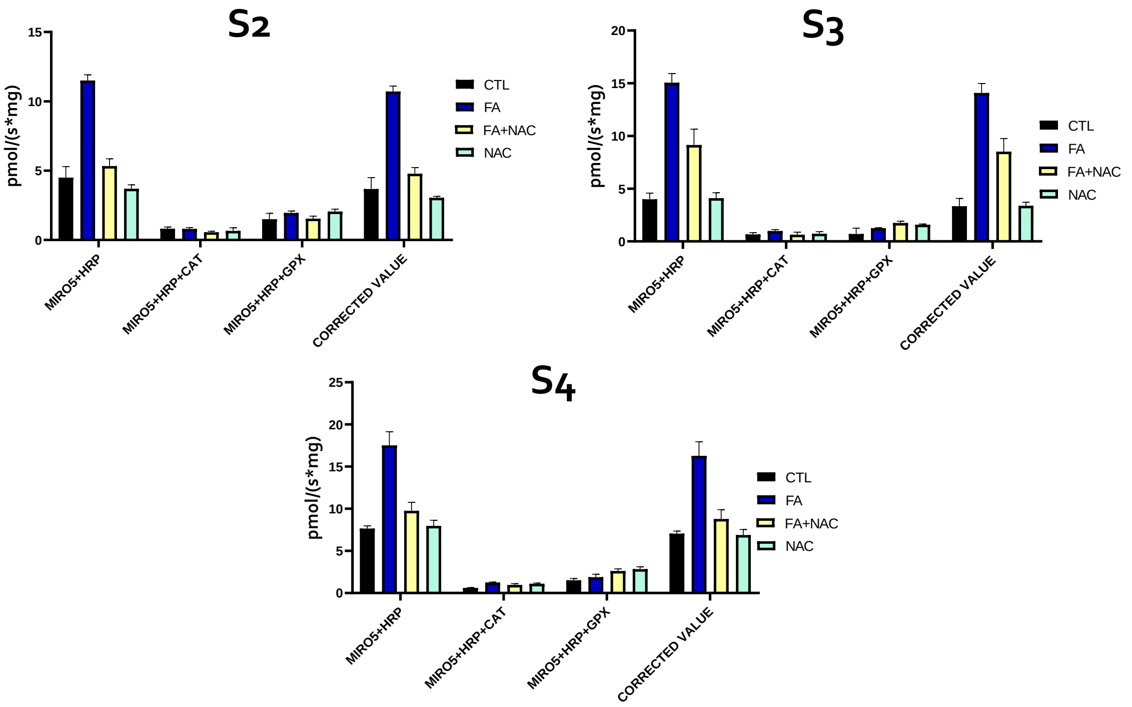

Figure S3. Evaluation of the rate of mitochondrial hydrogen peroxide (H_2O_2) production using Amplex red as a probe in the respiratory states S2= State 2, S3= State 3, S4= State 4. Data are mean ± SEM, n = 3. FA = Folic Acid, NAC = N-acetyl-cysteine. Firstly, freshly isolated mitochondria were evaluated in Mitochondrial Respiration Buffer 05 plus Horseradish peroxidase 0.5 U/Ml (MIR05+HRP). H_2O_2 oxidizes Amplex red in the presence of HRP to produce the fluorescence product resorufin (λ=530–590 nm). The activity was expressed as nmol/min/mg of protein. To eliminate the verified specificity of the probe to H_2O_2, a second assay using the same condition in medium MIR05+HRP plus 280 U/mL of catalase (MIR05+HRP+CAT) was run for each group. As is observed in the figure, the H_2O_2 scavenger, by catalase addition, drastically decreases the signal observed in all respiratory states and groups, proving that the observed signal is mainly attributed to this ROS. Similar results were observed in different assays where the base medium MIR05+HRP was supplemented with glutathione peroxidase and reduced glutathione (MIR05+HRP+GPx groups), reaffirming that the observed signal is mainly attributed to H_2O_2. The corrected value was determined as (MIR05+HRP) $_{activity}$ - (MIR05+HRP+CAT) $_{activity}$ and was reported in Fig. 7 of the results section.

Mitochondrial fraction

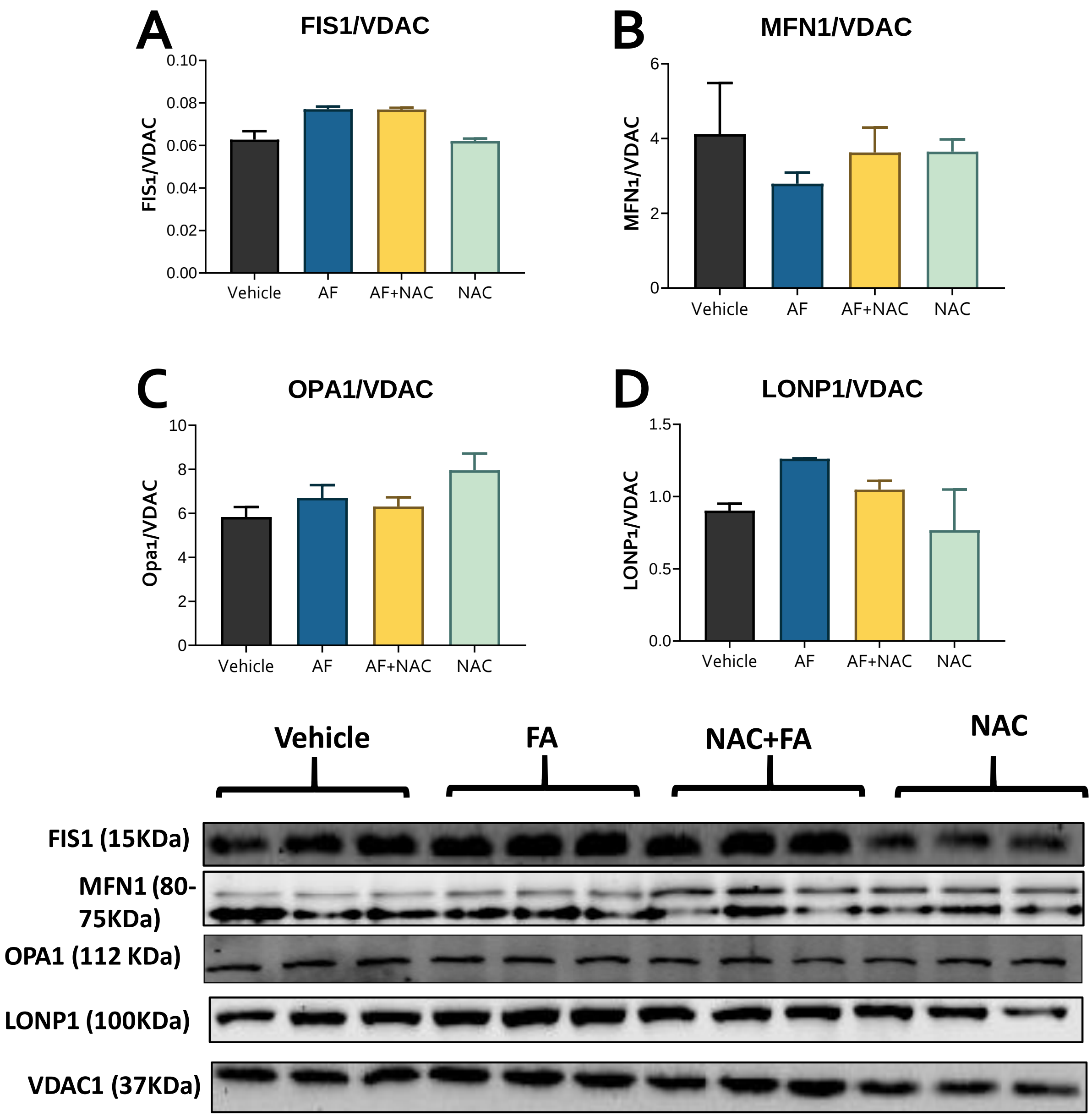

Figure S4. Western proteins in heart isolated mitochondrial and their densitometries of fission proteins (A) FIS1; fusion proteins: (B) MFN1 and (C) OPA1; as well as mitochondrial protease (D) LONP1. Data are mean ± SEM, n = 3. FIS1= Mitochondrial fission 1 protein, VDAC= Voltage-dependent Anion selective channel, MFN1= Mitofusin 1, , LONP1= Lon Peptidase 1 mitochondrial, OPA1= Optic Atrophy 1. FA = Folic Acid, NAC = N-acetyl-cysteine

Western blot membranes of Figure 1.

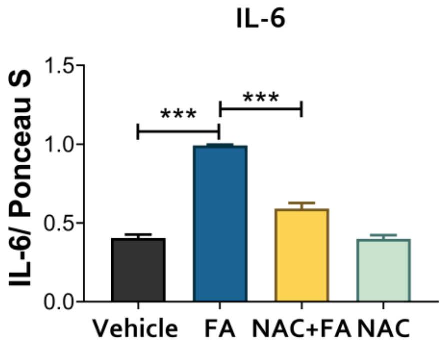

Vehicle	FA	NAC+FA	NAC
0.467	1	0.502	0.495
0.428	0.966	0.556	0.442
0.463	0.998	0.483	0.366
0.356	0.995	0.685	0.369
0.357	1	0.68	0.369
0.358	0.994	0.634	0.354

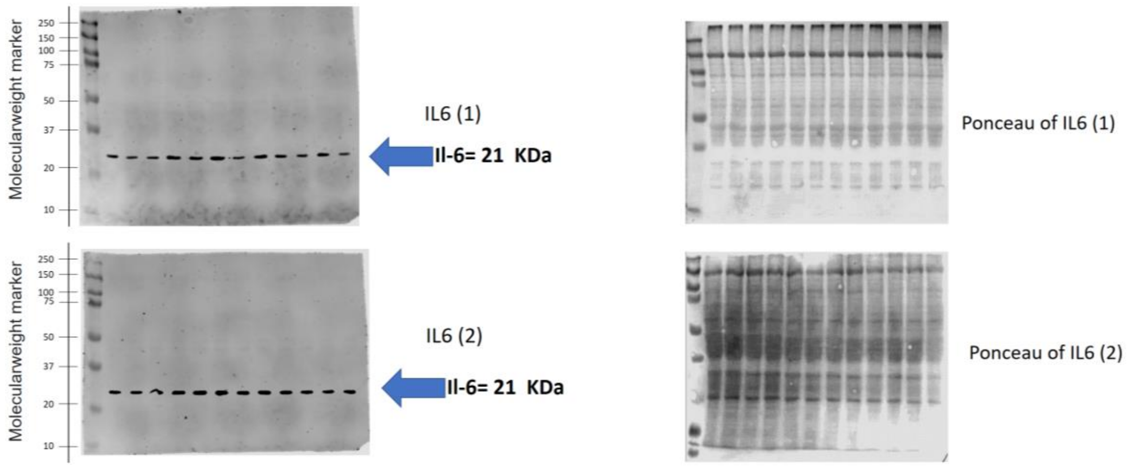

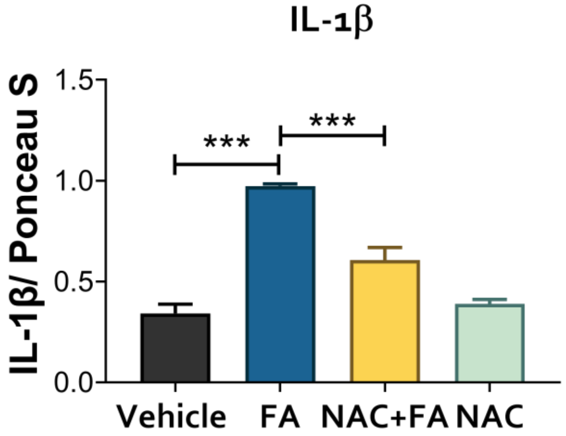

Vehicle	FA	NAC+FA	NAC
0.25	0.94	0.44	0.37
0.24	0.95	0.49	0.31
0.23	1	0.48	0.34
0.44	0.99	0.73	0.44
0.44	0.96	0.75	0.43
0.45	1	0.75	0.44

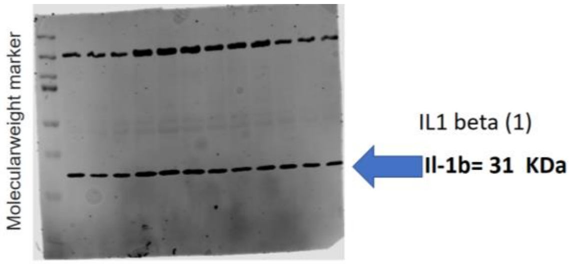

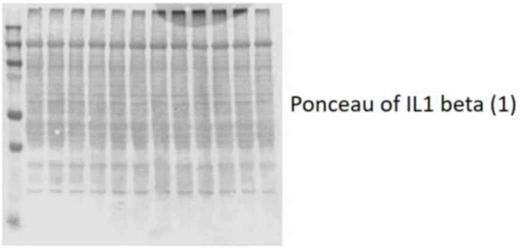

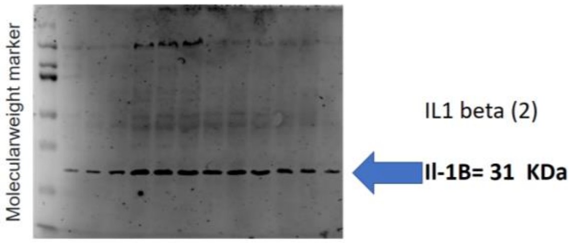

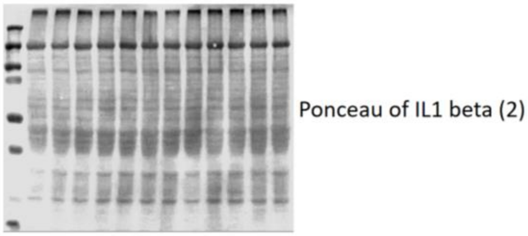

Western blot membranes of Figure 2.

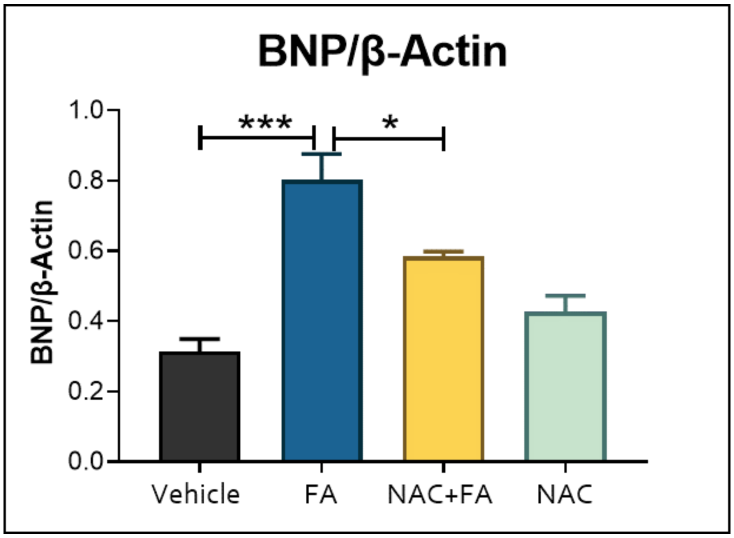

Vehicle	FA	NAC+FA	NAC
0.30022826	0.65301131	0.55928677	0.35596873
0.25948604	0.86397149	0.59080572	0.41116443
0.38072393	0.88862752	0.60434262	0.51242124

BNP

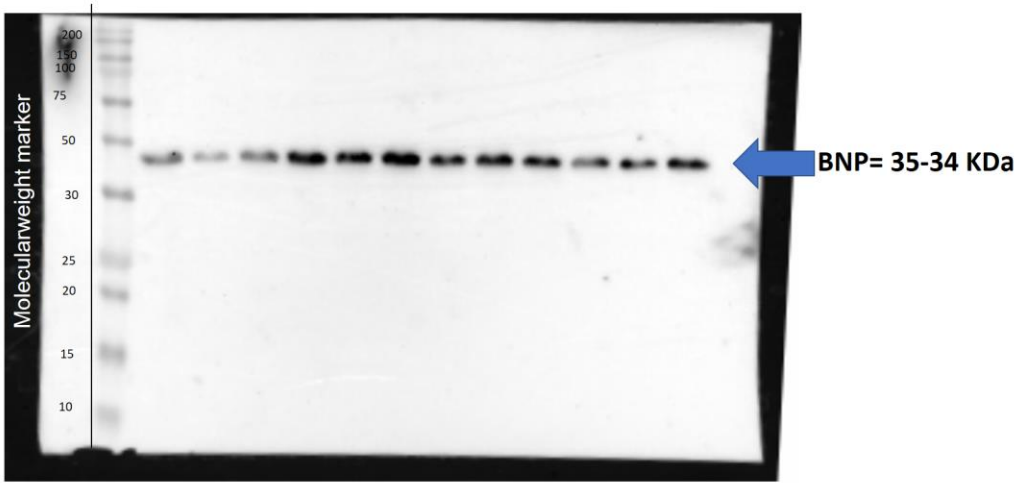

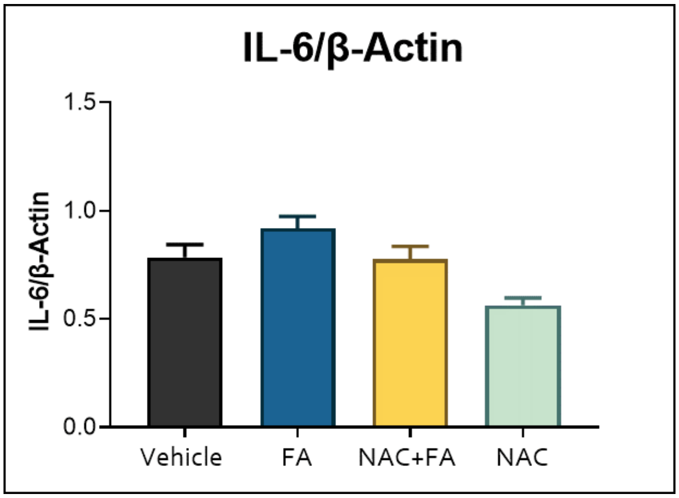

Vehicle	FA	NAC+FA	NAC
0.90502479	1.02760273	0.79765069	0.49364511
0.71897558	0.8554978	0.66333741	0.59936652
0.72651235	0.87514385	0.86692549	0.5947087

IL-6

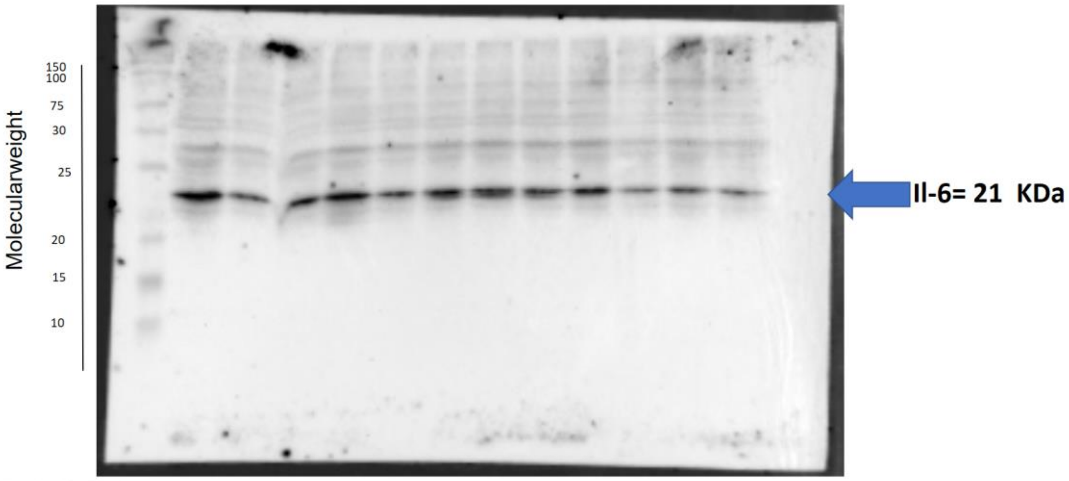

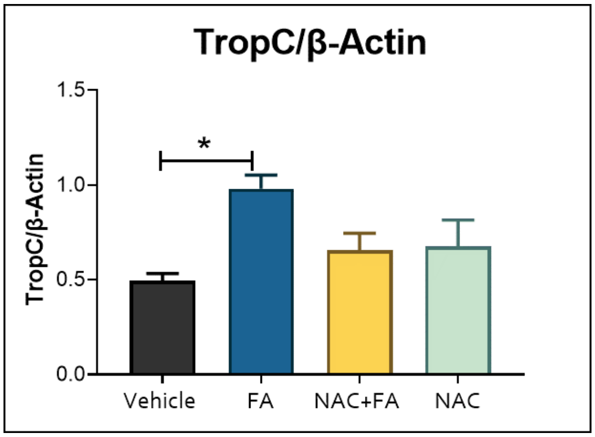

Vehicle	FA	NAC+FA	NAC
0.56303901	0.96069144	0.56370764	0.94998611
0.42640581	0.86728514	0.83751261	0.50026708
0.49155765	1.11400709	0.56246518	0.57844585

Troponin C

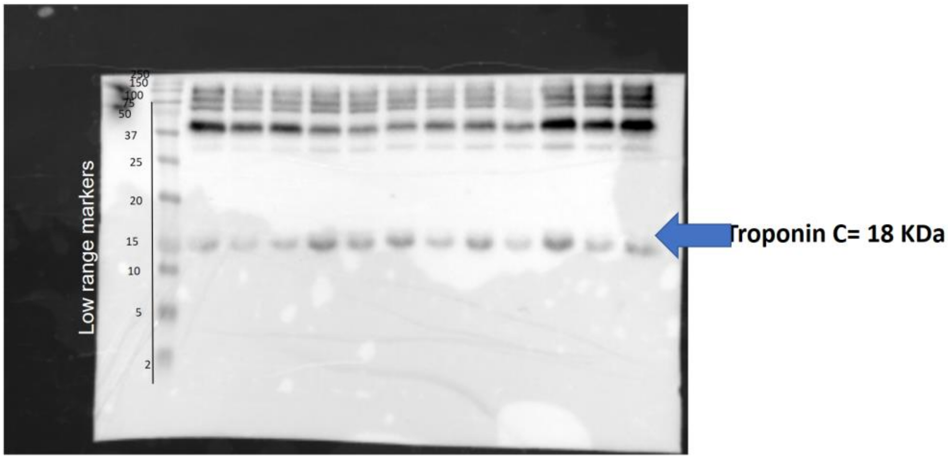

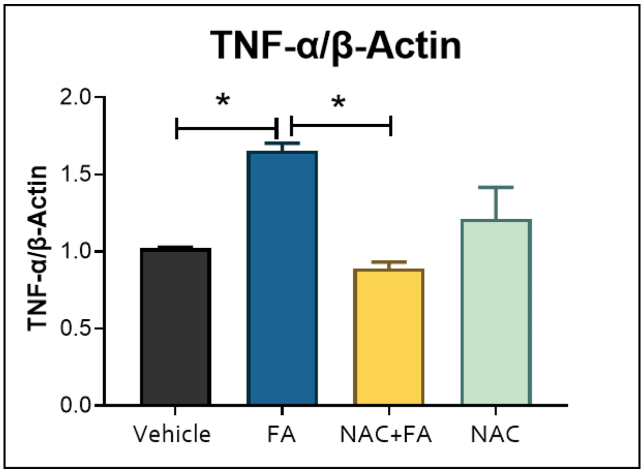

Vehicle	FA	NAC+FA	NAC
1.03164799	1.5612415	0.88301117	1.61052786
1.01988676	1.7154448	0.82209546	0.93107211
1.00947436	1.68885191	0.96622058	1.09394663

TNF alfa

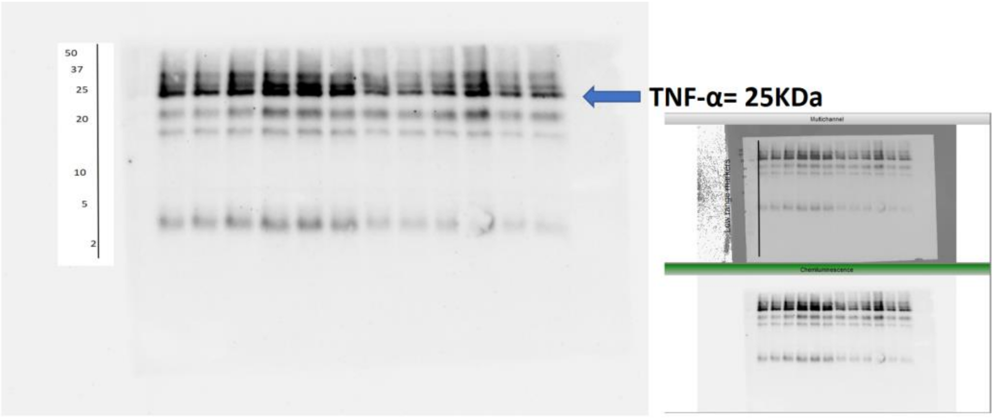

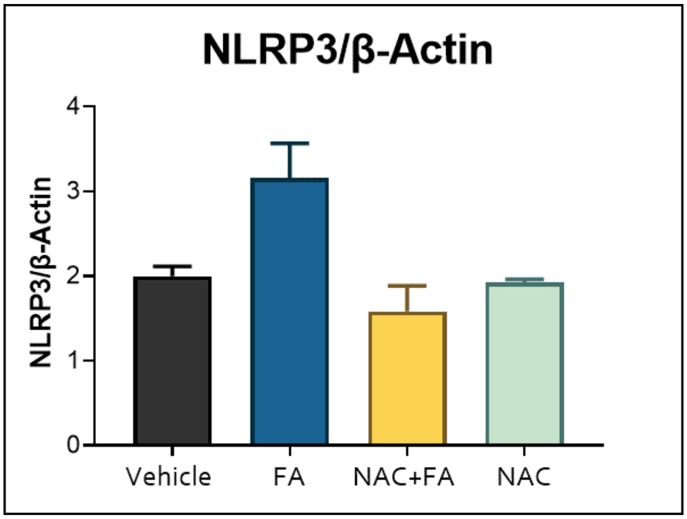

Vehicle	FA	NAC+FA	NAC
1.94946642	2.33854585	2.1586361	1.99970225
2.21920527	3.64537073	1.45860914	1.87062005
1.82593792	3.48331854	1.12861024	1.89159324

NLRP3

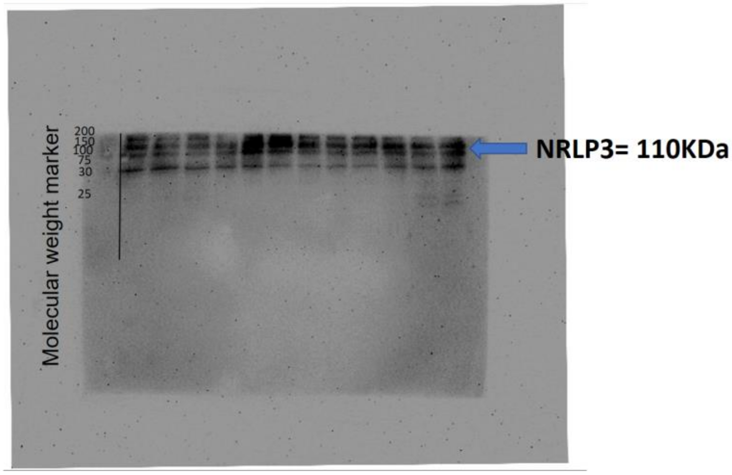

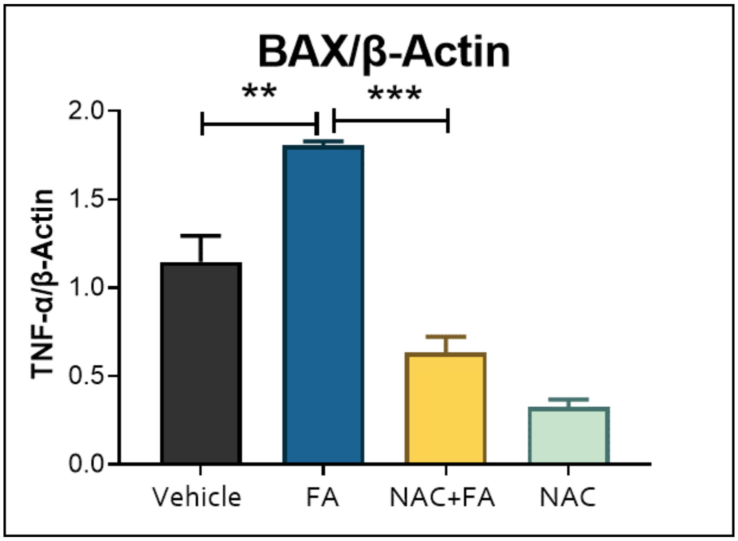

Vehicle	FA	NAC+FA	NAC
0.85495728	1.83219605	0.62880947	0.24475559
1.25033148	1.82616462	0.48028205	0.39058801
1.33393157	1.75600981	0.79027065	0.33718414

BAX

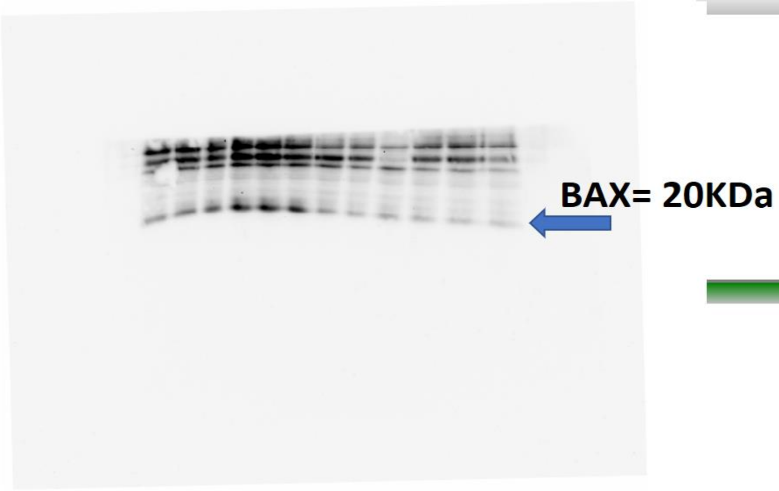

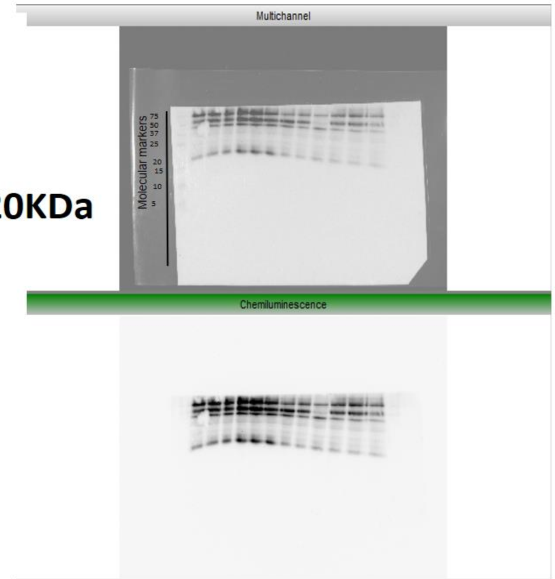

B-actin

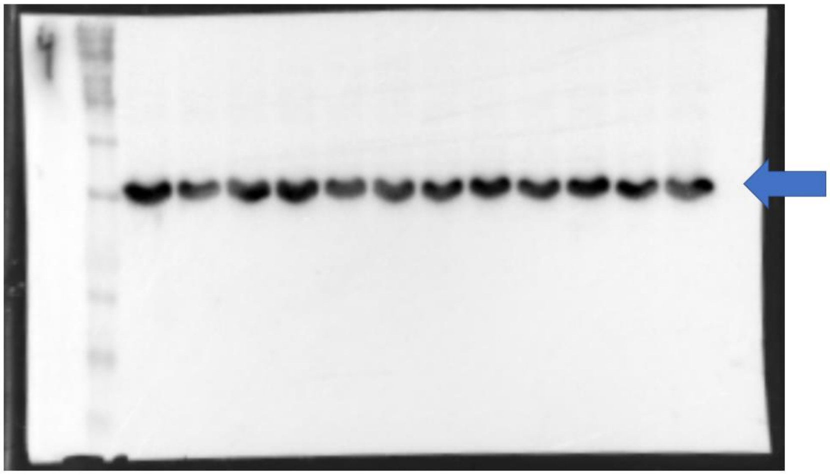

B actin= 43 KDa

Western blot membranes of Figure 7.

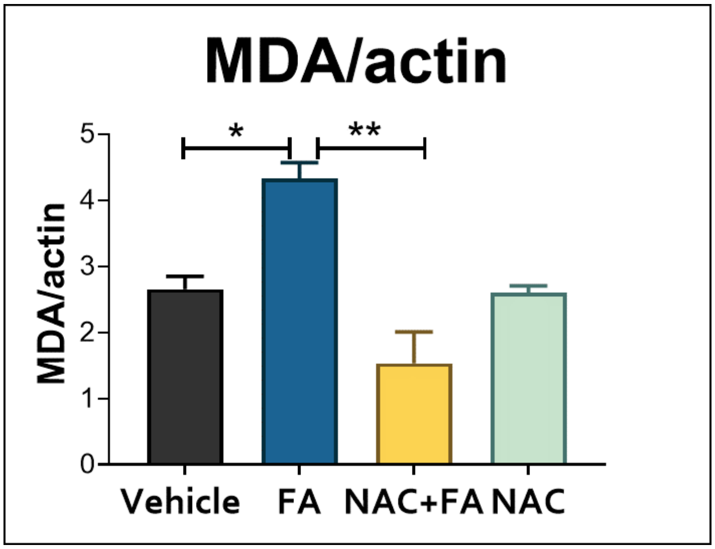

Vehicle	FA	NAC+FA	NAC
2.937215	4.026724	1.198199	2.600187
2.747233	4.171483	2.476323	2.779822
2.277246	4.799113	0.91936	2.415789

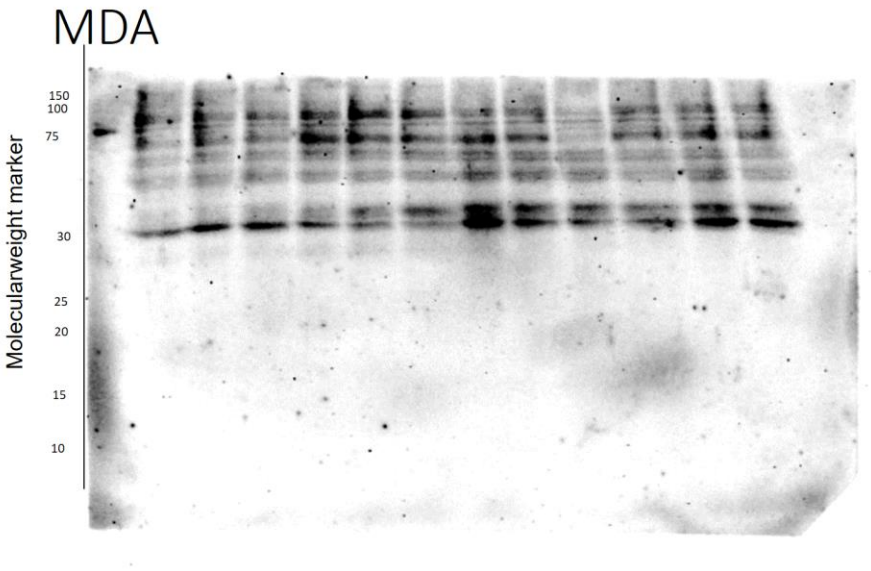

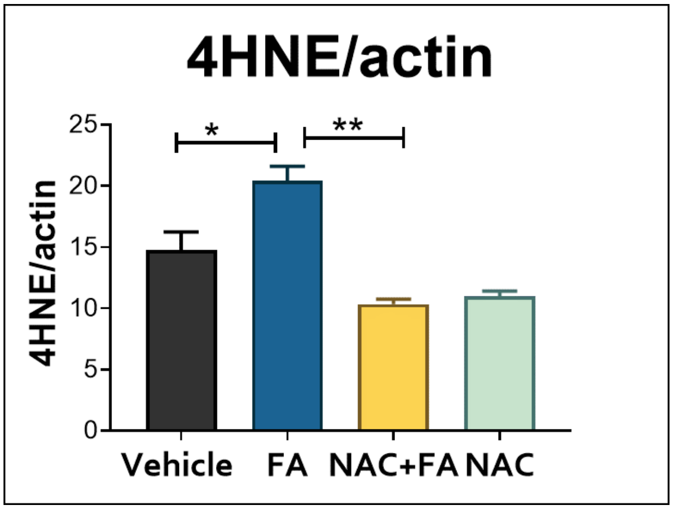

Vehicle	FA	NAC+FA	NAC
17.42864	19.09086	9.864676	10.1184
14.53852	19.33127	11.1409	11.26596
12.25064	22.79039	9.99134	11.50833

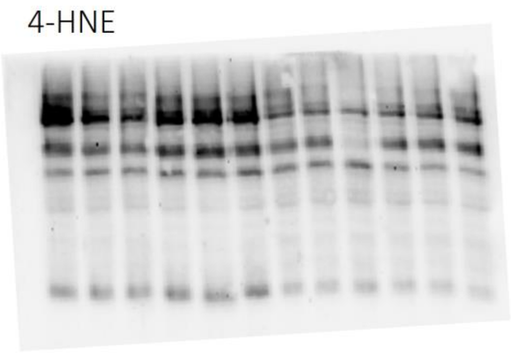

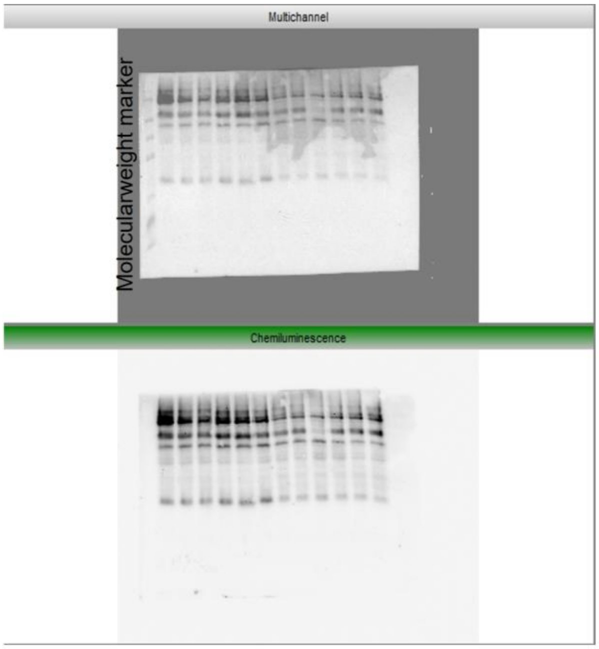

B- Actin

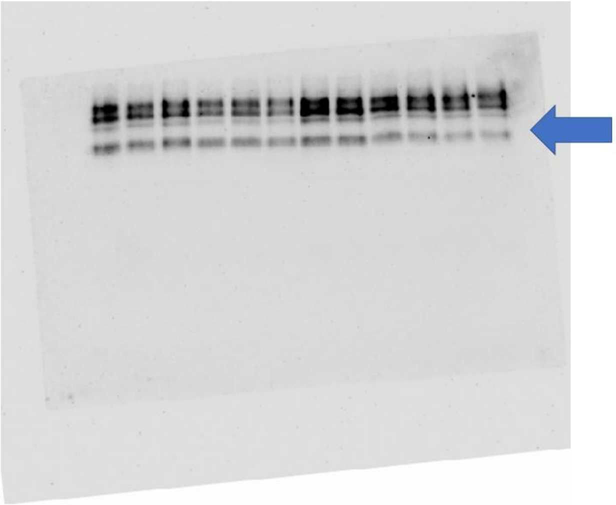

B actin= 43 KDa

Western blot membranes of Figure 9

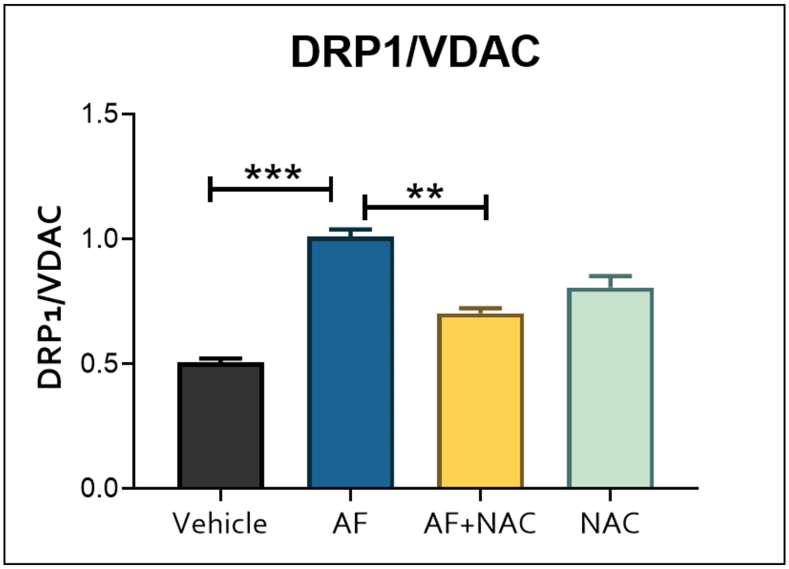

Vehicle	AF	AF+NAC	NAC
0.49965369	1.03320243	0.67330984	0.71289989
0.4847872	1.04291468	0.6927488	0.84842755
0.53533162	0.95023144	0.74112967	0.85477446

DRP1

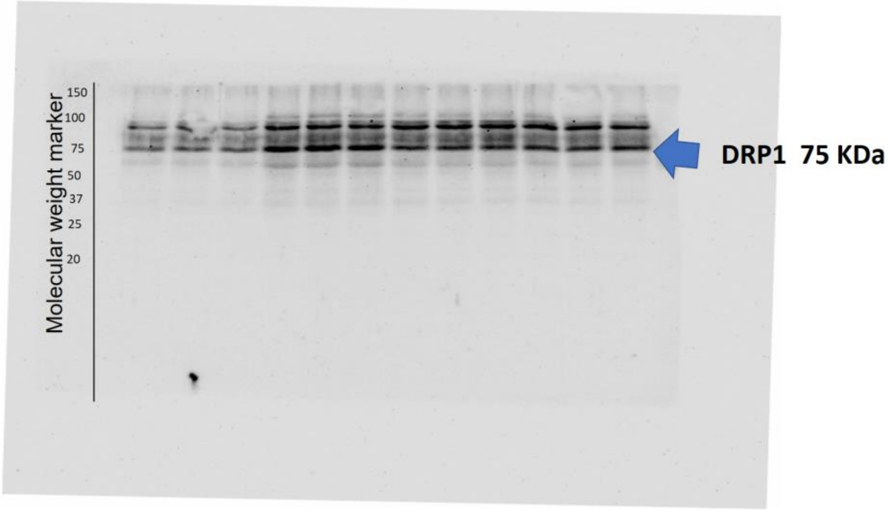

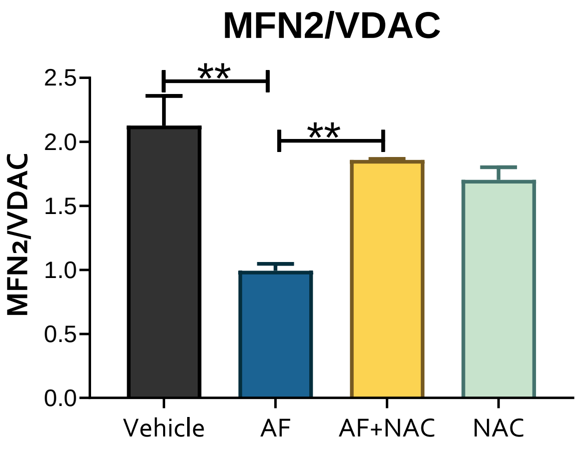

Vehicle	AF	AF+NAC	NAC
0.3789049	0.63578036	2.02428978	1.11740291
0.73219723	0.86560829	1.55301633	1.1770406
0.35561275	0.78531569	1.32767801	0.97287765

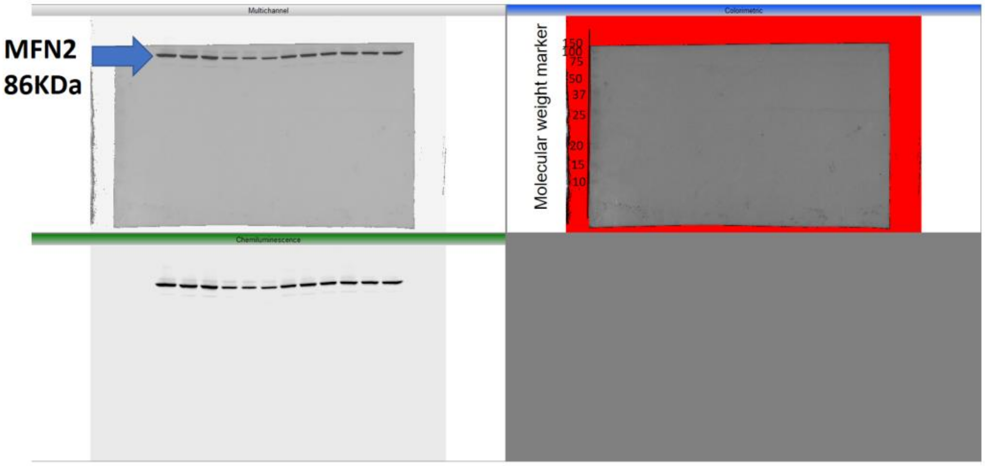

VDAC

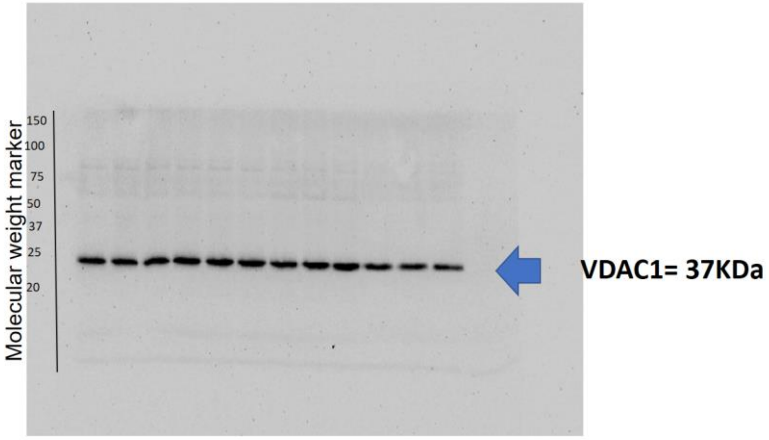

Western blot supelemtary figure 4

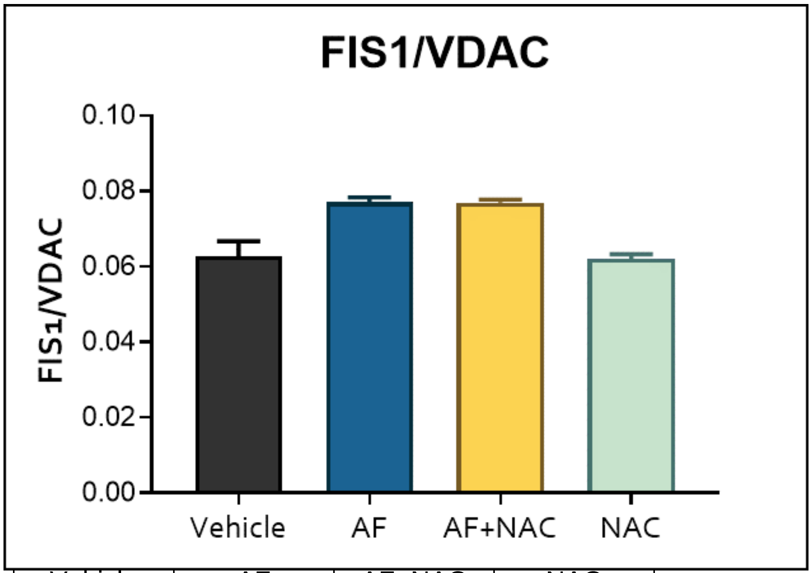

Vehicle	AF	AF+NAC	NAC
0.05593013	0.07911003	0.0779793	0.06192357
0.06958683	0.07471382	0.07749142	0.06413311
0.06277675	0.07733455	0.07517437	0.06005298

Fis1

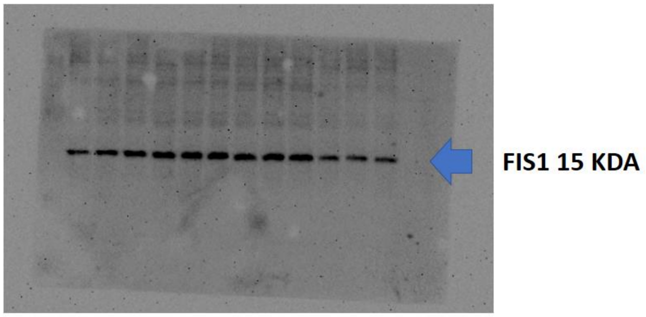

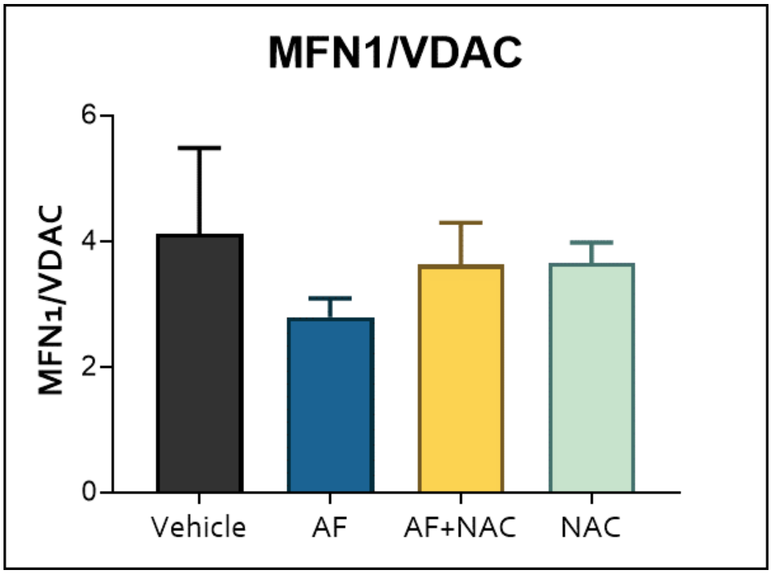

Vehicle	AF	AF+NAC	NAC
6.796167	2.584477	2.980436	3.477387
2.316114	3.382156	4.959867	4.28662
3.238759	2.405699	2.940465	3.189984

MFN1

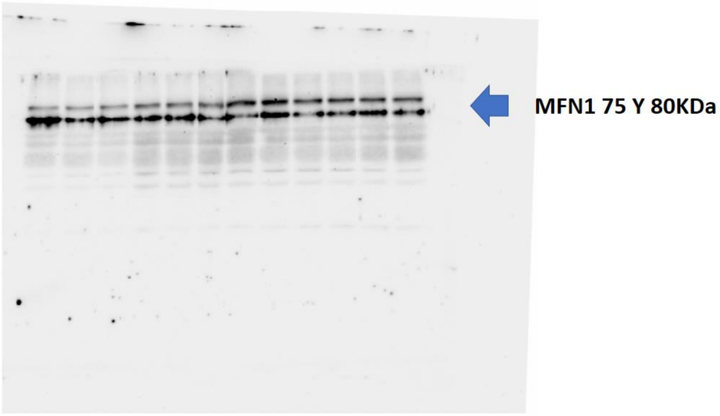

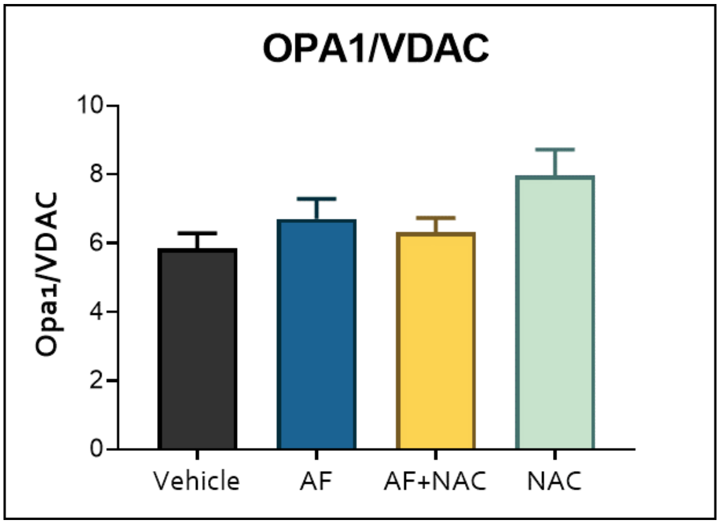

Vehicle	AF	AF+NAC	NAC
5.283835	7.672334	5.570879	9.045065
5.479125	5.660518	7.038251	8.334301
6.735062	6.776542	6.319103	6.484178

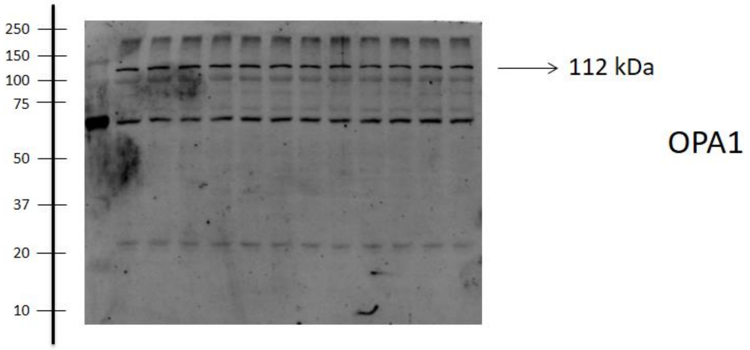

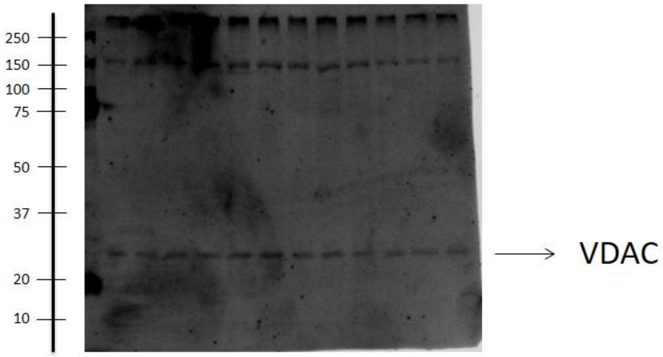

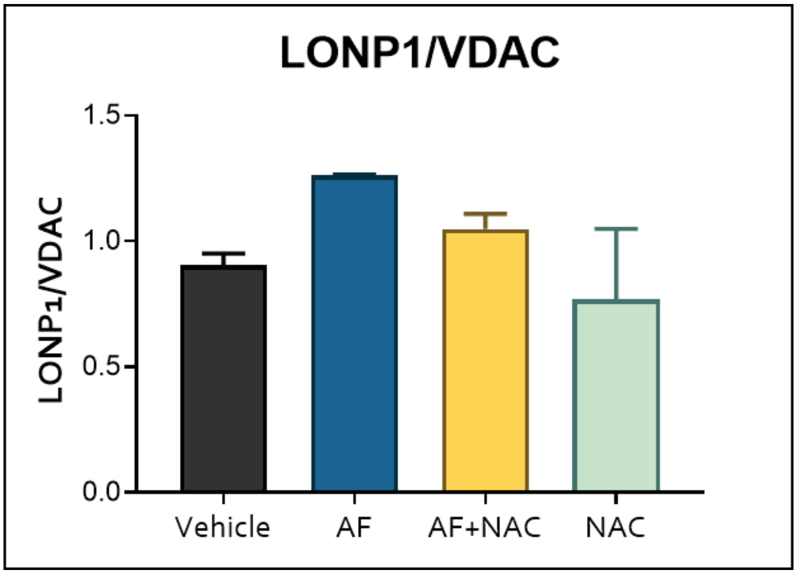

Vehicle	AF	AF+NAC	NAC
0.80842402	1.26166443	0.99053442	0.97640648
0.94509542	1.25733501	1.16898178	1.11613914
0.9550785	1.26730732	0.98437941	0.20708973

LONP

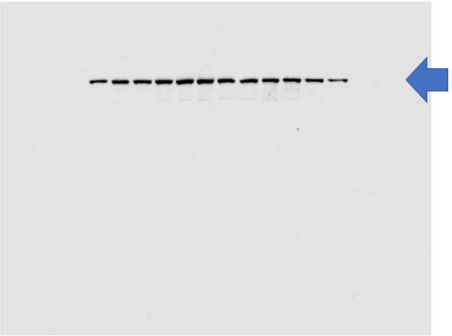

LONP 100KDA

Western blot membranes of Figure 11.

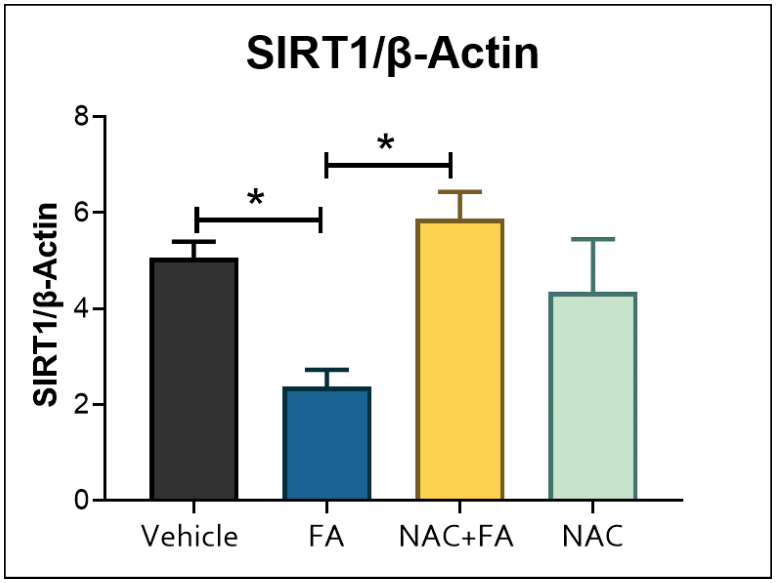

Vehicle	FA	NAC+FA	NAC
5.52415512	3.03380195	5.46337493	5.76550045
5.24777932	2.26876459	6.973627	2.16927752
4.41229537	1.82952411	5.18604555	5.08827623

SIRT1

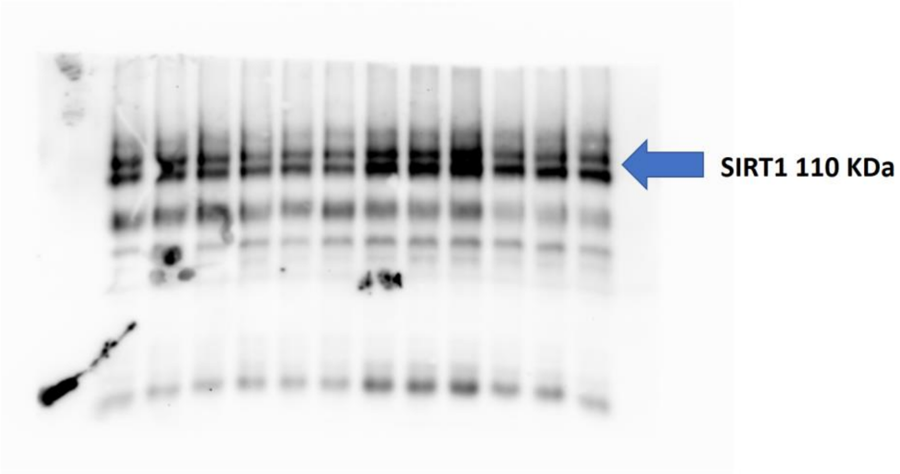

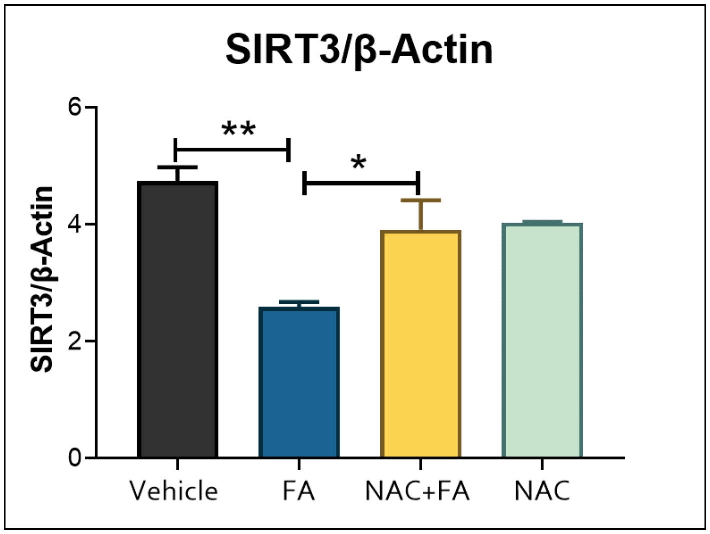

Vehicle	FA	NAC+FA	NAC
5.21209779	2.68983826	3.88457935	4.03808888
4.47368897	2.65913419	3.04511235	4.01417205
4.52635123	2.41276779	4.79260803	4.04564588

Sirt3

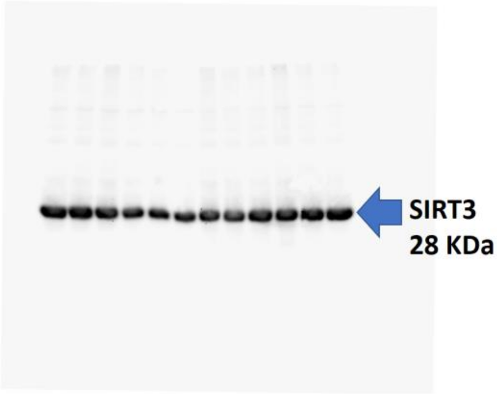

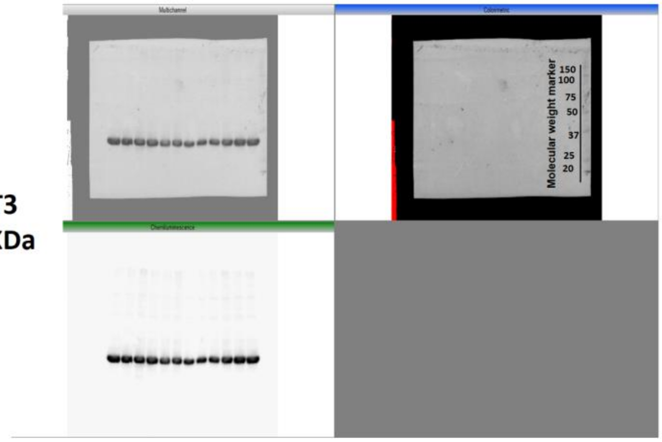

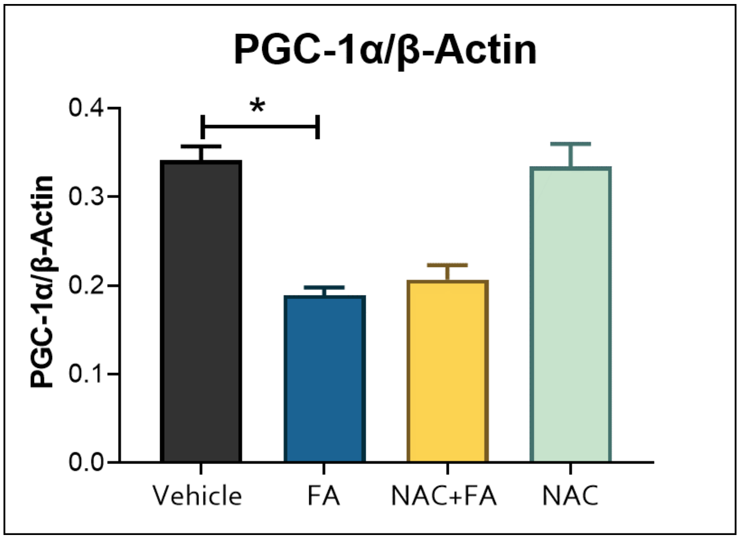

Vehicle	FA	NAC+FA	NAC
0.30963461	0.18414456	0.20347402	0.3849452
0.35657256	0.17763982	0.23651442	0.30199329
0.35713431	0.20608746	0.18041365	0.31545666

PGC-1α

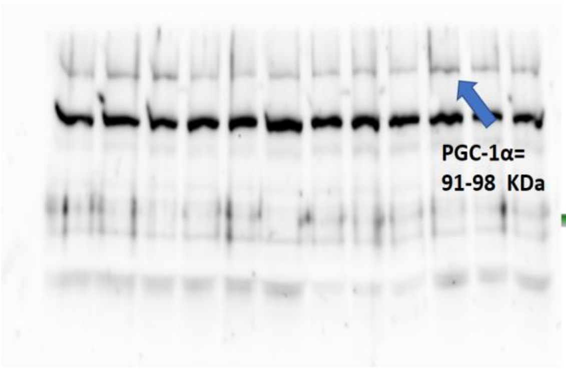

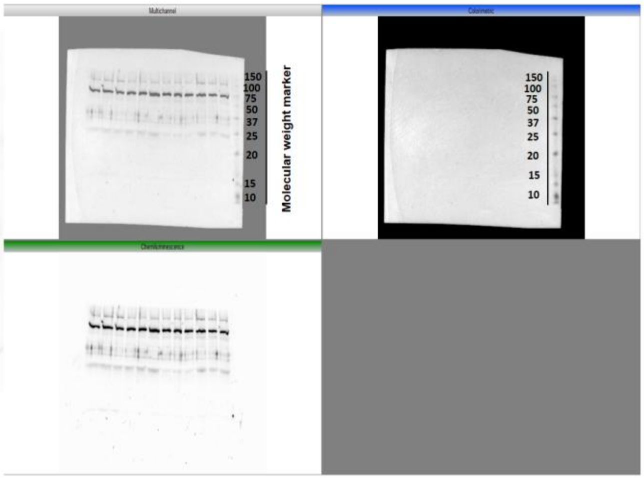

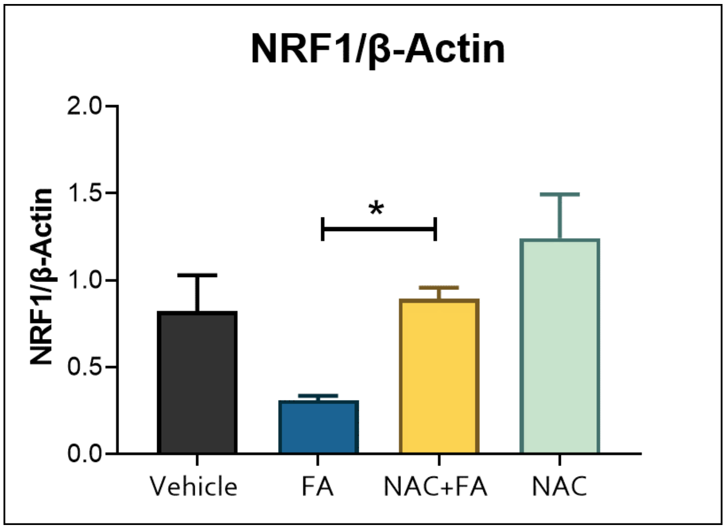

Vehicle	FA	NAC+FA	NAC
1.23415707	0.32171404	0.99463225	0.82935667
0.57739134	0.26279176	0.77704334	1.69827541
0.65307219	0.34810109	0.90904736	1.19751918

NRF1

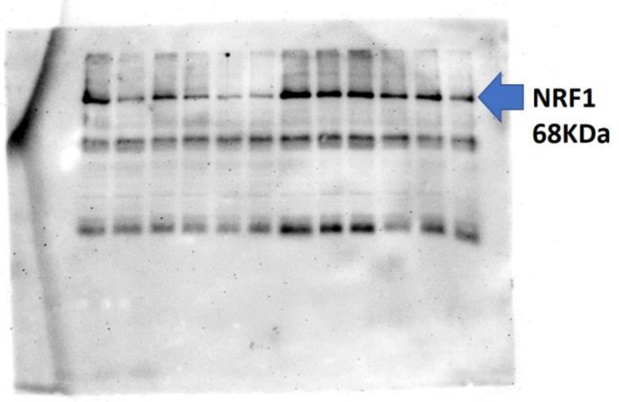

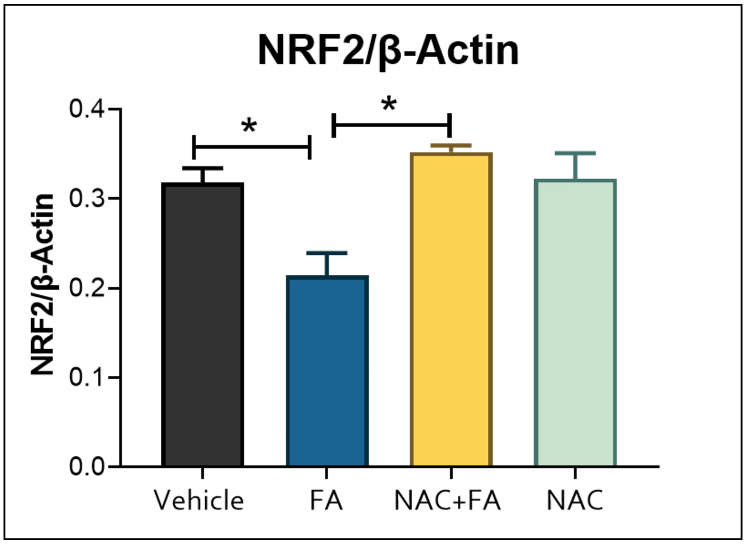

Vehicle	FA	NAC+FA	NAC
0.345182	0.235862	0.362289	0.322044
0.318965	0.242411	0.356112	0.273214
0.290114	0.164457	0.33754	0.372021

NRF2

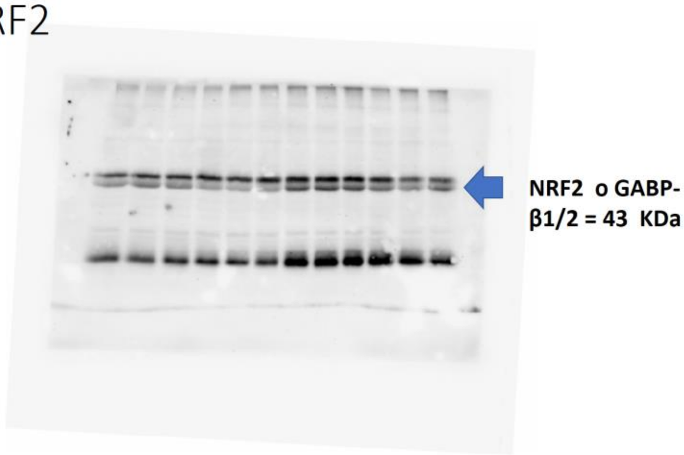

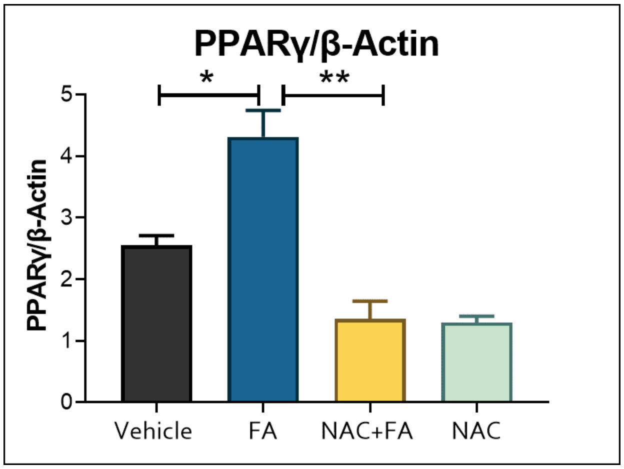

Vehicle	FA	NAC+FA	NAC
2.23351941	4.88662656	0.91516011	1.47518327
2.69654108	4.57561357	1.24968928	1.29423187
2.71883719	3.47627225	1.90003155	1.12690707

PPAR gamma

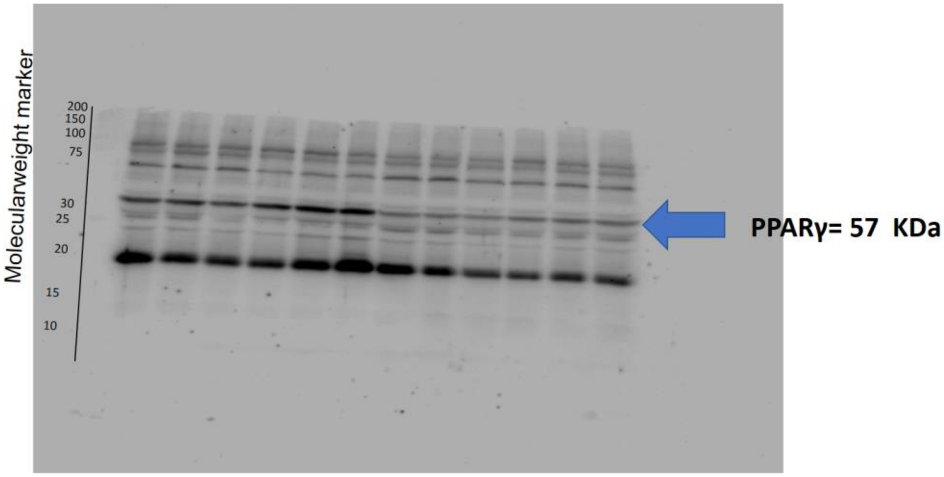

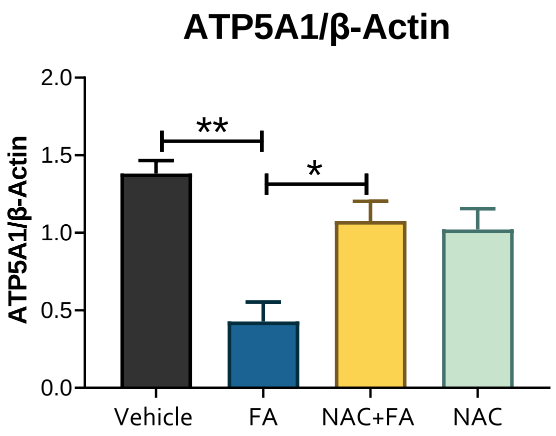

Vehicle	FA	NAC+FA	NAC
1.359236	0.333929	1.329114	1.131876
1.246283	0.274222	0.970024	1.17835
1.53816	0.675813	0.929425	0.751162

ATP5a

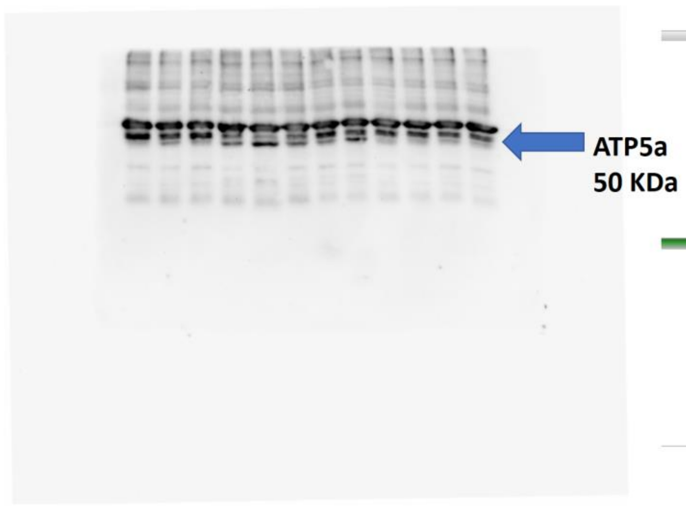

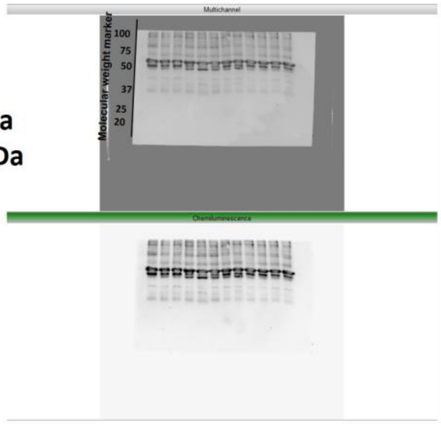

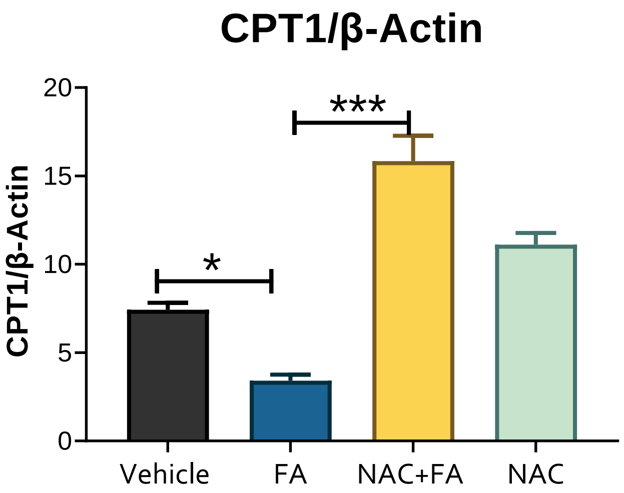

Vehicle	FA	NAC+FA	NAC
7.006263	2.922048	13.23143	10.36047
8.222851	4.066357	18.14552	12.42093
7.035095	3.233778	16.14807	10.57153

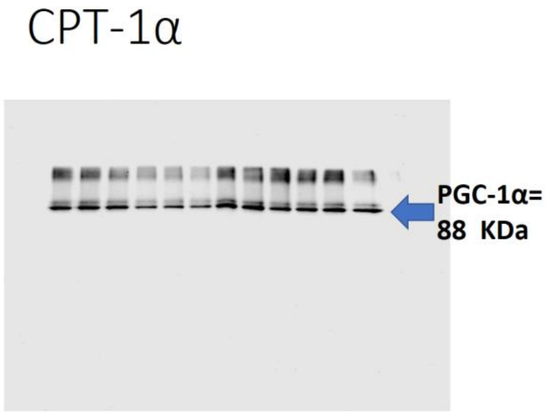

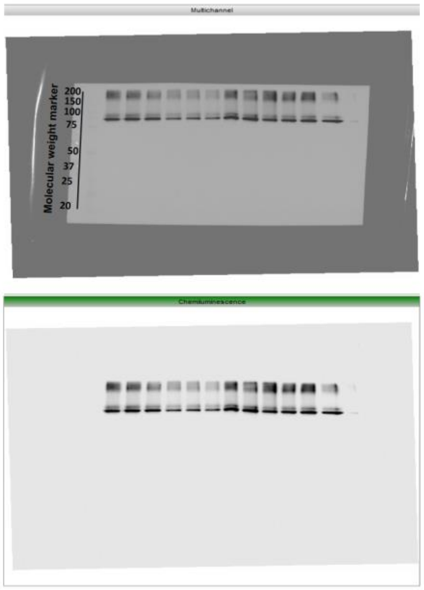

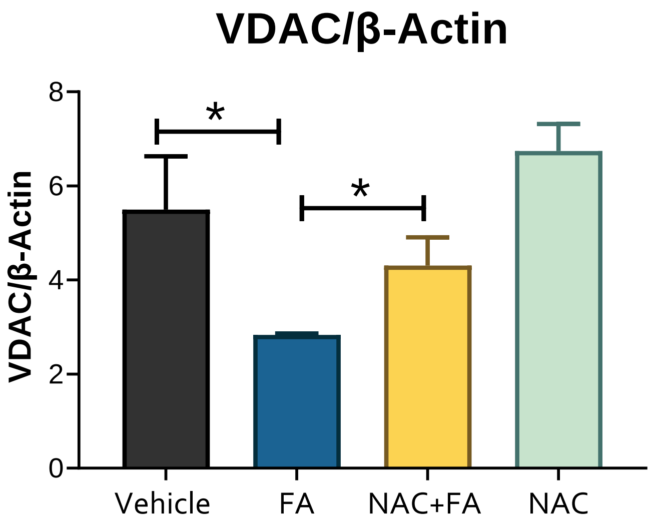

Vehicle	FA	NAC+FA	NAC
7.76972752	2.87445045	3.96595681	5.63988016
4.38507927	2.77525568	3.47869177	7.00790834
4.3299294	2.85065469	5.47437846	7.57698683

VDAC

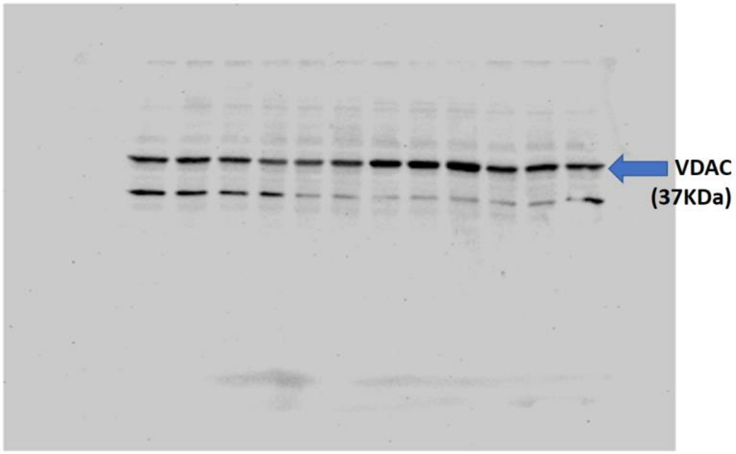

β-Actin

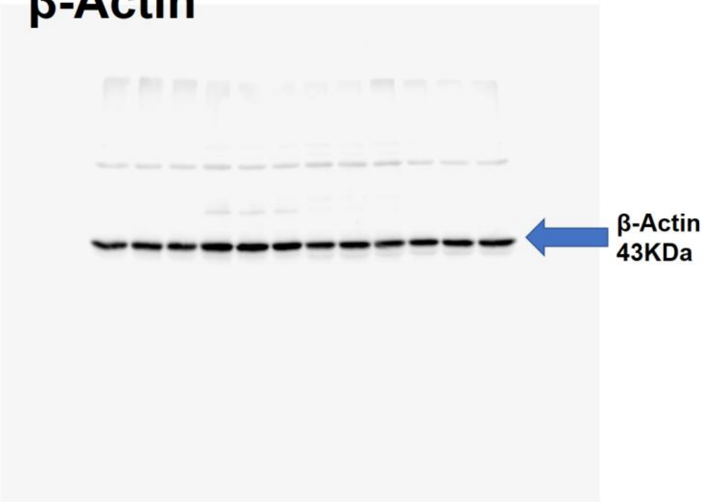

Western blot membranes of Figure 12.

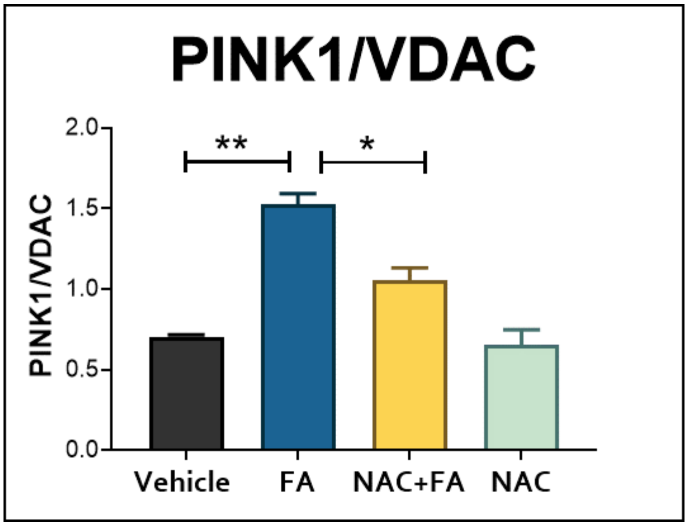

Vehicle	FA	NAC+FA	NAC
0.682705	1.467966	1.076935	0.842651
0.723144	1.65801	0.915936	0.539535
0.708236	1.459375	1.17498474	0.571308

PINK

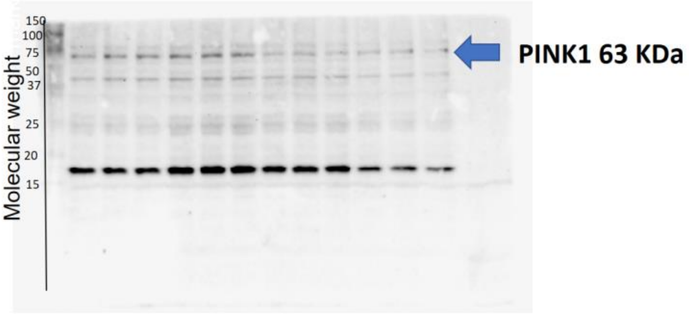

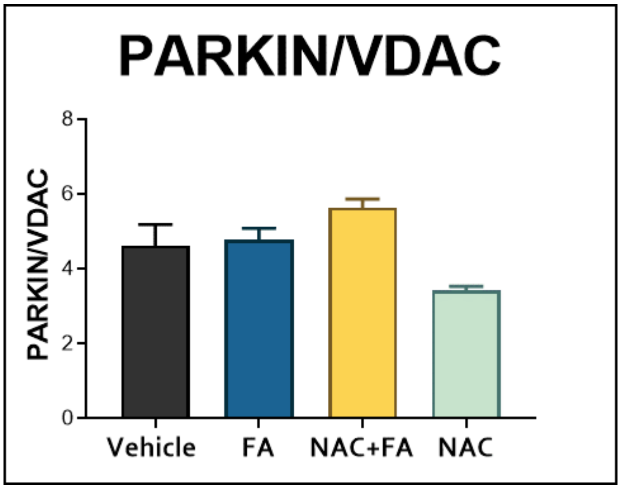

Vehicle	FA	NAC+FA	NAC
3.607509	4.479805	5.765629	3.449712
5.558215	4.460144	5.191758	3.589188
4.681846	5.382733	5.944185	3.209564

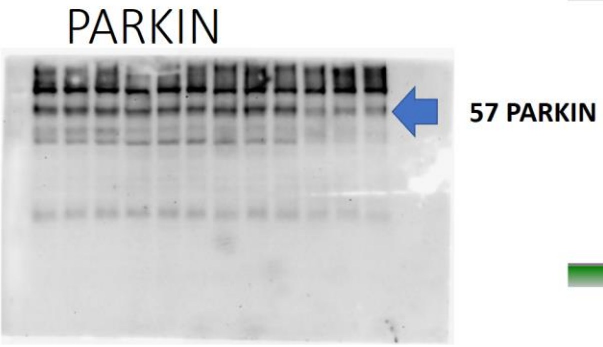

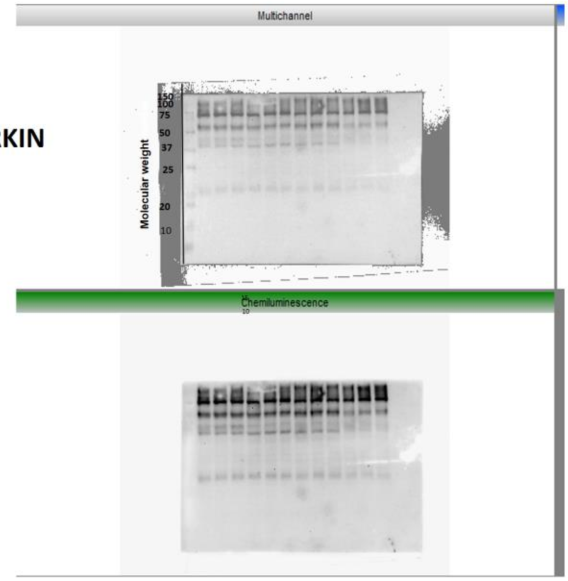

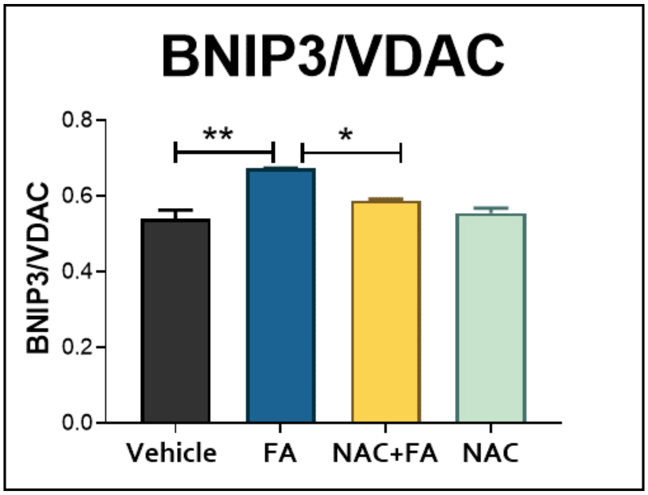

Vehicle	FA	NAC+FA	NAC
0.58681384	0.67337381	0.58102964	0.57398213
0.52409407	0.67200514	0.59390857	0.53203779
0.50873734	0.67461331	0.59162463	0.56174891

BNIP3

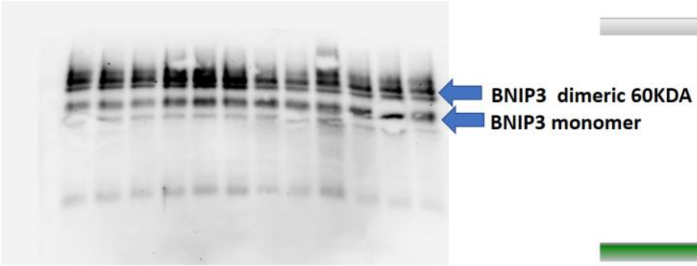

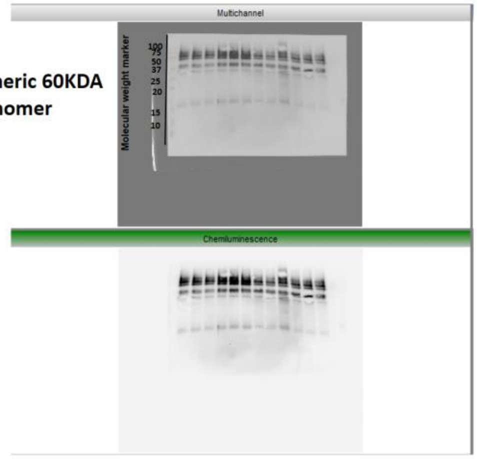

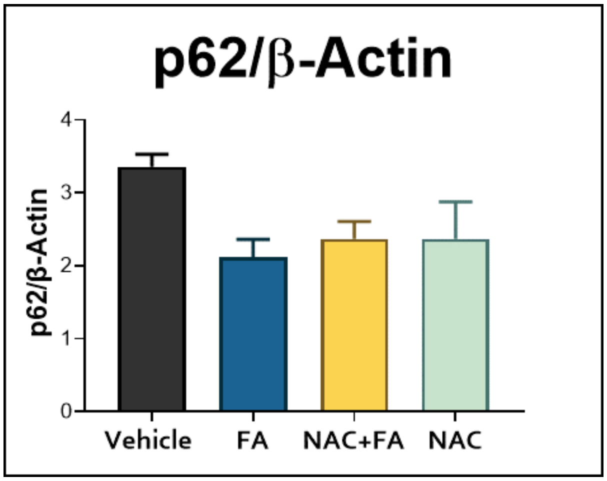

Vehicle	FA	NAC+FA	NAC
3.617965	2.14913844	1.998233	1.90859241
3.418165	2.52571216	2.286647	3.38054665
3.03584923	1.68650581	2.813905	1.81502106

p62

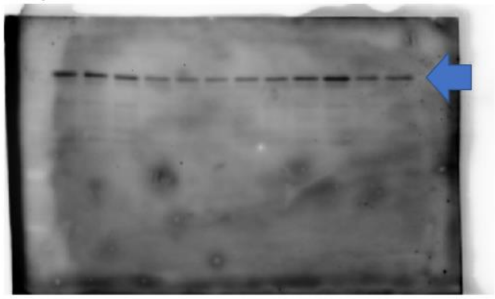

P62 60-62kda

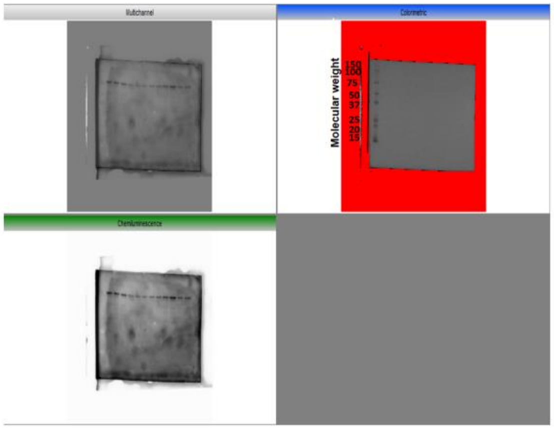

VDAC mitochondrial fraction

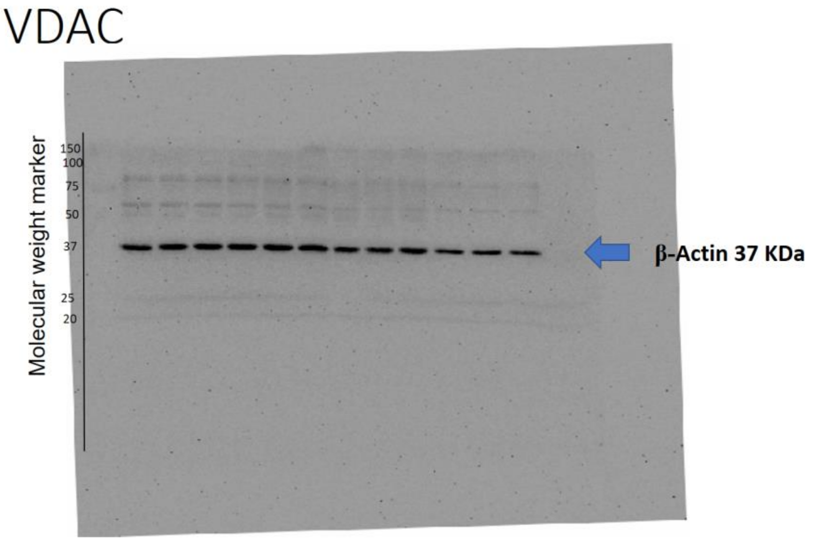

B-actin heart homogenate

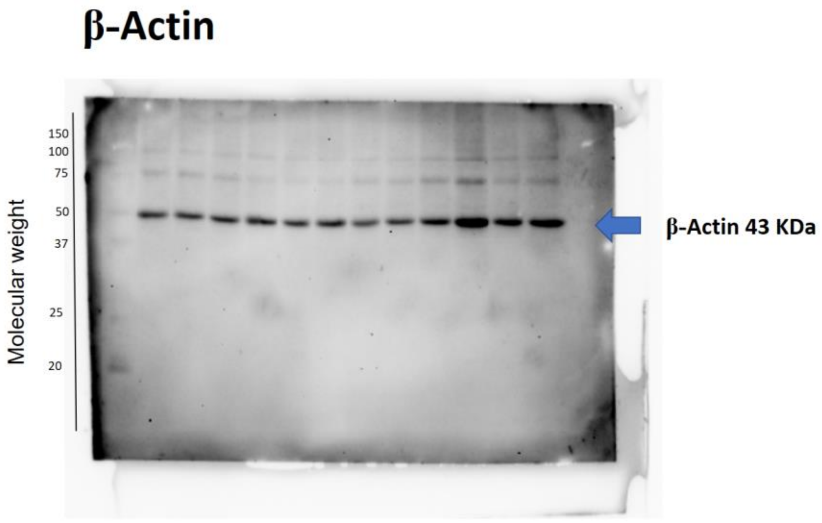

Supplement: Supplementary file 1 [file antioxidants-12-01592-s001.zip › antioxidants-2503762-supplementary.pdf]
